# Amphiphilic Triazine Polymer Derivatives as Antibacterial And Anti-atopic Agents in Mice Model

**DOI:** 10.1038/s41598-019-51561-7

**Published:** 2019-10-22

**Authors:** Pethaiah Gunasekaran, meiqi Fan, Eun Young Kim, Jun Ho Shin, Ji Eun Lee, Eun Ju Son, Jaehi Kim, Eunha Hwang, Min Su Yim, Eun-Hee Kim, Young-Jin Choi, Young-Ho Lee, Young-Ho Chung, Hak Nam Kim, Eun Kyoung Ryu, Song Yub Shin, Eun-Kyung Kim, Jeong Kyu Bang

**Affiliations:** 1 Division of Magnetic Resonance, Korea Basic Science Institute (KBSI), Ochang, Chung Buk 28119 Republic of Korea; 2 Division of Food Bioscience, Konkuk University, Chungju, 27478 Republic of Korea; 3 Department of Medical Science, Graduate School, and Department of Cellular and Molecular Medicine, School of Medicine, Chosun University, Gwangju, 61452 Republic of Korea; 4 Drug & Disease Target Research Team, Korea Basic Science Institute (KBSI), Ochang, Chung Buk 28119 Republic of Korea; 5 Department of Bio-analytical Science, University of Science & Technology, Daejeon, 34113 Republic of Korea

**Keywords:** Chemical libraries, Small molecules

## Abstract

Considering the emergence of bacterial resistance and low proteolytic stability of antimicrobial peptides (AMPs), herein we developed a series of ultra-short triazine based amphipathic polymers (TZP) that are connected with ethylene diamine linkers instead of protease sensitive amide bond. The most potent oligomers, TZP3 and TZP5 not only displayed potent antibacterial action on various drug-resistant pathogens but also exhibited a strong synergic antibacterial activity in combination with chloramphenicol against multidrug-resistant *Pseudomonas aeruginosa* (MDRPA). Since most of atopic dermatitis (AD) infections are caused by bacterial colonization, we evaluated the potency of TZP3 and TZP5 on AD *in vitro* and *in vivo*. *In vitro* AD analysis of these two polymers showed significant inhibition against the release of *β*-hexosaminidase and tumor necrosis factor (TNF-*α*) from RBL-2H3 cells. In AD-like skin lesions in BALB/c mice model, these two polymers displayed significant potency in suppressing dermal and epidermal thickness, mast cell infiltration and pro-inflammatory cytokines expression. Moreover, these polymers exhibited remarkable efficacy over the allergies caused by the imbalance of Th1/Th2 by regulating total IgE and IgG2a. Finally, the impact of treatment effects of these polymers was examined through analyzing the weights and sizes of spleen and lymph node of AD-induced mice.

## Introduction

The rapid emergence of drug-resistant pathogens and the shortage in the discovery of new antibiotics become a major concern in the therapeutic world to treat infectious diseases that remain second leading killer globally causing high morbidity and mortality^1–4^. To overcome the drug resistance, antimicrobial peptides (AMPs), also called as host-defense peptides, are considered as an important class of antibiotics because of their rapid action on pathogens and their poor propensity to elicit drug resistance^5–7^. Even though many detailed studies and reports pertaining to the antimicrobial peptides have been reported, advancement into commercially available therapeutics is limited. The primary reason is protease instability, as it contains multiple amide bonds from 15 to 50 amino acid sequences. The other prominent reasons are expensive production cost associated with multistep synthesis, cytotoxicity to eukaryotic cells, and poor antimicrobial activity in the presence of physiological condition of salts^8–10^. To overcome the drawbacks mentioned above, several strategies have been implemented, including, modification in the peptide sequences using unnatural amino acids or *D*-amino acids and the development of new peptidomimetics to mimic the properties of AMPs^11–16^. Even though they show a substantial difference from peptides, the amide functionality remains unchanged, which is highly prone to protease destabilization. To overcome the above hurdles existing in the present methods and in a view of performing structure-activity relationship (SAR) study, it is imperative to design antibiotics with a simple design having two or three residues, avoiding amide linkage and with operational simplicity.

Taking the above facts into the considerations, we envisioned the design and synthesis of triazine based polymers consisting of lipophilic and hydrophobic residues as a new class of novel antibiotics. We preferred *s*-triazine for developing the new antibiotics because (i) synthesis of triazine based scaffolds holding hydrophobic and hydrophilic domains are highly facile, (ii) ethylene diamine was used as a linker that increases protease stability compare to the peptides with amide linkage, (iii) triazine monomer can be adopted to construct the amphipathic polymers through solid phase peptide synthesis (SPPS)^17^. Despite there are reports pertaining to the antibacterial activities of *s*-triazine derivatives^18–20^, to the best of our knowledge, amphipathic triazine polymer possessing both anti-bacterial and anti-atopic properties have not been reported yet. Thus, present work is a part of our research engaged on the development of new antibiotics^11,18,21,22^.

In the present work, we designed and synthesized triazine monomers holding hydrophilic and lipophilic moieties, which were adopted in SPPS for construction of a series of polymers ranging the length from two to five residues. These sequence defined polymers were screened against Gram-positive and Gram-negative bacterial strains. To identify the most potent compound for the therapeutic applications, we selected the dimer TZP-3 and trimer TZP-5 for further studies because of their enhanced antibacterial effects and low hemolytic activity. To evaluate the potential of TZP-3 and trimer TZP-5 against drug-resistant pathogens, we assayed against methicillin-resistant *S*. *aureus* (MRSA), vancomycin-resistant *E*. *faecium* (VREF) and multidrug-resistant *P*. *aeruginosa* (MDRPA). Further, these two polymers were investigated for their synergistic effect in combination with three clinically used antibiotics against multidrug-resistant *Pseudomonas aeruginosa* (MDRPA). As most of atopic dermatitis skin infections involve bacterial colonization, an antibacterial agent with enhanced anti-inflammatory action has been treated for AD^18^. In this context, TZP-3 and TZP-5 were evaluated for their potential on AD involving both *in vitro* and *in vivo*. Further, the impact of these two polymers on inhibiting both tumor necrosis factor (TNF-*α*) and *β*-hexosaminidase release from the RBL-2H3 cells were analyzed. Further, its underlying mechanism of action was studied. The histopathological changes such as IgG2a levels, serum IgE and mast cell infiltration were also investigated. In BALB/c mice model, cytokine expressions of AD-like skin lesions were investigated. Finally, weight and size reduction in spleen and lymph nodes of AD-induced mice after treating with TZP3 and TZP5 were analyzed.

## Results

### synthesis of amphipathic triazine polymers

**Figure 1:**
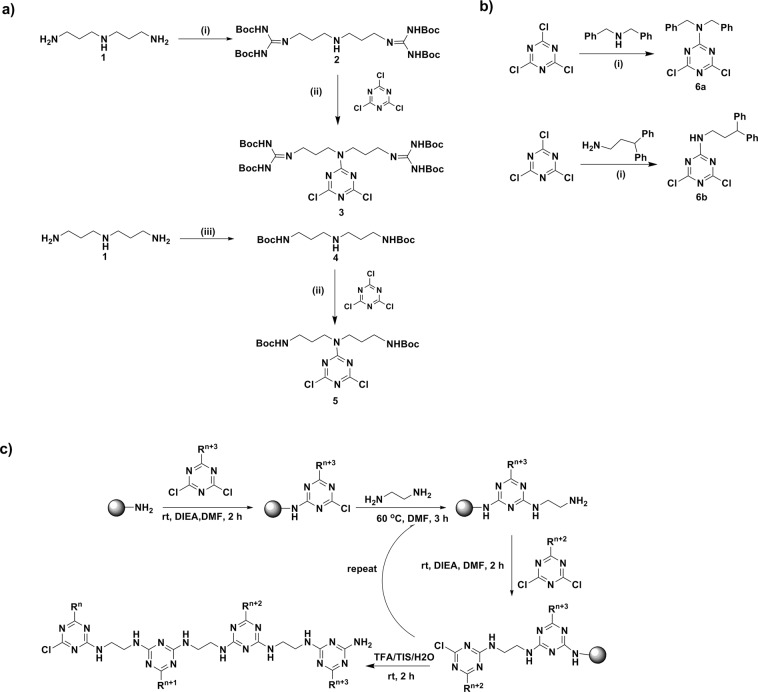
(**a**) Synthesis of hydrophilic monomers; reagents and conditions: (i) *N*,*N*’-di-boc-*N*”-trifylguanidine (**2**), TEA, DCM, rt, 6 h, 68%; (ii) DIEA, DCM, 0 °C, 3 h, 87–97%; (iii) Boc-ON, THF, 0 °C- rt, 20 h, 81%. (**b**) Synthesis of lipophilic monomers**;** reagents and conditions: (i) DIEA, DCM, 0 °C, 3 h, 54–67%; (**c**) Solid phase synthesis of a triazine based polymers.

It is known that the cyanuric chloride offers replaceable 2,4,6-positioned chlorine atoms that can be sequentially substituted using temperature gradient by various nucleophiles; thus it provides diversity in the structure by architecting the required substituents^23^. To derivatize a series of polymers for performing an intensive structure activity relationship study, we synthesized a variety of monomers holding lipophilic and hydrophilic residues. Hydrophilic monomers were prepared as delineated in Fig. 1a. For instance, the guanidinylation was carried out at the terminal amines of norspermidine (**1**) using *N*,*N*-di-boc-*N*-trifylguanidine, which resulted in the formation of **2**. Further, nucleophilic substitution reaction of **2** on cyanuric chloride in basic condition yielded the hydrophilic monomer **3** as an arginine mimic.

Another monomer **5** was initiated from the selective di-boc formation at the primary amines of norspermidine (**1**) in the presence of base at low temperature to yield **4**, which was upon treatment with cyanuric chloride in presence of *N*, *N*-diisopropylethylamine (DIEA) at ice-cold condition resulted in the formation of desired monomer **5** as a lysine mimic in good yield. Then the hydrophobic monomers were prepared as in Fig. 1b. Briefly, base mediated aromatic nucleophilic substitution reactions of amines including dibenzylamine and 3,3-diphenylpropan-1-amine over cyanuric chloride at low temperature resulted in the formation of lipophilic scaffolds **6a** and **6b**. After the successful synthesis of lipophilic and hydrophilic monomers, which were adopted for the sequence controlled amphipathic triazine polymer synthesis using Rink amide resin mediated conventional solid-phase peptide synthesis as shown in Fig. 1c. The resultant crude polymers were purified using preparative RP-HPLC. The molecular weight of synthesized polymers was determined by matrix-assisted laser-desorption ionization time-of-flight mass spectrometry (MALDI-TOF MS).

### Structure-antimicrobial activity study

**Figure 2:**
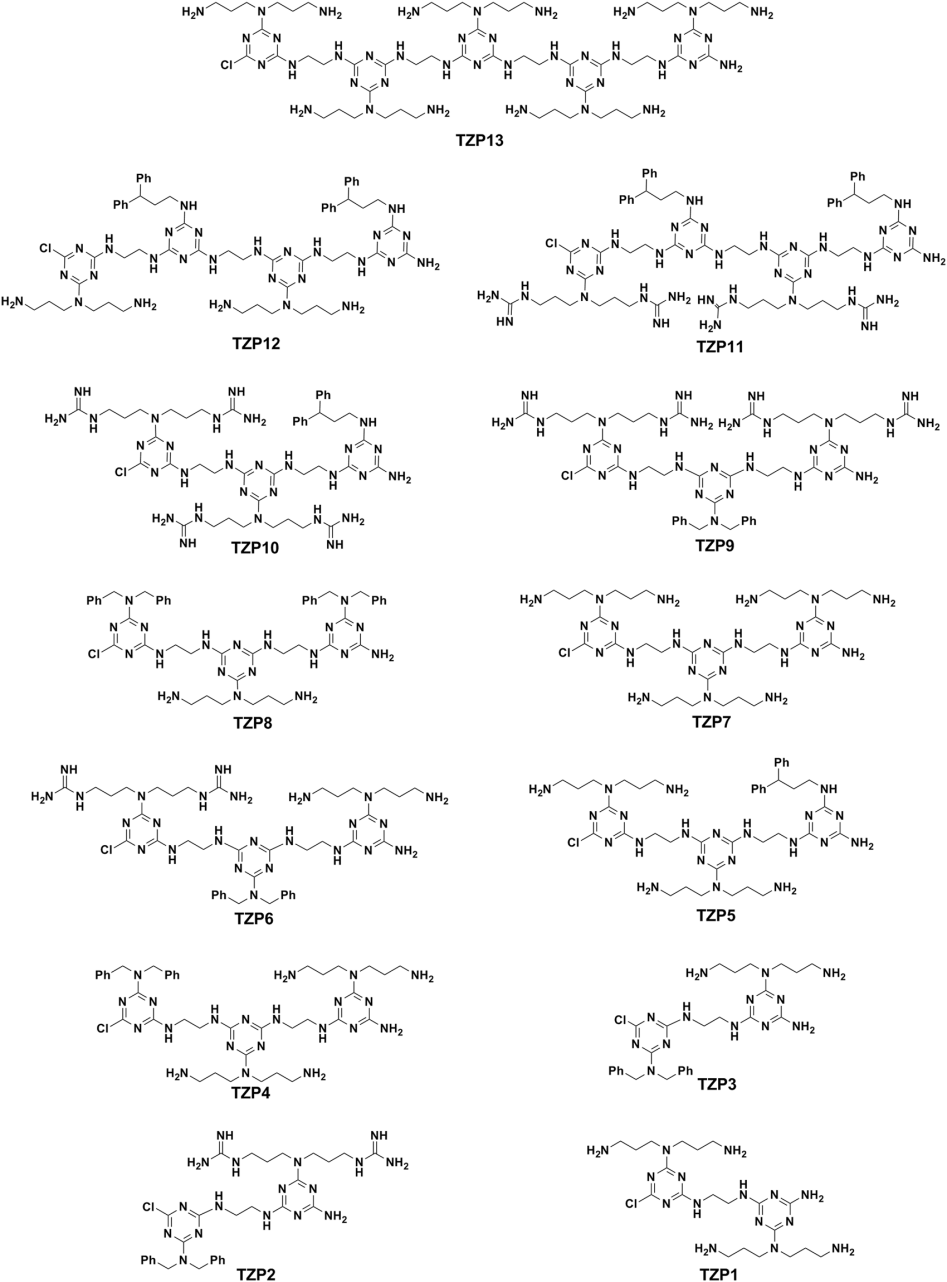
Structures of the designed antibacterial polymers.

Having synthesized the polymers, the antimicrobial activities of these polymers were evaluated against a panel of both Gram-negative bacteria and Gram-positive bacteria using the reference, melittin and the results are discussed based on the MICs (minimal inhibitory concentrations) in μg/mL. We began our structure activity study by synthesizing TZP13 that is a pentamer derived entirely from amine anchored hydrophilic triazine monomer for the possible comparison of the shortest antimicrobial peptide, WKWKW, assuming that triazine can act as tryptophan while propyl amine can mimic the lysine, as shown in Fig. 2. Surprisingly, this pentamer showed significant antibacterial activity against all the strains as melittin. Besides, it didn’t show any hemolysis at tested concentration (Table 1). This result implies that pentamer might have existed as an amphipathic structure by the possible array of hydrophobic triazine backbone and hydrophilic amines. Inspired by this encouraging result, we were curious to validate the impact of the length of polymers. Thus, we synthesized trimer, TZP7 by shortening two monomers from TZP13. Unfortunately, the MICs were not appreciable compared to TZP13. Further, we synthesized tetramers TZP11 and TZP12 consisting of two hydrophobic residues and two hydrophilic residues sequenced in an alternative fashion. Among them, the hydrophobicity was derived from two 3,3-diphenyl propyl groups in both tetramers, and for tuning the hydrophilicity, guanidine and amine anchored triazine monomers were used for the synthesis of TZP11 and TZP12, respectively. The antibacterial evaluation of these tetramers showed appreciable activity against all the strains, however, the hemolysis was very high even at very low concentration, which might be due to the enhanced hydrophobicity exerted from 3,3-diphenyl propyl residues and alkyl chains. Thus, we made TZP10 by removing a hydrophobic triazine unit from TZP11. As expected, TZP10 didn’t show any hemolysis at the tested concentrations. In addition, it showed an increased antibacterial effect against all the strains except for *P*. *aeruginosa*. Moving from guanidyl to amine in TZP10 resulted in TZP5 for finding the possible effect of charge.

**Table 1:** Antimicrobial and hemolytic activities and cell selectivity of the synthesized TZP polymers.

Comp.	MIC^a^ (μg/mL, [μM])				GM^b^ (μg/mL, [μM])	MHC^c^ (μg/mL, [μM])	TI (MHC/GM)
	Gram-negative bacteria		Gram-positive bacteria				
	*Escherichia coli* (KCTC 1682)	*Pseudomonas aeruginosa* (KCTC 1637)	*Staphylococcus aureus* (KCTC 1621)	*Bacillus subtilis* (KCTC 3068)			
TZP1	32 [60.8]	64 [121.7]	32 [60.8]	64 [121.7]	48.0 [91.3]	>256 [>486.7]	10.7
TZP2	2 [3]	8 [11.8]	2 [3]	2 [3]	3.5 [5.2]	245 [362.2]	70.0
TZP3	4 [6.8]	8 [13.5]	4 [6.8]	2 [3.4]	4.5 [7.6]	>256 [>432.4]	113.8
TZP4	2 [2.3]	4 [4.7]	2 [2.3]	2 [2.3]	2.5 [2.9]	>256 [>298.7]	204.8
TZP5	4 [3]	4 [3]	4 [3]	2 [1.5]	3.0 [2.3]	>256 [>193.2]	170.7
TZP6	2 [2.1]	4 [4.2]	2 [2.1]	2 [2.1]	2.5 [2.7]	>256 [>271.8]	204.8
TZP7	16 [20.2]	8 [10.1]	16 [20.2]	8 [10.1]	12.0 [15.2]	>256 [323.2]	42.7
TZP8	8 [8.7]	>64 [>69.3]	8 [8.7]	8 [8.7]	38.0 [41.1]	26 [28.1]	0.7
TZP9	2 [1.9]	8 [7.8]	2 [1.9]	2 [1.9]	3.5 [3.4]	>256 [>249.5]	146.3
TZP10	4 [3.8]	16 [15.4]	4 [3.8]	4 [3.8]	7.0 [6.7]	>256 [>246.2]	73.1
TZP11	8 [9.2]	16 [18.3]	8 [9.2]	8 [9.2]	10.0 [8.7]	16 [18.3]	1.6
TZP12	4 [2.9]	16 [11.5]	4 [2.9]	8 [5.8]	8.0 [5.8]	26 [18.7]	3.3
TZP13	8 [6.6]	8 [6.6]	8 [6.6]	4 [3.3]	7.0 [5.7]	>256 [>210.2]	73.1
melittin	4 [1.4]	16 [5.6]	4 [1.4]	8 [2.8]	8.0 [2.8]	8.5 [3]	1.1

^a^MICs (minimal inhibitory concentrations) were determined as the lowest concentration of the polymer that causes 100% inhibition of microbial growth.

^b^GM denotes the geometric mean of MIC values from selected six bacterial strains.

^c^MHC is the lowest polymer concentration that produces 10% hemolysis of sheep red blood cells.

Surprisingly, TZP5 displayed 2 and 4 folds of increased activity against *B*. *subtilis* and *P*. *aeruginosa*, respectively, without showing hemolytic activity at tested concentration. Next, to validate the effect of hydrophobic group in TZP10, we have replaced 3,3-diphenyl propyl anchored triazine residue with dibenzyl anchored triazine monomer, resulting in the trimer TZP9. The antibacterial efficacies of TZP9 were increased by two folds against all the tested bacterial strains compared to that of TZP10, without showing any hemolysis at tested concentrations.

This increased antibacterial effects can be attributed to the fact that the hydrophobicity derived from dibenzyl group can balance the charges from four guanidyl residues effectively, rather than 3,3-diphenyl benzyl group. Next, we synthesized trimers, TZP4 and TZP6 by varying the hydrophilic monomers of TZP9. TZP4 was constructed in a sequence that two units of amine anchored triazine monomers and a dibenzyl anchored triazine monomer were connected by ethylene diamine linkers. Trimer, TZP6 was constructed from each amine, dibenzyl, and guanidyl anchored triazine monomers. These two trimers displayed almost the same antibacterial effects as TZP9 without showing any hemolysis at tested concentrations. It is inferred from the results of TZP4 and TZP6 that varying amine or guanidine didn’t make any significant difference in the antibacterial effect. Thus, amine and guanidine can be used for designing antibacterial agents involving triazine polymers. Further, it was proven by the fact that increasing the hydrophobicity in trimer TZP8 ultimately loosed its antibacterial effects from 4–16 folds and showed elevated hemolysis even at lower concentrations. To find out the minimum polymer length that requires for displaying significant antibacterial activity, we synthesized three dimers with a different combination of hydrophilic and hydrophobic monomers. Among them, TZP1, is the dimer with the combination of two amine based hydrophilic residues, loosed its activity against both Gram-positive and Gram-negative strains. This suggested that TZP1 required hydrophobicity to balance the charge derived from the amines. As dibenzyl monomer displayed enhanced antibacterial effects in TZP4, TZP6 and TZP9, we envisaged the construction of TZP2 and TZP3 with dibenzyl monomer and a hydrophilic monomer. Thus, we constructed TZP2 with guanidyl and dibenzyl anchored triazine monomers. Similarly, TZP3 was made up of amine and dibenzyl anchored triazine monomers. Interestingly, these two short polymers displayed significant antibacterial activity against all the bacterial strains, which were better than that of melittin and almost as potent as TZP4. However, TZP3 was found to be more efficient than TZP2 because it didn’t show any hemolysis at tested concentrations, whereas TZP2 showed hemolysis at tested concentration. Thus, we identified TZP3 as one of the most potent dimers through our antibacterial screening.

### Cell selectivity

**Figure 3:**
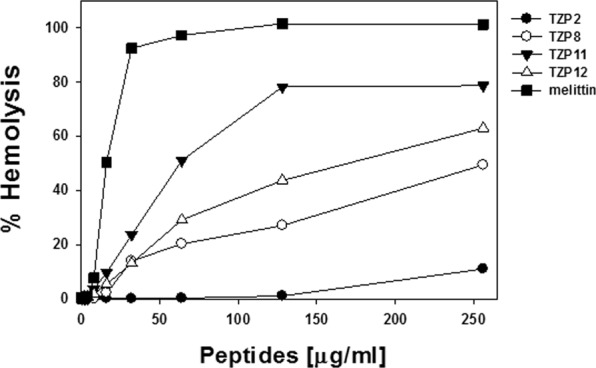
Concentration-response curves of percent hemolysis of the designed antimicrobial polymers against sheep red blood cells (s-RBCs). Other TZP polymers did not cause any hemolysis even at 256 μg/mL.

To assess the bacterial selectivity of the newly synthesized TZP polymers, all the compounds were evaluated for their hemolytic activity against sheep red blood cells (sRBCs) in the range of 0~256 μg/mL as shown in Fig. 3. TZP11 and TZP12 displayed maximum hemolysis at 125 μg/mL and 260 μg/mL, respectively, while most of the compounds failed to show appreciable hemolysis. These results suggest that these compounds possess a potential for use as an antimicrobial therapeutic.

### Protease stability

**Figure 4:**
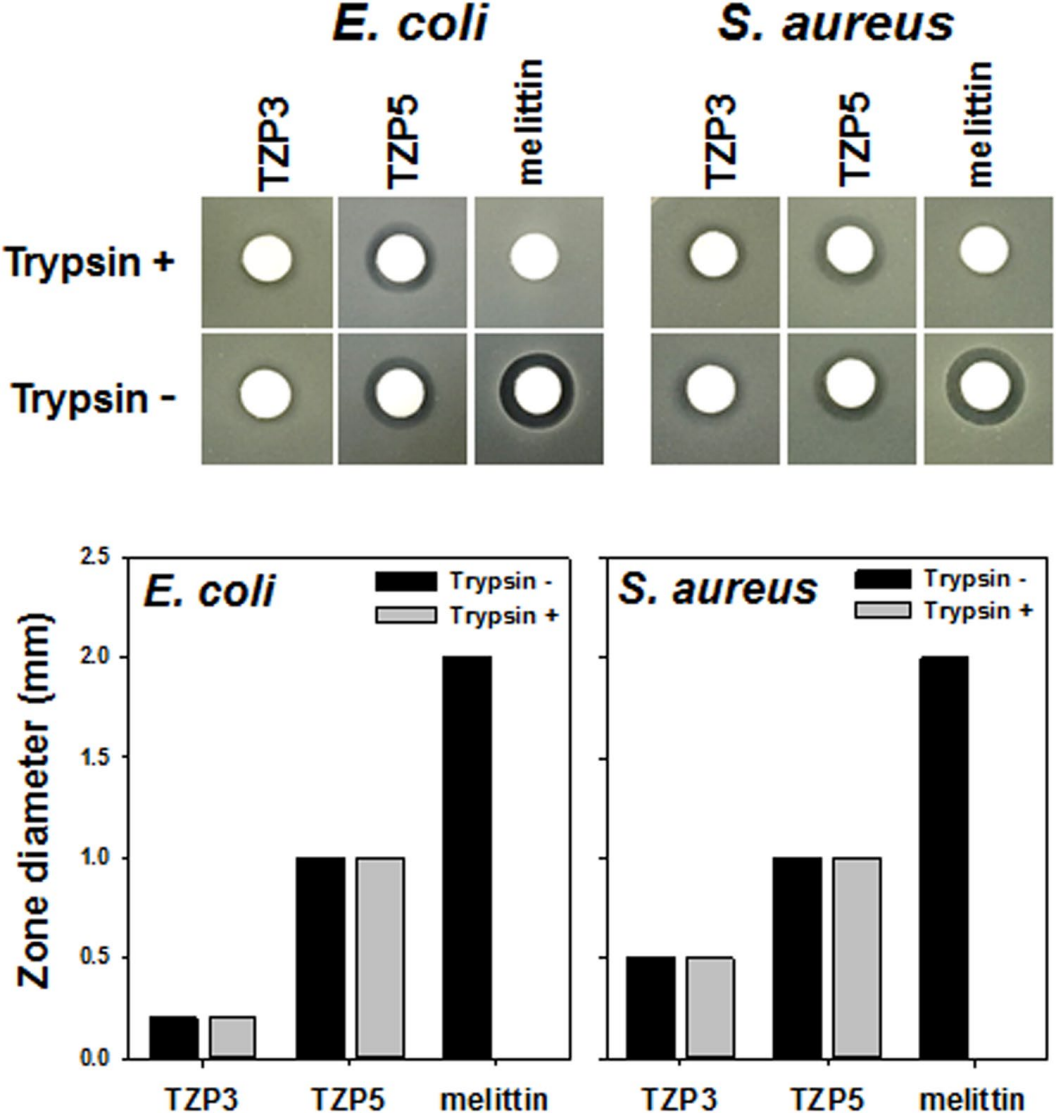
Inhibition of antimicrobial activity of TZP3, TZP5 and melittin by tryptic digestion.

Proteolytic instability of peptides in the presence of enzymes remains one of the major challenges in the advancement of peptide based antibacterial as therapeutic agents. As lysine containing peptides are highly prone to trypsin-mediated degradation, we investigated the proteolytic stability of TZP3 and TZP5, which contain alkyl amines as a mimics of lysine, as shown in Fig. 4. Although TZP3 and TZP5 were incubated with trypsin for 24 h, these compounds did not lose any of their antibacterial effects against both *E*.*coli* and *S*.*aureus*. At the same time, melittin was found to lose its antibacterial activity entirely due to the proteolytic degradation by trypsin.

### Antibacterial activity of TZP-3 and TZP-5 against antibiotic-resistant bacteria

In mid of escalating crisis in the invention of new antibiotics, infections caused by multidrug-resistant bacteria are considered as an enormous and growing threat to the people health. For instance, hospital-acquired infections are mainly due to the multidrug-resistant bacteria, including methicillin-resistant *S*. *aureus* (MRSA), vancomycin-resistant *E*. *faecium* (VREF), and Gram-negative bacteria multidrug-resistant *P*. *aeruginosa* (MDRPA). Thus, the discovery of new antibiotics with great potency toward drug-resistant bacteria remains as an essential need in modern health care^24^. Therefore, TZP3 and TZP5 screened for their antibacterial activity against three MRSA strains (CCARM 3089, CCARM 3090, and CCARM 3095), two MDRPA strains (CCARM 2095 and CCARM 2109) and a VREF strain (ATCC 51559). Interestingly, both the compounds TZP3 and TZP5 revealed significant activities against all the strains compared to that of melittin (Table 2). TZP5 showed superior potency against MRSA and VREF bacterial strains compare to that of reference, melittin. In particular, it showed four folds of superior potency against CCARM 3095 (MRSA) strains compared to that of melittin, and in the case of MDRPA strains, it showed the activity profiles as equal as the melittin. TZP3 showed similar potency as melittin against all the tested drug-resistant strains, except for CCARM 3095, where it showed a two-fold better profile of activity compared to melittin. These results suggest that our tested compounds TZP3 and TZP5 could be used as a representative for designing antibiotics that are effective against drug-resistant bacteria.

**Table 2:** Antimicrobial activities of TZP3 and TZP5 against antibiotic-resistant bacterial strains.

Microorganisms	MIC (µg/mL) [μM]		
	TZP3	TZP5	melittin
**Methicillin-resistant** ***Staphylococcus aureus*** **(MRSA)**			
CCARM 3089	8 [13.5]	4 [3]	8 [2.8]
CCARM 3090	8 [13.5]	4 [3]	8 [2.8]
CCARM 3095	8 [13.5]	4 [3]	16 [5.6]
**Multidrug-resistant** ***Pseudomonas aeruginosa*** **(MDRPA)**			
CCARM 2095	8 [13.5]	8 [6]	8 [2.8]
CCARM 2109	8 [13.5]	8 [6]	8 [2.8]
**Vancomycin-resistant** ***Enterococcus faecium*** **(VREF)**			
ATCC 51559	8 [13.5]	4 [3]	[2.8]

### Synergy effects of TZP3 and TZP5 with clinically used antibiotics

The emerging multi-drug resistant bacterial infections have serious implication for global public health. We investigated the synergistic effect of TZP3 and TZP5 in combination with three clinically used antibiotics including, chloramphenicol, ciprofloxacin, and oxacillin that are showing the different mechanism of antimicrobial action against *Pseudomonas aeruginosa* (MDRPA) and also display the resistance with a MIC range of 512−1024 μM. The interactions due to the combinations of drugs can exist in three different forms, namely synergism, additivity, and antagonism, which represents the effect of two drugs combined is stronger, equal, and weaker than that of the equal doses of individual drugs, respectively. The fractional inhibitory concentration index (FICI) data of the combinations of antibiotics with two triazine polymers are given in Table 3. Interestingly, both TZP3 and TZP5 displayed a strong synergy activity (FICI 0.281) in combination with chloramphenicol against MDRPA. In combination with ciprofloxacin, TZP3 (FICI 0.75) and TZP5 (FICI 0.5) showed an additive and synergy effects, respectively. However, these two molecules exhibited an indifferent effect (FICI 2.0) in combination with oxacillin. These results suggested that TZP3 and TZP5 in combination with chloramphenicol are potential antibiotic adjuvants against MDRPA infection.

**Table 3:** The synergy between TZP3 or TZP5 and clinically used antibiotics against. Multidrug-resistant *Pseudomonas aeruginosa (MDRPA)* (CCARM 2095).

MIC_A_	[A]	FIC_A_^a^	MIC_B_	[B]	FIC_B_	FICI^b^	Interaction
Chloramphenicol			TZP3				
512	16	0.031	16	4	0.25	0.281	synergy
Chloramphenicol			TZP5				
512	16	0.031	16	4	0.25	0.281	synergy
Ciprofloxacin			TZP3				
1024	256	0.25	16	8	0.5	0.75	additive
Ciprofloxacin			TZP5				
1024	256	0.25	16	4	0.25	0.5	synergy
Oxacillin			TZP3				
1024	1024	1.0	16	16	1.0	2.0	indifferent
Oxacillin			TZP5				
1024	1024	1.0	16	16	1.0	2.0	indifferent

^a^FIC: Fractional inhibitory concentration.

^b^FICI: Fractional inhibitory concentration index.

### Atopic dermatitis

AD, can be classified as a complex inflammatory skin disease that particularly affects the young children, and is characterized by Th2 cells immune response and sensitization of immunoglobulin (Ig)E^25^. The pathogenesis of AD arising from various factors, including complex interaction of genetic factors with skin barrier dysfunction, and immunologic and environmental factors^26^. In general, clinical features of AD involve eczematous skin lesions, scaling, and pruritus^27^. Generally, the pathophysiology of the AD associated with disruption of epidermal structure, and infiltration of mast cells, T-helper cells, and neutrophils into the dermis region^28^. In AD patients, skin infections are mainly caused by bacterial colonization, for instance, *S*. *aureus* is instrumental in most of the skin infections in AD. Moreover, drug resistant pathogens such as MRSA produces a large number super antigens that increases the severity of infections and cutaneous inflammation in AD patients^18,29,30^. Thus, considering the proficient anti-bacterial activity of our synthesized polymers, we speculated that they could be the right choice for treating AD.

### Effects of TZP3 and TZP5 on AD-like skin lesion in a BALB/c mouse model and mast cell infiltration

**Figure 5:**
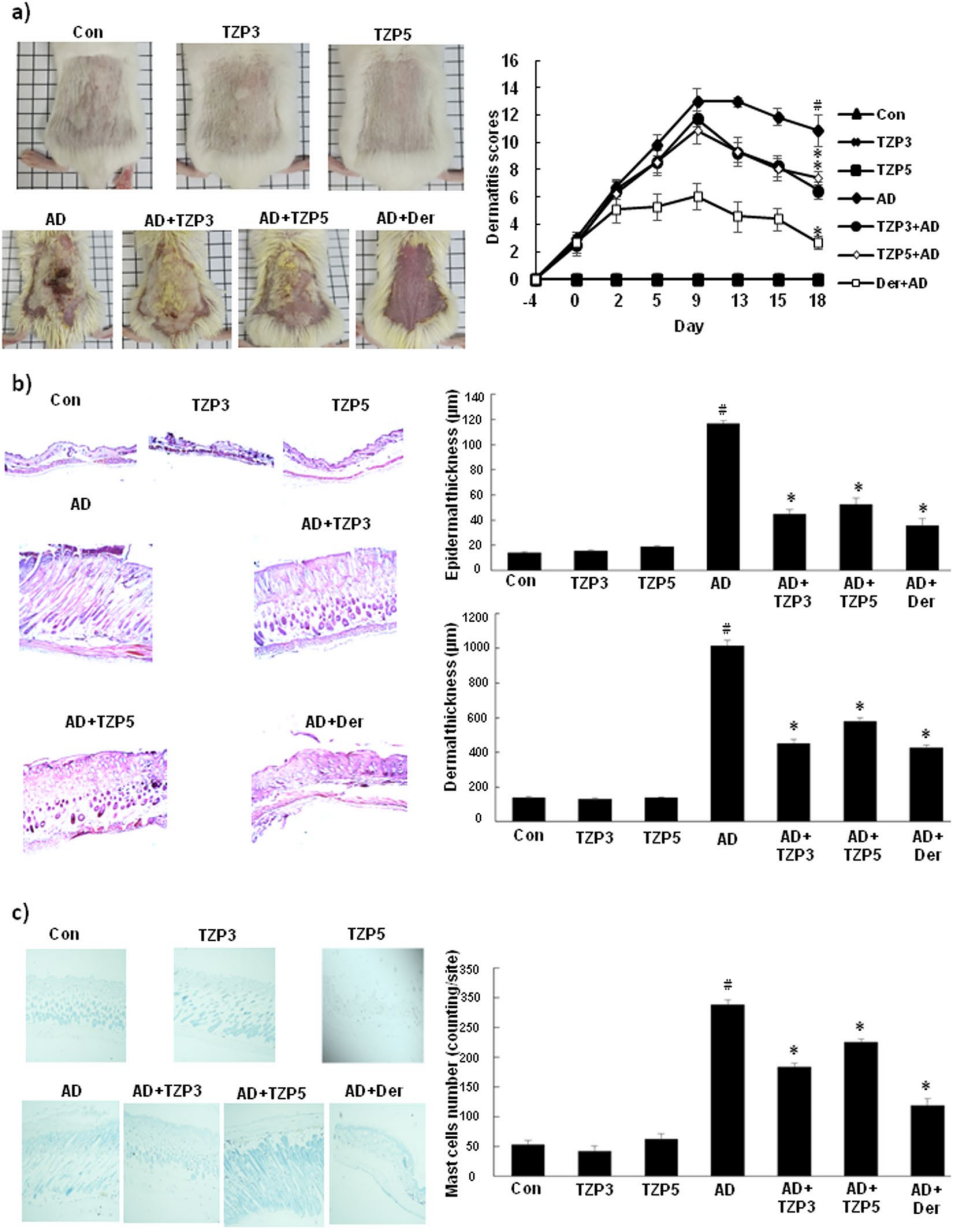
(**a**) AD induction and treatment in the dorsal skin of mice models and corresponding dermatitis score; (**b**) Hematoxylin and eosin-stained microphotographs and measurement of the epidermal and dermal thickness; (**c**) The number of infiltrated mast cells was determined by toluidine blue staining. CON represents control; Der represents the AD drug, dermatop; Significant differences at ^#^*p* < 0.05 compared with the Con group. Significant differences at ^*^p < 0.05 com*p*ared with the AD group.

TZP3 and TZP5 were probed for their potential on AD-like skin lesion in a BALB/c mouse model that was developed by 2,4-dinitrochlorobenzene (DNCB) treatment. The repetitive and periodical application of DNCB induced swelling with a significant increase in the thickness on the dorsal skin surface of BALB/c mice as shown in Fig. 5a. However, treatment of TZP3 and TZP5 daily for 18 days exhibited a remarkable effect on AD. In addition, to assess the potential of the inhibitors, dermatitis score was calculated by evaluating the skin features of dermatitis including, i. erythema and hemorrhage, ii. pruritus and dry skin, iii. edema and excoriation, iv. erosion, and v. lichenification. Even though, the dermatitis scores of AD developed mice were significantly high and progressive, after the treatment of TZP3 and TZP5, AD mice showed significantly decreased score and followed a similar pattern as that of AD drug, dermatop. Interestingly, after day-9, the dermatitis scores of TZP3 and TZP5 were significantly reversed compared to the AD-induced mice.

To investigate the effect of TZP3 and TZP5 on skin hypertrophy and granulocyte infiltration, microscopic images of DNCB-induced epidermal and dermal thickness were examined after treating with TZP3 and TZP5 as shown in Fig. 5b. The repeated DNCB application induced pathological changes, including thickening of the epidermis and dermis of AD mice. However, treatment with TZP3 and TZP5 showed a significant decrease (more than 50%) in both epidermal and dermal thickness. In particular, TZP3 showed better efficiency than that of TZP5, which was almost as good as the positive control, dermatop.

The effects of TZP3 and TZP5 on mast cell infiltration were investigated as shown in Fig. 5c. Interestingly, topical application of TZP3 and TZP5 significantly suppressed the infiltration of mast cells in the skin lesions of the AD mice. In particular, TZP3 showed significant effects compare to TZP5. Thus, these two compounds can be considered as a possible model for designing drugs for IgE mediated allergies, controlling through mast cells infiltration.

### Effects of TZP3 and TZP5 on various inflammatory cytokine expressions

**Figure 6:**
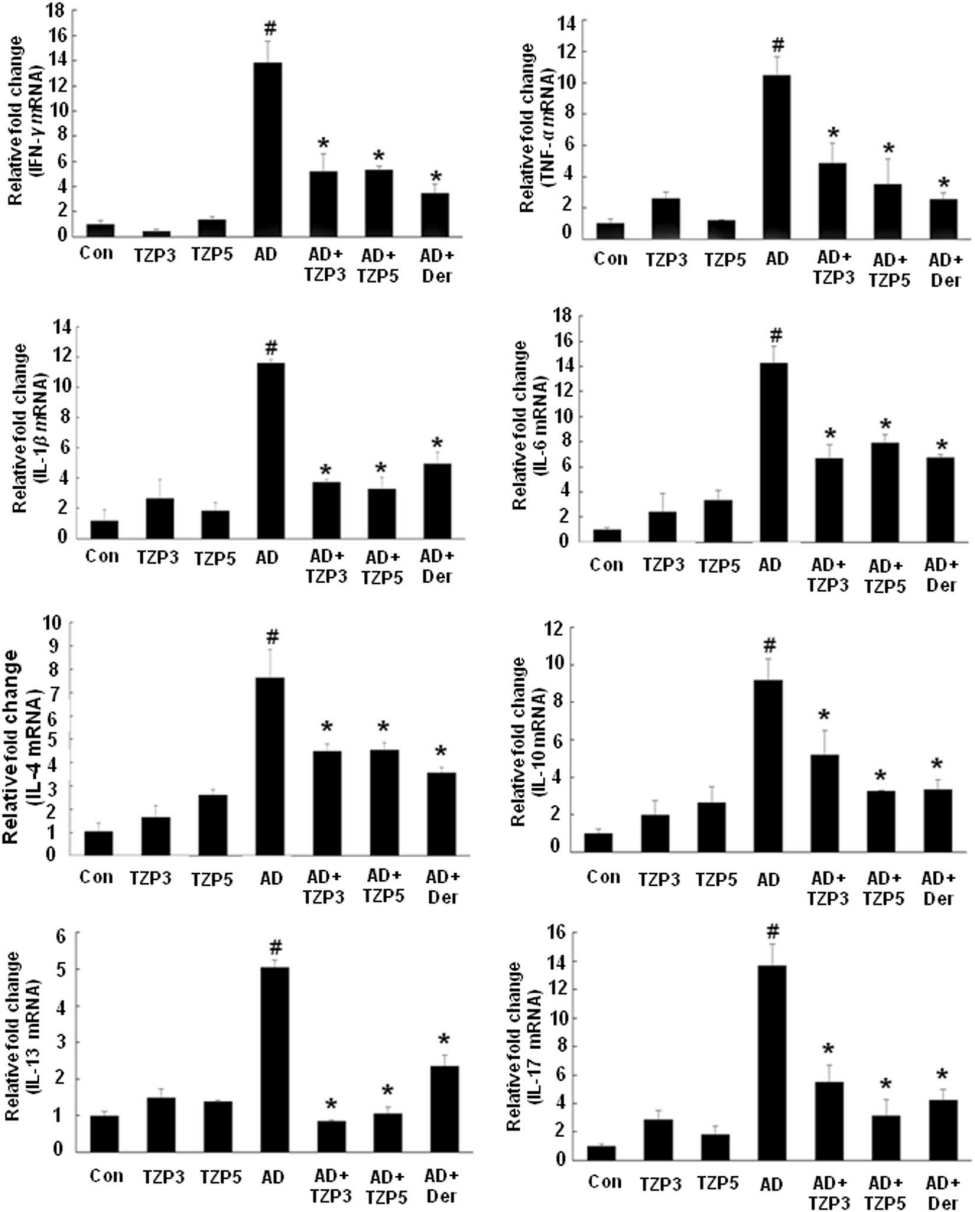
Effects of TZP3 and TZP5 on the expression of various pathogenic factors in the dorsal tissue. Data are presented as mean ± SD of triplicate determinations of each group (n = 8). CON represents control; Der represents the AD drug, dermatop; Significant differences at ^#^*p* < 0.05 compared with the Con group. Significant differences at *p < 0.05 com*p*ared with the AD group.

We investigated the impact of TZP3 and TZP5 on inflammatory cytokine expressions in AD-like skin lesions using real-time PCR. The expressions of IFN-*γ*, TNF-*α*, IL-1*β*, IL-6, IL-4, IL-10, IL-13 and IL-17 were increased by repeated oral application of DNCB (Fig. 6). Treatment of TZP3 and TZP5 showed significant effects in reducing the upregulated expression of cytokines in AD-like skin lesions compared to the control drug, dermatop. In the case of IFN-*γ*, even though the inhibitory effects showed by both compounds were little weaker than the control, dermatop; still, they displayed the inhibitory potential around 60%. Similarly, TZP3 and TZP5 showed TNF-*α* inhibition of approximately 55% and 65%, respectively. It is pertinent to note that in the cases of IL-1*β*, IL-13, the inhibitory potentials shown by TZP3 and TZP5 were approximately more than 70% and 75%, respectively, which were more potent than the control, dermatop. However, they displayed IL-4 suppression approximately, 35%. In the case of IL-6, TZP3 and TZP5 suppressed the expression by approximately 60% and 50%, respectively. Surprisingly, in IL-10, TZP5 showed almost similar potency with the dermatop and it was better than that of TZP3 that showed around 35% suppression. Similarly, in IL-17, TZP5 displayed more than 85% potency in inhibiting the expressions, which was better than that of TZP3 as well as control. Meanwhile, the TZP3 displayed 60% inhibition. These results collectively infer that TZP3 and TZP5 were effective in suppressing the mRNA expressions of Th1, Th2 and Th17 cytokines in both acute and chronic inflammation in AD-lesions.

### Effects of TZP3 and TZP5 on protein expressions

**Figure 7:**
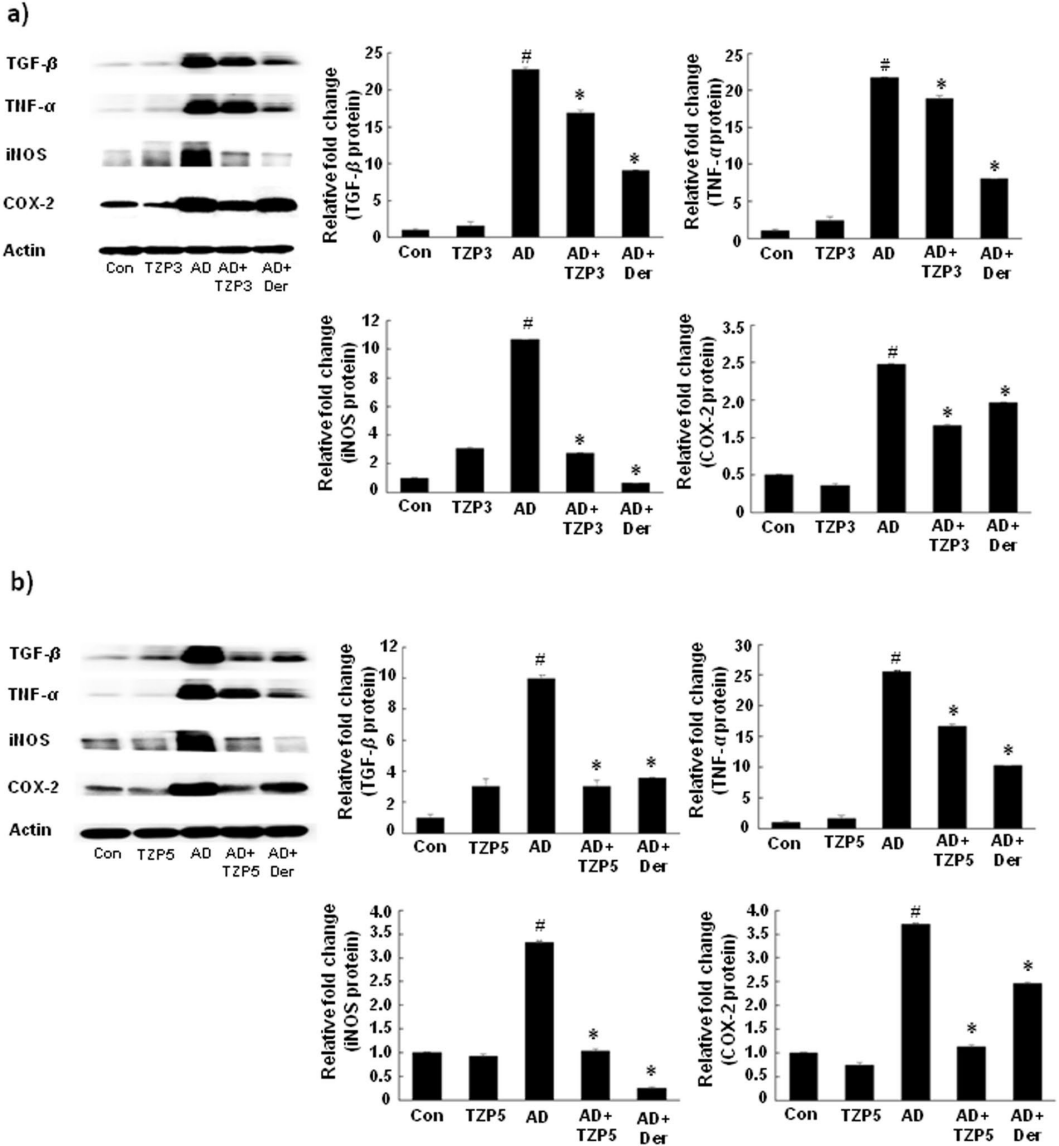
Effects of TZP3 (**a**) and TZP5 (**b**) on inflammatory mediators in dorsal tissue. CON represents control; Der represents the AD drug, dermatop; Significant differences at ^#^*p* < 0.05 compared with the Con group. Significant differences at ^*^p < 0.05 com*p*ared with the AD group. (*Full-length blots are included in Supplementary information*).

As shown in Fig. 7, in all the cases of AD, a high level of protein expression was observed. However, treatment of TZP3 and TZP5 showed a significant decrease in all protein expressions. In particular, TZP3 and TZP5 displayed remarkable effects in suppressing COX-2 expression level, which was better than the AD drug, dermatop. Similarly, TZP5 also showed remarkable potency in suppressing TGF-*β* than the positive control dermatop.

### Treatment of TZP3 and TZP5 decreases serum IgE and IgG2a levels in AD mice

**Figure 8:**
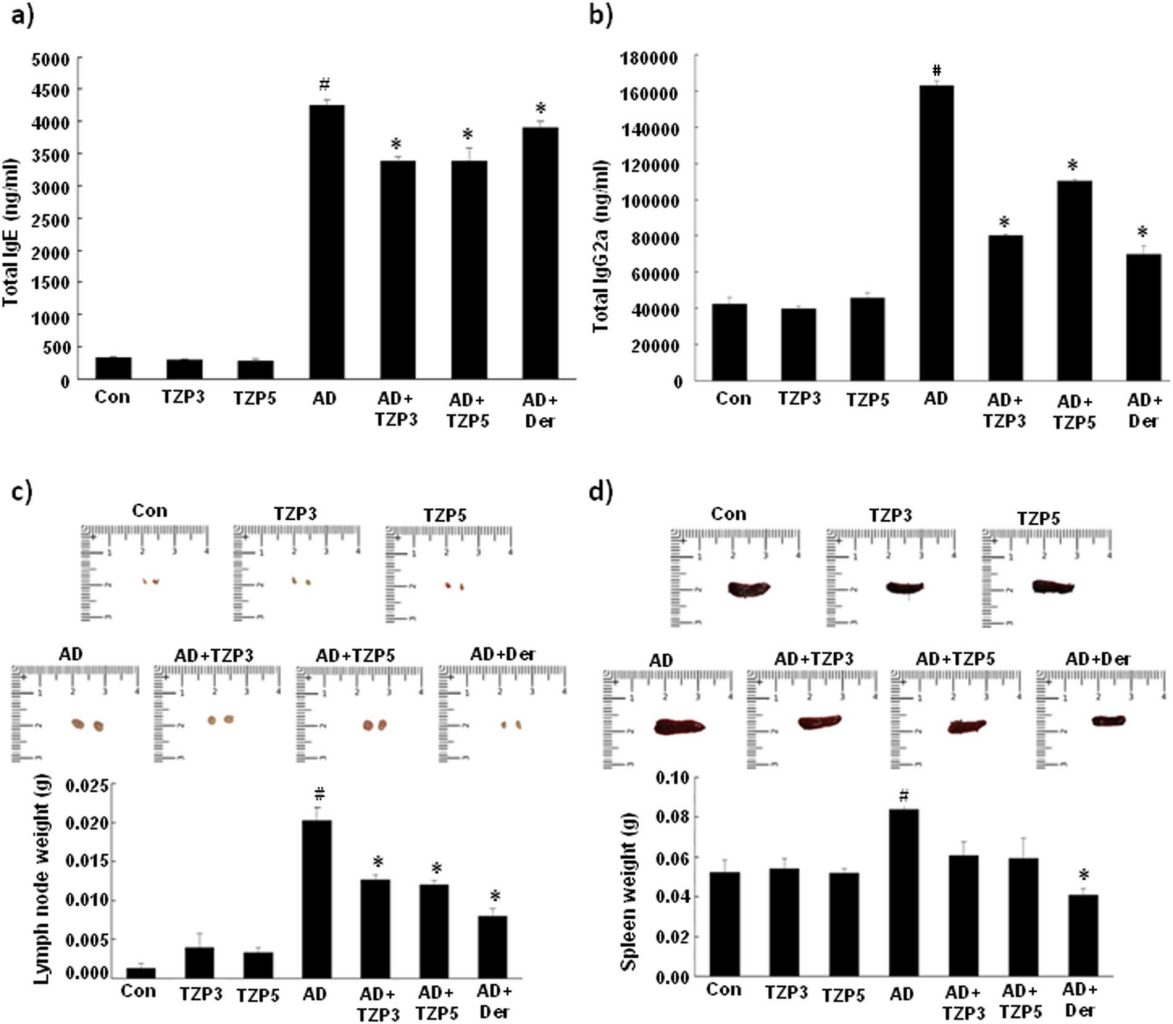
Effect of TZP3 and TZP5 on serum immunoglobulin levels. Plasma levels of total IgE (**a**) and total IgG2a (**b**). Photographs represent the size and weight of lymph nodes (**c**), and spleens (**d**) of DNCB induced AD mice. The millimeter ruler images represent variation in the size of lymph nodes (**c**) and spleens (**d**); the bar diagram describes the variation in the weight of lymph nodes and spleens. CON represents control; Der represents the AD drug, dermatop; Significant differences at ^#^*p* < 0.05 compared with the Con group. Significant differences at *p < 0.05 com*p*ared with the AD group.

To identify whether TZP3 and TZP5 possibly exert their effects through a Th1 or Th2 response, serum levels of total IgE and total IgG2a were measured. Repeated application of DNCB caused an apparent elevation of total IgE and IgG2a (Fig. 8a,b). However, TZP3 and TZP5 treated mice showed significantly reduced serum levels of both total IgE and total IgG2a than the AD-induced mice. In the case of total IgE, TZP3 and TZP5 showed better effect than the positive control, dermatop treated mice, and in the case of total IgG2a, TZP3 showed almost equal potency as compared to that of positive control and showed better effect than that of TZP5. Thus, it is understood from the suppression of serum levels of total IgE and IgG2a that TZP3 and TZP5 showed their effects possibly by inhibiting both Th2 and Th1 cellular responses.

### Effect of TZP3 and TZP5 on lymph node and spleen

As shown in Fig. 8c, we observed that the weight and size of the lymph node were remarkably increased in DNCB treated AD induced mice. Treatment of TZP3 and TZP5 on AD induced mice showed more than 35% decrease in both weight and size of lymph node compared to that of untreated AD-mice. Moreover, investigation of spleen’s weight and size was carried out to assess the health and immunological status of AD-induced mice. As shown in Fig. 8d, DNCB treated AD induced mice showed an increase in spleen weight and size compared to the drug-treated and vehicle-treated mice. However, oral administration of TZP3 and TZP5 on AD induced mice showed a significant decrease in both weight (*p* < 0.05) and the size of spleen compared to that of AD-induced mice.

## Discussion

Through systematic structure activity relationship study, it is inferred that (i) amphipathic triazine polymers with potent antimicrobial activity can be achieved from monomers containing hydrophilic and hydrophobic residue; (ii) In the possible study of mimicking shortest antimicrobial peptide, WKWKW, triazine scaffold can play a hydrophobic role like tryptophan, (iii) we achieved potent antimicrobial agent made up of only two triazine residues, which was more potent than melittin, (iv) we are successful in attaining triazine based antibacterial polymers through simple and convenient SPPS using ethylenediamine linkers as an alternative to amide bonds, which solved the proteas instability issue to the antimicrobial agents.

Further, hemolysis study also demonstrates that most of the polymers didn’t show any hemolysis even at higher concentration, which proves their selective mode action towards the bacteria. In particular, dimer TZP-3 and trimer TZP-5 showed enhanced antibacterial action against both Gram-positive and Gram-negative bacterial strains without showing any hemolysis even at elevated concentrations. Considering the emerging threat of multidrug-resistant pathogens on the global health care, evaluation of TZP-3 and TZP-5 against antibiotic-resistant bacteria suggest that these oligomers poses significant activity against various MRSA, MDRPA and VREF bacterial strains. Proteas stability is a major concern that impedes the advancement of the peptides into corresponding drugs. However, TZP3 and TZP5 didn’t lose any antibacterial effect in trypsin-mediated degradation study, proving their proteolytic stability. Combination therapy of antimicrobial agents is often proposed as a solution to combat MDR isolates and to decrease the probability of emerging resistance^31^. Thus, several studies have reported synergistic effects of combinations of AMPs with clinically used antibiotics^32^. However, triazine polymers based combination studies are not yet reported. Our study suggest that TZP3 and TZP5 are efficient in enhancing antibacterial action by showing synergy effects in combination with clinically used antibiotics, chloramphenicol.

AD is a complex and chronic inflammatory skin disease. It is established that bacterial infections in AD enhance the allergic responses including total IgE, and skin Th2 cytokine expressions. Thus, understanding the mechanisms of which may lead to potential therapeutic interventions^30^. Considering the proficient anti-bacterial effects of TZP3 and TZP5, we investigated their potential on an atopic dermatitis mice model. Initially, TZP3 and TZP5 were evaluated for their cytotoxicity in HaCaT cells. The MTT assay results suggested that these polymers didn’t show significant cytotoxicity at the maximum tested concentration (20 µg) (see Supplementary Fig. S3). During the allergy, mast cells produce proinflammatory mediators such as cytokines and chemokines, upon activation with antigens. TNF-α, is one of the well-known proinflammatory cytokines, inhibition of which is considered as a worth strategy of preventing the allergy^33^. TZP3 and TZP5 exert anti-allergic effects by inhibiting the TNF-*α* induced from mast cells (Fig. S1). *β*-hexosaminidase is considered as a degranulation marker of mast cells, which contain allergic mediators^34^. Usually, histamine and *β*-hexosaminidase are released together during mast cells activation, which is induced by an antigen or degranulation inducer. Dose-dependent inhibition of TZP3 and TZP5 against *β*-hexosaminidase suggest that they can act as an anti-allergic inhibitor by suppressing the degranulation in mast cells (Fig. S2). Investigation of TZP3 and TZP5 on AD-like skin lesion in a BALB/c mouse model revealed that these polymers not only have significant potential on reducing the thickness on the dorsal skin surface but also on reducing the thickness of epidermal and dermal thickness, proving their impact on skin hypertrophy and granulocyte infiltration. Usually, in the IgE mediated allergies including AD, elevated levels of mast cells are predominant. Upon activation of mast cells, histamine is released as one of the immune mediators; thus, elevated levels of histamine have been observed in AD patients. The suppression effect on mast cell infiltration exerted by TZP3 and TZP5 suggest that they can be considered as a possible model for designing drugs for IgE mediated allergies.

It is reported that, over expressions of Th2 cytokines including IL-4, IL-10 and IL-13 are predominant in the acute phase of AD lesions, which stimulate the high production of IgE^35^. IL-17 is also involved in most of the skin inflammation as a proinflammatory cytokine. Thus, an increased number of Th17 can be found in peripheral blood and acute lesional skin of AD^36^. Meanwhile, Th1 producing IFN-γ and other proinflammatory cytokines such as TNF-*α* and IL-6 were abundant in the chronic phase of AD^37,38^. Treatment of TZP3 and TZP5 showed significant potency in suppressing the *m*RNA expressions of Th1, Th2 and Th17 cytokines in both acute and chronic inflammation in AD-lesions. COX-2, is one of the enzyme isoforms of COX, plays prominent roles in allergic reactions and mast cell-mediated inflammations^39,40^. As inflammatory cytokines and endotoxins induce the COX-2 enzymes, alleviating upregulated COX-2 and inflammatory mediators expressions can be considered as an attractive strategy for the development of anti-allergic drugs^33^. TZP3 and TZP5 showed considerable effects in suppressing the protein expression level, including TGF-*β*, TNF-*α*, iNOS, and COX-2. Thus, TZP3 and TZP5 can be an anti-allergic drug model by deterring mast cell-dependent inflammatory mediators.

In general, AD is directly associated with the imbalance of Th1/Th2 responses. Moreover, patients affected with AD usually observed with elevated levels of total IgE and IgG2a as a response to the environmental allergens. The hyperproduction of IgE and IgG2a is associated with cellular responses of Th2 and Th1, respectively^41^. TZP3 and TZP5 displayed significant suppression of serum levels of total IgE and IgG2a, possibly by inhibiting both Th2 and Th1 cellular responses. Lymph node tends to increase its size during the stress that arises due to the inflammatory mediators. Similarly, as the spleen is also sensitive to inflammatory mediators and it tends to increase its weight and size during the AD. However, TZP3 and TZP5 treatment displayed a remarkable decrease in both the size and weight of lymph node and spleen in AD affected mice. Collectively, these results suggest that suppression of serum total IgE and Th2 cytokine expression by TZP3 and TZP5 possibly caused by affecting the bacterial infections in AD, thus our polymers opened a new pathway as an anti-atopic drug model with enhanced antibacterial effects.

## Conclusion

As compared with their small molecular counterparts, antimicrobial polymers demonstrate superior efficacy^42^, we designed and synthesized a series of *s-*triazine-based amphipathic antibacterial polymers. Against drug-resistant pathogens, the most potent polymers TZP3 and TZP5 showed significant antibacterial effects while showing significant protease stability and synergy effects. In worldwide, 5–20% of AD patients are children^27^, for which steroidal drug, corticosteroids are commonly prescribed. However, prolonged intake of such steroidal drugs ends up with severe side effects and cutaneous atrophy^43^. Thus, the discovery of non-steroidal anti-inflammatory drug without side effects and short treatment duration are of highly imperative. Impressed by the anti-bacterial effects of TZP3 and TZP5, they were examined on AD-like skin lesions *in vitro* and *in vivo*. In RBL-2H3 cells, they inhibited *β*-hexosaminidase and tumor necrosis factor (TNF-*α*) release. In addition, these two polymers showed substantial effects in suppressing the histopathological changes of mice, including mast cell infiltration, cytokine expressions and protein levels of TGF-*β*, TNF-*α*, iNOS and COX-2, and serum IgE and IgG2a levels. Finally, the therapeutic potential of TZP3 and TZP5 were examined by treatment effects that displayed variations in the size of spleen and lymph node of AD-induced mice. Thus, our study demonstrates that these amphipathic triazine polymers could be a model for designing antibacterial agents that can also act as an anti-atopic agent, with promising therapeutic potential.

## Materials and Methods

### Chemistry

All reagents and starting materials were purchased from commercial chemical suppliers (Sigma-Aldrich, TCI and Across Organics) and used as received. All the anhydrous organic solvents of purity greater than 99.9% were purchased from Aldrich and used directly. Thin layer chromatography (TLC) was performed on Merck aluminum sheets with silica gel 60 F254 and were visualized by ultraviolet light, KMnO_4_ staining and ninhydrin. For the purification of compounds, column chromatography was performed on Merck silica gel 60 (70–230 mesh or 230–400 mesh). ^1^H and ^13^C NMR spectra were recorded on a Bruker DRX-400 and DRX-500 spectrometer. Chemical shifts (δ) are reported in parts per million (ppm) measured relative to an internal standard and coupling constants (*J*) are expressed in hertz (Hz). Mass spectra were recorded using Shimadzu (MALDI-TOF) mass spectrometer.

### Antimicrobial activity

The bacterial strains employed in the experiment were *Escherichia coli* (KCTC 1682), *Pseudomonas aeruginosa* (KCTC 1637), *Bacillus subtilis* (KCTC 3068), *Staphylococcus aureus* (KCTC 1621), three methicillin-resistant *Staphylococcus aureus* (CCARM 3089, 3090 and 3095), two multidrug-resistant *Pseudomonas aeruginosa* (CCARM 2095 and 2109) and vancomycin-resistant *Enterococcus faecium* (ATCC 51559). The antimicrobial activities of polymers were determined by the broth microdilution method, and the procedures were used as previously reported^44^. Briefly, bacteria grown to mid-logarithmic phase in LB broth were diluted to 2 × 10^6^ CFU/mL in 1% peptone and added to serial two-fold dilutions of different polymers ranging from 1 to 64 μg/mL. The plates were incubated in a shaking incubator at 37 °C for 18–22 h. The minimum inhibitory concentration (MIC) was determined by confirming the absence of bacterial growth both visually and spectrophotometrically by OD_600_ readings taken by a microplate ELISA reader (Molecular Devices, Sunnyvale, CA, USA). All measurements were performed three times.

### Hemolytic activity

Hemolytic activity was performed as described previously^11^. Sheep red blood cells (sRBCs) was washed three times with phosphate-buffered saline (PBS), centrifuged for 5 minutes at 1000 rpm, and diluted to a concentration of 2% in PBS. Both polymers solution (100 μL) and 2% sRBC (100 μL) were added simultaneously to each well of a 96-well plate and then further incubated at 37 °C for 2 h. After the plate was centrifuged at 120×g for 5 minutes, the supernatant (100 μL) from each well was transferred to a new 96-well plate and measured by a microplate ELISA reader (Molecular Devices, Sunnyvale, CA, USA) at 414 nm. Zero and 100% hemolysis were determined in PBS and 0.1% Triton-X 100, respectively.

### Protease stability

The protease stability of triazine based polymers (TZP3 and TZP5) was performed by the radial diffusion assay as described previously^11^. Briefly, a bacteria suspension (2×10^6^ CFU/mL in LB) was mixed with 0.7% agarose. This mixture was poured into a 10-cm petri dish, and it dispersed rapidly. 5μL of an aqueous TZP3, TZP5 or melittin stock solution (10 mg/mL) were added to 25 μL of trypsin solution (0.2 μg/mL) in PBS, and incubated at 37 °C for 4 h. The reaction was stopped by freezing with liquid nitrogen, after which 10 μL aliquots were placed in each circle paper, put on the agarose plates, and then incubated at 37 °C overnight. The diameters of the bacterial clearance zones surrounding the circle paper were measured for the quantitation of inhibitory activities.

### Chequerboard assay

Antimicrobial interactions between triazine based monomers (TZP3 and TZP5) and the clinically used antibiotics (chloramphenicol, ciprofloxacin and oxacillin) were evaluated via the chequerboard assay as described previously^32,45,46^. Briefly, 2-fold serial dilutions of each antibiotic and each polymer were prepared and added in a 1:1 volume ratio to the wells of a 96-well plate. An equal volume of bacterial solution (100 μL) at ∼10^6^ CFU/mL was then seeded into each well. The plates were incubated in a shaking incubator at 37 °C and 200 rpm and read after 22 h. Bacterial growth was assessed visually or spectrophotometrically via OD_600_ readings taken by a microplate ELISA reader (Molecular Devices, Sunnyvale, CA, USA). The fractional inhibitory concentration index (FICI) for each drug combination was calculated using the following equation: FICI = FIC_A_ + FIC_B_ = [A]/MIC_A_ + [B]/MIC_B_, where MIC_A_ and MIC_B_ are the MIC of antibiotic and triazine based monomer (TZP3 or TZP5), respectively, while [A] and [B] are the MIC of antibiotic and triazine based monomer (TZP3 or TZP5) in combination, respectively. FICI of ≤ 0.5 was interpreted as synergy, 0.5 < FICI ≤ 1.0 as additive, 1.0 < FICI ≤ 4.0 as indifferent, and a FICI > 4.0 as antagonism.

### Atopic dermatitis

Mouse IgE, IgG2a ELISA kits were obtained from Bethyl Laboratories (Montgomery, TX, USA). PCR primers were purchased from Genotech (Daejeon, Korea). Western blot antibodies, including transforming growth factor-*β* (TGF-*β*), tumor necrosis factor-*α* (TNF-*α*), inducible nitric oxide synthase (iNOS), cyclooxygenase-2 (COX-2) were acquired from Cell Signaling (Danvers, MA, USA). All other reagents, including calcium ionophore A23187, phorbol 12-myristate 13-acetate (PMA), 4-nitrophenyl-*N*-acetyl-*β*-D-glucosaminide, hydroxyethyl piperazineethanesulfonic acid (HEPES), l-glutamine, dimethyl sulfoxide (DMSO) were purchased from Sigma (St. Louis, MO, USA). DMEM cell medium, Fetal bovine serum (FBS), Penicillin/streptomycin and phosphate buffered saline (PBS) were provided by Gibco (CA, USA). RBL-2H3 cells from the Korean Cell Line Bank (Seoul, Korea).

### Cell culture

As we described in our previous article^47^, RBL-2H3 cells were cultured in Dulbecco’s modified Eagle’s medium (DMEM) supplemented with 10% fetal bovine serum, 1% penicillin/streptomycin (GIBCO). The cells were maintained in humidified conditions at 37 °C and 5% CO_2_ in an incubator.

### Production of TNF-α induced by A23187 and PMA

The inhibition of TNF-α release induced by A23187 and PMA from the RBL-2H3 cells was assessed by using a previously reported method^48^. The RBL-2H3 cells (2 × 10^5^ cells/well) were cultured with 1 μM A23187 and 80 nM PMA for 4 h at 37 °C, after exposure to 0, 15.625, 31.25, 62.5, or 125 μg/mL 30 min of the TZP3 and TZP5. Next, the cells were used for RT-PCR.

### *β*-hexosaminidase release assay

The *β*-hexosaminidase assay was performed as described previously with some modifications^49^. Cells were treated with different concentrations of samples for 12 h. The treated cells were washed two times with PBS and stimulated with calcium ionophore A23187 (1 μM, final concentration) at 37 °C for 30 min. Aliquots (50 μL) of the supernatants were incubated with 50 μL of 1 mM 4-nitrophenyl-N-acetyl-*β*-D-glucosaminide in 0.1 M sodium citrate (pH 4.5) at 37 °C for 1 h. At the end of incubation, 250 μL of carbonate buffer containing 0.1 M Na_2_CO_3_ and 0.1 M NaHCO_3_ (pH 10) was added, and then the absorbance due to the formation of *p*-nitrophenol was measured at 410 nm. The supernatant from the non-stimulated cells was used as a blank while the supernatant from the stimulated cells with calcium ionophore A23187 alone was used as a control. The *β*-hexosaminidase release levels were calculated as a percentage compared to that of control: *β*-hexosaminidase release (%) = (absorbance of tested sample− absorbance of blank)/(absorbance of control-absorbance of blank) × 100.

### Animals

As described in our previous article^50^, female BALB/c mice were purchased from the Nara Bio animal center (NARA Biotech, Seoul, Korea), housed under specific pathogen-free conditions. The mice were housed in groups of four in transparent plastic cages bedded with aspen chip and were provided with standard mouse chow diet and tap water ad libitum when not being treated. The environment of the animal room was carefully controlled, with a 12 h dark–light cycle, the temperature of 20–21 °C, and relative humidity of 40–45%. All experiments were approved by the Institutional Animal Care and Use Committee of Konkuk University (KU17088), and all experiments were performed in accordance with relevant guidelines and regulations.

### Allergic dermatitis model and treatment protocols

As described in our previous article^50^, the 7-week-old female BALB/c mice were divided into seven groups (n = 8 per group): Control (Con) group, TZP3 200 μg/mL (TZP3) group, and TZP5 200 μg/mL (TZP5) group, DNCB-sensitized only (AD) group, DNCB-sensitized + TZP3 200 μg/mL (AD + TZP3) group, DNCB-sensitized + TZP5 200 μg/mL (AD + TZP5) group, DNCB-sensitized + Dermatop (Sanofi, Korea) 0.25% (AD + Dermatop) group. Dermatop was used as a positive control treatment. For the first induction of AD in the BALB/c mice, the backs of the mice were shaved and challenged with 200 μL of 1% 2,4-dinitrochlorobenzene(DNCB) in acetone/olive oil (3:1) one time (day−4). After 4 days from the induction, 0.5% DNCB was painted onto the backs of the mice every two days (day 2, 4, 6, 8, 10, 12, 14, 16 and 18). Concentrations of 200 μg/mL TZP3 or TZP5 in 20 μL of PBS were applied to the dorsal skin once daily for 18 days. On day 18, all animals were sacrificed as shown in Fig. S4.

### Analysis of mouse blood

Blood was collected from each experimental group via cardiac puncture and was centrifuged at 3000 rpm and 4 °C for 20 min to obtain the serum. The levels of IgE, IgG2a were measured with an enzyme-linked immunosorbent assay kit (Bethyl Laboratories, Montgomery, TX, USA), following the manufacturer’s instructions.

### Histological observations

As described in our previous article^50^, the skin tissues of the mice were embedded in 10% neutral buffered formalin (NBF) for 24 h and embedded in white paraffin. Serial paraffin‐sections with 5 µm thickness, were prepared and stained with hematoxylin-eosin (H&E) or toluidine blue. The thickness of the epidermis and dermis was measured under a microscope. The number of mast cells stained by toluidine blue in the upper dermis in a field of 0.25 mm × 0.25 mm (0.0625 mm^2^) was counted under the microscope. The cells in five fields (0.3125 mm^2^ in total) of the upper dermis were counted, and an estimate of the number of cells per 1 mm^2^ was made for each mouse specimen.

### Evaluation of skin dermatitis severity score

The severity of dermatitis on the dorsal skin lesions was evaluated three times per week, according to a slight modification of the criteria described previously^51^. The development of (1) erythema/hemorrhage, (2) scarring/dryness, (3) edema, and (4) excoriation/erosion was scored as 0 (none), 1 (mild), 2 (moderate), or 3 (severe). The sum of the individual scores was defined as the dermatitis score.

### Analysis of mRNA expression

For the reverse transcription (RT) - polymerase chain reaction (PCR), the total cellular RNA was isolated from the dorsal skin tissue of each treatment group using TRIzol according to the manufacturer’s protocol. The first-strand complementary DNA (cDNA) was synthesized using Superscript II reverse transcriptase (Invitrogen). The conditions for RT-PCR were similar ones that have been previously described in related studies^38^. Quantitative real-time PCR was carried out using a Thermal Cycler Dice TP850 (Takarabio Inc., Shiga, Japan) according to the manufacturer’s protocol. Briefly, 2 μL of cDNA (100 ng), 1 μL of sense and antisense primer solution (0.4 µM), 12.5 μL of SYBR Premix Ex Taq (Takarabio Inc.), and 9.5 μL of dH_2_O were mixed to obtain a final 25-μL reaction mixture in each reaction tube. The amplification conditions were as follows: 10 s at 95 °C, 40 cycles of 5 s at 95 °C and 30 s at 60 °C, 15 s at 95 °C, 30 s at 60 °C, and 15 s at 95 °C. The mRNA levels of the target genes relative to that of GAPDH were normalized as follows: relative mRNA expression = 2^-(ΔCt of target gene−ΔCt of GAPDH)^, where *Ct* is the threshold cycle value. In each sample, the expression level of the analyzed gene was normalized to that of GAPDH and presented as a relative mRNA level.

### Western blot analysis

Frozen skins from the mice were homogenized in liquid nitrogen. For the western blot analysis, the tissues were lysed in a RIPA lysis buffer(Sigma) containing 1% protease inhibitor. The cell lysates were incubated on ice for 1 h and then the homogenates were centrifuged at 13000 rpm for 20 min at 4 °C. The cell lysates (30 μg protein/sample) were separated by 10% sodium dodecyl sulfate-polyacrylamide gel electrophoresis (SDS-PAGE) and transferred to a nitrocellulose blotting (DC, Invitrogen) membrane. The DC membrane was blocked with 5% skim milk and incubated overnight at 4 °C with several primary antibodies (diluted 1:5000). Anti-COX-2, anti-iNOS, anti-TNF-*α*, anti-TGF-*β* were purchased from Cell Signaling Technology (Danvers, MA, USA). After the membranes were washed, they were incubated with anti-rabbit IgG HRP-conjugated secondary antibodies (1:2000) for 2 h at room temperature. Equal protein loading was ascertained using Ponceau S staining of the blotted membranes as well as western blotting with a *β*-actin antibody. The antibody-specific proteins were visualized with enhanced chemiluminescence (ECL) detection reagent and quantified using the ImageJ software. All the full length blots are included in the supplementary informations.

### Statistical analysis

Statistical analysis was carried out using SAS statistical software (SAS Institute, Cary, NC, USA). Multiple group data were analyzed using one-way analysis of variance followed by Dunnett’s multiple range test. All results are expressed as the mean ± standard deviation of comparative fold differences. Data are representative of three independent experiments. The threshold for significance was set at *p* < 0.05.

## Supplementary information

supplementary information

## Data Availability

All data generated or analyzed during this study are included in this article and its supplementary information files.
