# Supplementary material for: Amphiphilic Triazine Polymer Derivatives as Antibacterial And Anti-atopic Agents in Mice Model
Source: Sci Rep. 2019 Oct 22;9:15161. doi: 10.1038/s41598-019-51561-7 (PMC6805867; doi:10.1038/s41598-019-51561-7)
Supplement: Supplementary file 1 — supplementary information [file 41598_2019_51561_MOESM1_ESM.pdf]

Amphiphilic Triazine Polymer Derivatives as Antibacterial And Anti-atopic Agents in Mice Model

Pethaiah Gunasekaran[1,+], meiqi Fan[2,+], Eun Young Kim[3,+], Jun Ho Shin[1], Ji Eun Lee[1,5], Eun Ju Son[1], Jaehi Kim[1], Eunha Hwang[1], Min Su Yim[1,5], Eun-Hee Kim[1], Young-Jin Choi[2], Young-Ho Lee[1,5], Young-Ho Chung[4], Hak Nam Kim[1], Eun Kyoung Ryu[1,5], Song Yub Shin*[,3], Eun-Kyung Kim*[,2], Jeong Kyu Bang*[,1,5]

[1]Division of Magnetic Resonance, Korea Basic Science Institute (KBSI), Ochang, Chung Buk, 28119, Republic of Korea. E-mail: bangjk@kbsi.re.kr, Tel: +82-43-240-5023, Fax: +82-43-240-5059

[2]Division of Food Bioscience, Konkuk University, Chungju 27478, Republic of Korea

[3]Department of Medical Science, Graduate School, and Department of Cellular and Molecular Medicine, School of Medicine, Chosun University, Gwangju 61452, Republic of Korea

[4]Drug & Disease Target Research Team, Korea Basic Science Institute (KBSI), Ochang, Chung Buk, 28119, Republic of Korea

[5]Department of Bio-analytical Science, University of Science & Technology, Daejeon, 34113, Republic of Korea

Table of Content

No	Content	Page
1	Full-length blots correspond to figure 6 and figure S2	S3-S4
2	Effect of TZP3 and TZP5 on TNF-*α* suppression	S5
3	Effect of TZP3 and TZP5 on *β*-hexosaminidase suppression	S5
4	Cytotoxicity evaluation of TZP3 and TZP5 on HaCaT cells	S6
5	Allergic dermatitis model and treatment protocols.	S7
6	Experimental section	S7-S14
7	^{1}H & ^{13}C NMR spectra of synthesised compounds and polymers	S15-S65

Full-length blots correspond to figure 6

No	Name	Full length image
1	**Actin, TZP3**	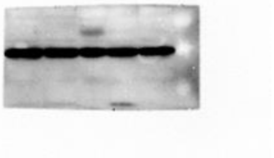
2	**Actin, TZP5**	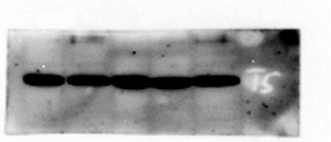
3	**COX-2 TZP3**	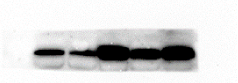
4	**COX-2 TZP5**	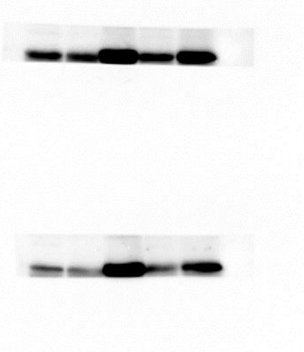
5	**INOS TZP3**	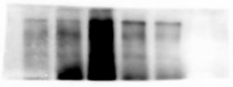
6	**INOS TZP5**	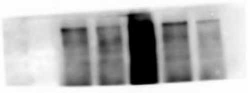
7	**TGF-β TZP-3**	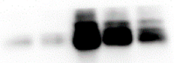
8	**TGF-β TZP-5**	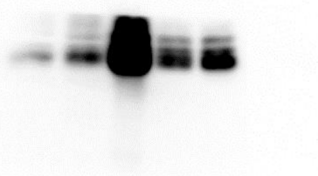
9	**TNF-α TZP-3**	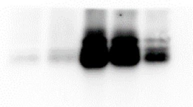

10	**TNF-α TZP-5**	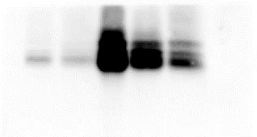

Full-length blots correspond to figure S2

1. **β-Actin, TZP3, and TZP5**

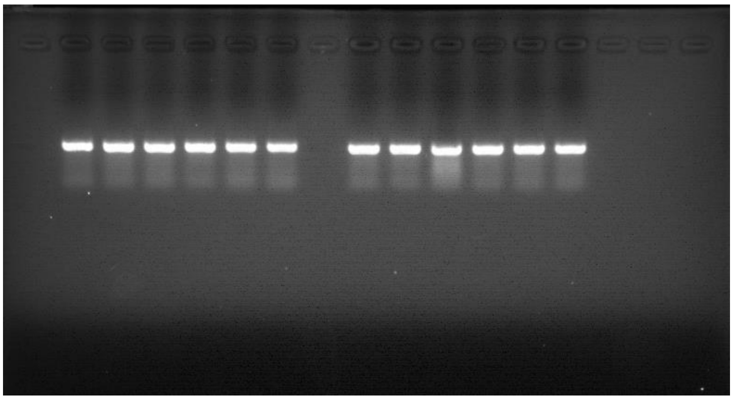

2. **TNF- α, TZP3, and TZP5**

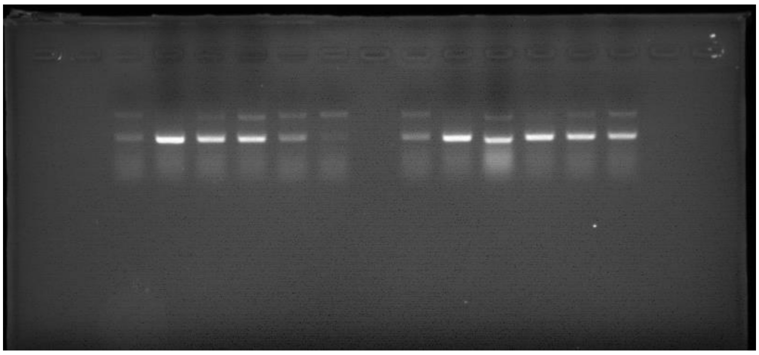

Effect of TZP3 and TZP5 on TNF-*α* suppression

Therefore, the dimer TZP3 and trimer TZP5 were probed for their potential for inhibiting the TNF-*α* induced from RBL-2H3 cells in *in vitro* method. Calcium ionophore A23187 and PMA induced the TNF-*α* secretion in RBL-2H3 cells, on the contrary, treatment of which with TZP3 and TZP5 suppressed the TNF-*α* secretion in a dose-dependent manner, as shown in figure S2. Even though, 15.6 and 31.2 μg/mL of TZP3 displayed almost similar TNF-*α* suppressions (10%), treatment of 62.5 and 125 μg/mL of TZP3 showed significant effects in TNF-*α* suppressions, approximately 70% and 90%, respectively. Similarly, 15.6 and 31.2 μg/mL of TZP5 didn't show appreciable TNF-*α* suppressions, whereas treatment of 62.5 and 125 μg/mL displayed TNF-*α* suppressions in a dose-dependent manner.

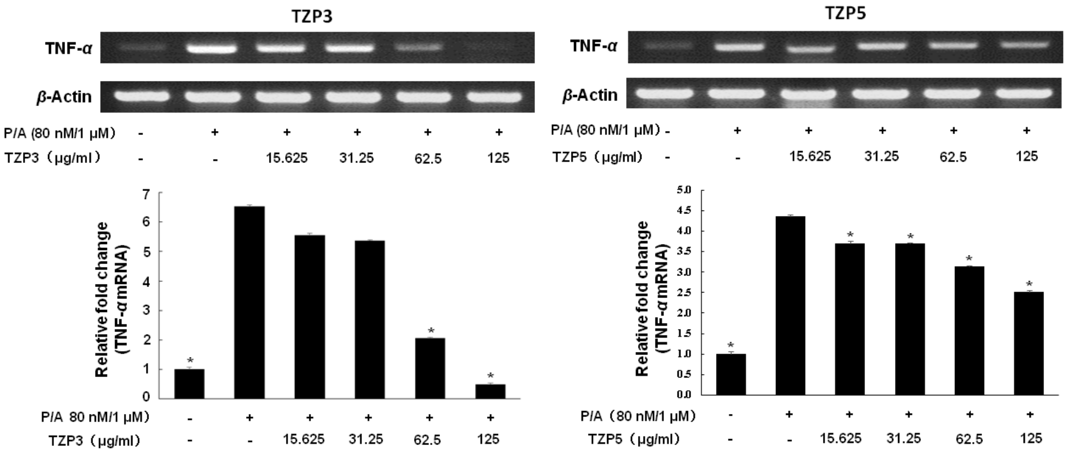

Figure S1. Inhibitory effects of the TZP3 and TZP5 on A23187 and PMA-induced TNF-*α* induced from RBL-2H3 cells. ***(Full-length blots are included in in the above section).***

Effect of TZP3 and TZP5 on *β*-hexosaminidase suppression

Treatment with calcium ionophore A23187 in the RBL-2H3 cells induced the degranulation by releasing *β*-hexosaminidase as shown in figure S3. Treatment of TZP3 and TZP5 against *β*-hexosaminidase showed a significant effect in a dose-dependent manner. Especially at a concentration of 500 μg/mL, they showed maximum inhibitory effects.

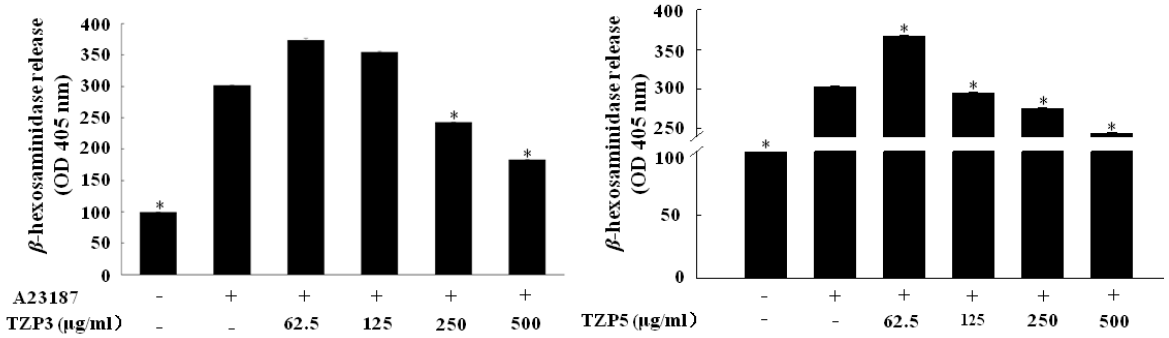

Figure S2. Effect of TZP3 and TZP5 on *β*-hexosaminidase release in RBL-2H3 cells

Cytotoxicity evaluation of TZP3 and TZP5 on HaCaT cells

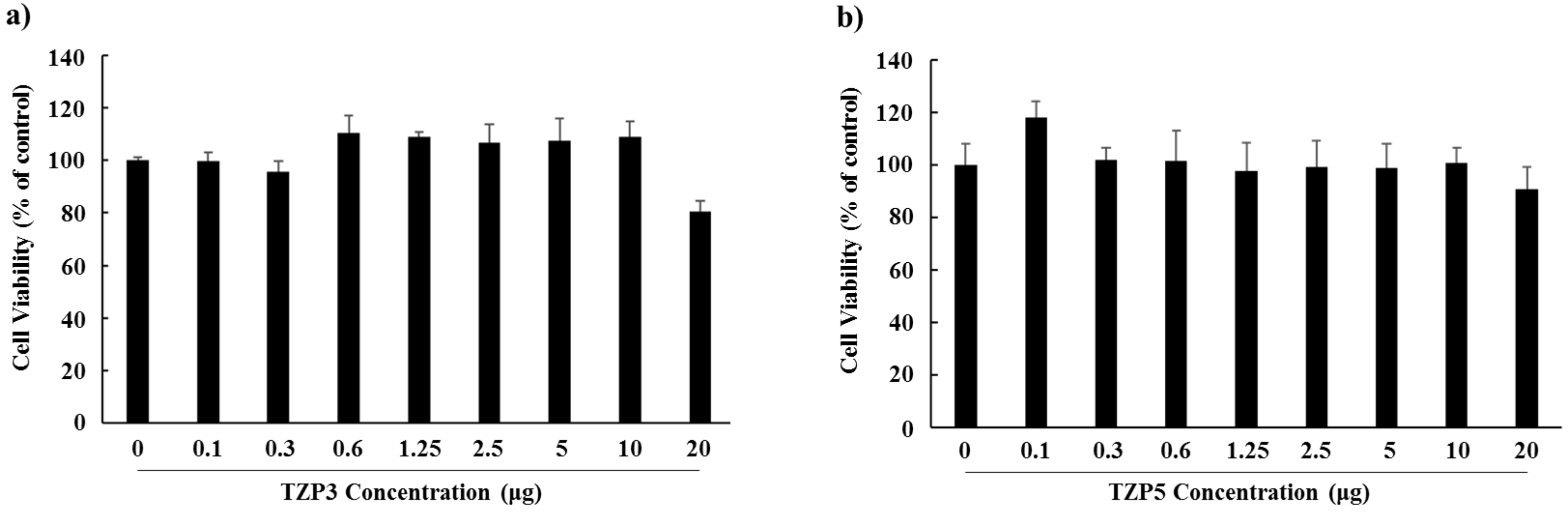

Figure S3: Cytotoxicity evaluation of **TZP3 (b)** and **TZP5 (b)** on HaCaT cells. **TZP3** and **TZP5** were treated with HaCaT cells in various concentrations for 24 h followed by MTT assay, and absorbance was measured. Cell viability was calculated as the relative absorbance.

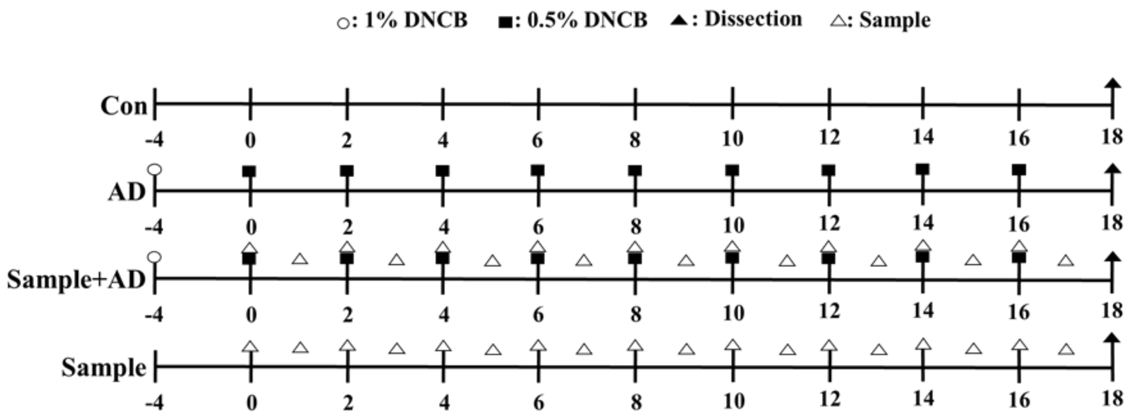

Figure S4: Allergic dermatitis model and treatment protocols.

Experimental section

Synthesis of **2**

N,N'-di-boc-*N*-trifylguanidine (4.27 g, 0.011 mol, 2.05 equiv.) in anhydrous DCM (30 mL) was added slowly to a stirred solution of norspermidine (**1**) (0.7 g, 0.005 mol, 2.05 equiv.) and triethylamine (3.0 mL, 0.021 mol, 4 equiv.) in DCM (20 mL) at 0 °C. After completion of the addition, the temperature was slowly raised to room temperature and stirred for 6 h. Then the reaction mixture was quenched by the addition of water (30 mL) and extracted with DCM (30 mL x 3). The combined organic extracts were washed with brine (40 mL), dried over Na_2SO_4, and evaporated. The crude product was purified by silica gel column chromatography using DCM:MeOH:TEA (20:1:0.1) mixture to afford **2** as pale brown color viscous oil (2.25g, 68%). ^{1}H NMR (400 MHz, $CDCl_3$) δ 11.49 (s, 2H), 8.56 (s, 2H), 3.51 (q, *J* = 6.2 Hz, 4H), 2.67 (t, *J* = 6.7 Hz, 4H), 1.77 (t, *J* = 6.7 Hz, 4H), 1.49 (d, *J* = 4.2 Hz, 36H). ^{13}C NMR (100 MHz, $CDCl_3$) δ 163.6, 156.2, 153.1, 82.9, 79.1, 47.4, 39.2, 29.2, 28.3, 28.1. Maldi-tof *m/z* calcd for $C_{28}H_{53}N_7O_8$: 615.3, found 616.3 $(M+H)^+$

Synthesis of **3**

To a stirred of solution of 2,4,6-trichloro-1,3,5-triazine (cyanuric chloride) (0.89g, 0.005 mol, 1.2 equiv.) and DIEA (2.2 mL, 0.012 mol, 3 equiv.) in 20 mL DCM at 0 °C, **2** (2.50 g, 0.004 mol, 1 equiv.) in 20 mL DCM was added slowly by ensuring that temperature was maintained at 0 °C. After the addition was completed, further stirred at the same temperature for 3 h. The reaction mixture was quenched with water (30 mL) and extracted from DCM (2 x 20 mL). The combined organic layer extracts were washed with brine (30 mL) and dried over Na_2SO_4 and evaporated. The crude product was purified by column chromatography using DCM/MeOH (98:2) combination to obtain the shiny, pale yellow solid as a product **3** (3g, 97%).^{1}H NMR (400 MHz, $CDCl_3$) δ 11.48 (s, 2H), 8.44 (s, 2H), 3.66 (t, *J* = 7.1 Hz, 4H), 3.47 (q, *J* = 6.4 Hz, 4H), 1.91 (p, *J* = 6.9 Hz, 4H), 1.50 (d, *J* = 2.4 Hz, 36H). ^{13}C NMR (100 MHz, $CDCl_3$) δ 170.1, 164.9, 163.5, 156.2, 153.2, 83.2, 79.3, 45.3, 38.0, 28.3, 28.1, 26.9.

Synthesis of tert-butyl 3,3'-azanediylbis(propane-3,1-diyl) dicarbamate (**4**)

To a stirred solution of norspermidine (**1**) (7.05 mL, 0.051 mol, 1.0 equiv.) and DIEA (25 mL, 0.142 mol, 2.8 equiv.) in THF (125 mL) at 0 ^{o}C, was added 1-(tert-butoxycarbonyloxyimino)-2- phenylacetonitrile (BOC-ON) (25g, 0.100 mol, 2.0 equiv.) in THF (75 mL) dropwise over 60 minutes. After the addition, the resultant mixture was stirred at 0 ^{o}C for three hours and warmed to room temperature, and stirred for 20 h at room temperature. The solvent was evaporated under vacuo, the resultant crude residue was redissolved in DCM (100 mL) and washed with 10% NaOH (3x60 mL) followed by brine (100 mL) and dried over Na_2SO_4. The oily product formed after evaporation was treated with hexane (125 mL) and methanol (1 mL) and kept in the freezer for overnight. The resultant white solid was filtered and washed with hexane, and dried to yield the product **4** as a white solid (13 g, 81%). ^{1}H NMR (400 MHz, $CDCl_3$) δ 5.19 (s, 2H), 3.21 (q, *J* = 6.3 Hz, 4H), 2.65 (t, *J* = 6.5 Hz, 4H), 1.65 (p, *J* = 6.5 Hz, 4H), 1.44 (s, 18H). ^{13}C NMR (100 MHz, $CDCl_3$) δ 156.1, 78.9, 47.4, 38.9, 29.8. Maldi-tof *m/z* calcd for $C_{16}H_{33}N_3O_4$: 331.2, found 332.2 $(M+H)^+$

Synthesis of tert-butyl 3,3'-(4,6-dichloro-1,3,5-triazin-2-ylazanediyl)bis(propane-3,1-diyl)dicarbamate (**5**)

To a stirred solution of 2,4,6-trichloro-1,3,5-triazine (cyanuric chloride) (1 g, 5.43 mmol, 1 equiv.) and DIEA (2.8 mL, 16.3 mmol, 3 equiv.) in DCM (30 mL) at 0 ^{o}C, was added **4** (1.83g, 5.54 mmol, 1.02 equiv.) in DCM (20 mL) drop wise over 30 min, ensuring that the temperature was maintained at 0 ^{o}C, and stirred further at 0 ^{o}C for 3 h. The reaction mixture was quenched with water (50 mL) and extracted from DCM (2 x 30 mL). The combined organic layer extracts were washed with brine (50 mL) and dried over Na_2SO_4 and evaporated. The resultant residue was purified by silica gel column

chromatography (Hexane-Ethyl acetate, 3:1) to afford **5** as a white solid (2.26g, 87%). ^{1}H NMR (400 MHz, $CDCl_3$) δ 5.05 (t, *J* = 6.0 Hz, 2H), 3.63 (t, *J* = 6.9 Hz, 4H), 3.13 (q, *J* = 6.4 Hz, 4H), 1.81 (p, *J* = 6.7 Hz, 4H), 1.45 (s, 18H). ^{13}C NMR (100 MHz, $CDCl_3$) δ 170.1, 164.7, 156.0, 79.3, 45.0, 37.4, 28.4, 27.7. Maldi-tof *m/z* calcd for $C_{19}H_{32}Cl_2N_6O_4$: 478.1, found 501.2 $(M+Na)^+$

Synthesis of N,N-dibenzyl-4,6-dichloro-1,3,5-triazin-2-amine (**6a**)

To a stirred solution of 2,4,6-trichloro-1,3,5-triazine (cyanuric chloride) (0.50 g, 2.72 mmol, 1 equiv.) and DIEA (2.5 mL, 13.5 mmol, 5 equiv.) in DCM (15 mL) at 0 ^{o}C, was added dibenzylamine (0.49 mL, 2.58 mmol, 0.95 equiv.) in DCM (10 mL) dropwise over 30 min, ensuring that the temperature was maintained at 0 ^{o}C, and stirred further at 0 ^{o}C for 3 h. The reaction mixture was quenched with water (10 mL) and extracted from DCM (2 x 15 mL). The combined organic layer extracts were washed with brine (50 mL) and dried over Na_2SO_4 and evaporated. The resultant residue was purified by silica gel column chromatography (Hexane-Ethyl acetate, 9:1 to 8:2) to afford **6a** as a white solid (0.63g, 67 %). ^{1}H NMR (400 MHz, $CDCl_3$) δ 7.47 – 7.31 (m, 6H), 7.31 – 7.20 (m, 4H), 4.82 (s, 4H). ^{13}C NMR (101 MHz, $CDCl_3$) δ 170.54, 165.60, 135.38, 128.91, 128.11, 49.14. Maldi-tof *m/z* calcd for $C_{17}H_{14}Cl_2N_4$: 344.07, found 345.22 $(M+H)^+$

Synthesis of 4,6-dichloro-N-(3,3-diphenylpropyl)-1,3,5-triazin-2-amine (**6b**)

To a stirred solution of 2,4,6-trichloro-1,3,5-triazine (cyanuric chloride) (0.50 g, 2.72 mmol, 1 equiv.) and DIEA (2.5 mL, 13.5 mmol, 5 equiv.) in DCM (15 mL) at 0 ^{o}C, was added 3,3-diphenylpropylamine (0.54g, 2.58 mmol, 0.95 equiv.) in DCM (10 mL) drop wise over 30 min, ensuring that the temperature was maintained at 0 ^{o}C, and stirred further at 0 ^{o}C for 3 h. The reaction mixture was quenched with water (10 mL) and extracted from DCM (2 x 15 mL). The combined organic layer extracts were washed with brine (50 mL) and dried over Na_2SO_4 and evaporated. The resultant residue was purified by silica gel column chromatography (Hexane-Ethyl acetate, 10:1) to afford **6b** as a white solid (0.52g, 54 %). ^{1}H NMR (400 MHz, $CDCl_3$) δ 7.41 – 7.13 (m, 10H), 6.56 (s, 1H), 4.01 (t, *J* = 7.9 Hz, 1H), 3.50 (q, *J* = 6.7 Hz, 2H), 2.42 (q, *J* = 7.6 Hz, 2H). ^{13}C NMR (101 MHz, $CDCl_3$) δ 170.86, 169.55, 165.79, 143.62, 128.84, 127.67, 126.66, 48.85, 40.37, 34.52. Maldi-tof *m/z* calcd for $C_{18}H_{16}Cl_2N_4$: 358.07, found 359.24 $(M+H)^+$

General Procedure for TZP polymer synthesis

Rink amide 4-methylbenzhydrylamine (MBHA) resin was purchased from Calbiochem-Novabiochem (La Jolla, CA). All the TZP polymers were synthesized from monosubstituted triazine building blocks anchoring various substituents to maintain either hydrophobicity or hydrophilicity by solid-phase peptide synthesis method using Rink amide resin with an initial loading of 0.61 mmol/g. Prior to the sequence extension, the resin was swollen in DMF for 45 min and a mixture of mono-substituted *s*-triazine (10 equivalents) and *N*,*N*-diisopropylethylamine (10 equivalents) in DMF(2 mL) was added and stirred for 2 hours at room temperature. Then, the resin was filtered and washed with DMF (3 times). Further to introduce the linker, a mixture of ethylenediamine (20 equivalents), *N*,*N*-diisopropylethylamine (10 equivalents) in DMF(2 mL) was added into the resin and was shaken for 3h at 60 ^{o}C. The resin was filtered and washed with DMF (3 times), and the process was repeated until synthesizing the desired polymer. Finally, the resin was washed with dichloromethane, and ether, and dried under vacuum. The polymer was cleaved from the resin using a mixture of trifluoroacetic acid, water, and triisopropylsilane (90:5:5, v/v/v, 2 mL) for 2 h. The crude polymer was precipitated by the addition of cold diethyl ether and filtered. Subsequently, the crude polymer was purified using preparative Vydac C_{18} column using a 10-90% for 30 min in water/acetonitrile gradient in the presence of 0.05% TFA (A: water buffer, B: acetonitrile buffer). The purity of the polymer (>95%) was assessed by RP- HPLC on an analytical Vydac C_{18} column. The polymers mass were confirmed using MALDI-TOF-MS (Shimadzu) and the structures were further confirmed using NMR

TZP1

^{1}H NMR (500 MHz, MeOD) δ 8.03 (s, 1H), 3.82 – 3.58 (m, 12H), 3.29 – 3.05 (m, 1H), 3.08 – 2.94 (m, 8H), 2.26 – 1.73 (m, 8H). ^{13}C NMR (101 MHz, MeOD) δ 184.59, 181.93, 173.37, 169.13, 168.36, 167.22, 166.14, 165.56, 165.08, 164.70, 163.40, 162.24, 161.89, 161.55, 161.20, 158.88, 157.04, 156.49, 155.85, 155.70, 152.35, 123.92, 121.65, 121.08, 120.56, 119.74, 118.17, 117.46, 115.26, 113.14, 112.35, 110.97, 54.41, 44.30, 44.09, 43.67, 43.53, 43.35, 42.80, 42.66, 42.37, 40.78, 39.81, 38.80, 37.19, 36.63, 36.52, 36.39, 25.70, 25.60, 25.41, 25.02. (Rotomer existence is present, as there is line brodening and presence of multiple signals). Maldi-tof *m/z* calcd for $C_{20}H_{40}ClN1_{5}$ 525.32, found 526.33 $(M+H)^{+}$.

TZP2

^{1}H NMR (500 MHz, MeOD) δ 7.38 – 7.25 (m, 6H), 7.24 – 7.16 (m, 4H), 4.76 – 4.70 (m, 3H), 4.70 – 4.63 (m, 1H), 3.75 – 3.48 (m, 8H), 3.27 (t, *J* = 6.8 Hz, 1H), 3.23 (t, *J* = 6.8 Hz, 1H), 3.18 (t, *J* = 6.8 Hz, 1H), 3.12 (t, *J* = 6.9 Hz, 1H), 2.06 – 1.95 (m, 1H), 1.95 – 1.88 (m, 1H), 1.89 – 1.80 (m, 1H),

1.80 – 1.68 (m, 1H). ^{13}C NMR (201 MHz, MeOD) δ 169.53, 169.00, 166.66, 166.07, 165.67, 161.58, 161.41, 157.28, 156.67, 137.21, 137.08, 136.95, 128.33, 128.22, 127.62, 127.56, 127.38, 127.33, 127.22, 127.12, 127.02, 48.51, 48.44, 48.29, 48.09, 47.98, 44.95, 44.75, 44.44, 40.75, 40.32, 39.40, 39.01, 38.82, 38.67, 38.50, 26.76, 26.68 (rotomer existence is present and traces of DMSO is present). Maldi-tof *m/z* calcd for $C_{30}H_{42}ClN_{17}$ 675.35, found 676.35 $(M+H)^+$.

TZP3

^1H NMR (500 MHz, MeOD) δ 7.41 – 7.27 (m, 7H), 7.28 – 7.13 (m, 5H), 4.65 (s, 2H), 4.48 (s, 2H), 3.66 – 3.57 (m, 2H), 3.55 – 3.48 (m, 2H), 3.45 (t, *J* = 6.5 Hz, 2H), 3.37 (t, *J* = 6.9 Hz, 2H), 2.96 – 2.90 (m, 1H), 2.90 – 2.82 (m, 2H), 2.73 – 2.63 (m, 2H), 1.95 – 1.87 (m, 1H), 1.87 – 1.75 (m, 2H), 1.65 – 1.53 (m, 2H). ^{13}C NMR (100 MHz, MeOD) δ 169.51, 168.91, 166.63, 165.95, 165.64, 165.56, 163.34, 162.12, 161.78, 161.43, 161.08, 156.87, 155.53, 137.23, 137.12, 136.98, 128.33, 128.22, 127.65, 127.58, 127.39, 127.26, 127.15, 127.01, 118.08, 115.18, 48.60, 44.24, 43.64, 40.71, 40.15, 39.54, 38.83, 37.23, 37.02, 36.50, 25.76, 25.55, 25.37. (rotomer existence is present). Maldi-tof *m/z* calcd for $C_{28}H_{38}ClN1_3$ 591.30, found 592.37 $(M+H)^+$.

TZP4

^1H NMR (400 MHz, MeOD) δ 7.41 – 7.14 (m, 10H), 4.81 – 4.68 (m, 4H), 3.84 – 3.41 (m, 16H), 3.19 – 2.81 (m, 9H), 2.20 – 1.76 (m, 9H). ^{13}C NMR (201 MHz, MeOD) δ 169.49, 168.92, 166.63, 165.89, 165.65, 163.41, 162.93, 162.79, 161.87, 161.70, 161.52, 161.35, 156.96, 155.81, 155.46, 137.17, 137.07, 128.30, 128.23, 127.67, 127.52, 127.39, 127.23, 127.10, 127.00, 118.80, 117.35, 115.90, 114.45, 48.73, 48.24, 44.67, 44.37, 43.71, 40.76, 39.95, 39.57, 39.24, 39.16, 38.83, 37.12, 36.54, 25.65, 25.42 (rotomer existence is present). Maldi-tof *m/z* calcd for $C_{39}H_{60}ClN_{21}$ 857.50, found 858.50 $(M+H)^+$.

TZP5

^1H NMR (500 MHz, MeOD) δ 7.40 – 7.20 (m, 8H), 7.21 – 7.08 (m, 2H), 4.06 – 3.94 (m, 1H), 3.81 – 3.45 (m, 16H), 3.47 – 3.35 (m, 2H), 3.17 – 2.92 (m, 8H), 2.49 – 2.27 (m, *J* = 7.0 Hz, 2H), 2.15 – 1.86 (m, *J* = 28.6 Hz, 8H). ^{13}C NMR (176 MHz, MeOD) δ 169.16, 168.58, 165.09, 164.76, 162.86, 162.01, 161.88, 161.68, 161.48, 161.28, 156.40, 144.44, 128.16, 127.45, 127.41, 125.97, 119.15, 117.49, 115.82, 114.16, 48.83, 48.69, 48.13, 44.61,

44.17, 43.96, 43.38, 42.83, 39.72, 39.51, 39.35, 39.02, 38.92, 37.26, 36.68, 36.42, 34.62, 34.47, 34.15, 25.73, 25.58, 25.33, 25.04 (rotomer existence is present). Maldi-tof *m/z* calcd for $C_{40}H_{62}ClN_{21}$ 871.51, found 872.51 $(M+H)^+$.

TZP6

^{1}H NMR (400 MHz, D_2O) δ 7.44 – 7.20 (m, 6H), 7.17 – 6.94 (m, 4H), 4.25 (s, 2H), 4.09 (s, 2H), 3.79 – 3.55 (m, 4H), 3.52 – 3.07 (m, 12H), 3.06 – 2.83 (m, 4H), 2.79 (t, *J* = 6.5 Hz, 2H), 2.64 (t, *J* = 6.9 Hz, 2H), 2.04 – 1.85 (m, 1H), 1.79 – 1.63 (m, 2H), 1.56 – 1.21 (m, 6H). ^{13}C NMR (176 MHz, MeOD) δ 169.18, 168.58, 165.40, 164.74, 163.40, 161.82, 161.62, 161.42, 161.22, 157.22, 156.08, 155.23, 136.44, 128.51, 127.57, 127.43, 127.15, 119.05, 117.39, 115.73, 114.07, 44.47, 44.28, 43.97, 43.56, 40.36, 39.73, 38.71, 38.36, 37.06, 36.47, 26.73, 26.40, 25.60, 25.37 (rotomer existence is present). Maldi-tof *m/z* calcd for $C_{41}H_{64}ClN_{25}$ 941.54, found 942.63 $(M+H)^+$.

TZP7

^{1}H NMR (400 MHz, MeOD) δ 3.89 – 3.47 (m, 10H), 3.24 – 3.10 (m, 1H), 3.11 – 2.86 (m, 6H), 2.22 – 1.92 (m, 6H). ^{13}C NMR (101 MHz, MeOD) δ 169.16, 166.05, 165.08, 163.36, 162.87, 162.19, 161.85, 161.50, 161.15, 156.85, 155.65, 120.98, 118.08, 115.17, 112.27, 94.94, 44.68, 44.39, 43.72, 43.37, 42.82, 42.67, 40.51, 39.64, 39.33, 39.16, 38.85, 37.10, 36.63, 36.52, 36.39, 25.65, 25.40, 25.20, 25.02 (rotomer existence is present). Maldi-tof *m/z* calcd for $C_{31}H_{62}ClN_{23}$ 791.52, found 792.58 $(M+H)^+$.

TZP8

^{1}H NMR (400 MHz, MeOD) δ 7.51 – 7.03 (m, 20H), 4.84 – 4.58 (m, 8H), 3.78 – 3.39 (m, 12H), 3.09 – 2.77 (m, 5H), 2.09 – 1.73 (m, 5H). ^{13}C NMR (176 MHz, MeOD) δ 172.40, 170.79, 168.80, 165.57, 161.91, 161.71, 161.51, 156.98, 137.18, 137.02, 136.78, 136.73, 136.53, 136.25, 128.39, 128.30, 128.24, 127.75, 127.67, 127.55, 127.38, 127.29, 127.23, 127.12, 127.03, 117.49, 115.82, 49.19, 48.64, 44.47, 41.03, 39.31, 37.36, 36.93, 35.89, 29.34, 25.57, 24.85 (rotomer existence is present and traces of DMSO is present). Maldi-tof *m/z* calcd for $C_{47}H_{58}ClN_{19}$ 923.48, found 924.61$(M+H)^+$.

TZP9

^{1}H NMR (400 MHz, D_2O) δ 7.43 – 7.17 (m, 6H), 7.16 – 6.90 (m, 4H), 4.21 (s, 2H), 4.09 (s, 2H), 3.79 – 3.58 (m, 4H), 3.53 – 3.08 (m, 12H), 3.04 – 2.66 (m, 8H), 1.58 – 1.21 (m, 8H). ^{13}C NMR (201 MHz, MeOD) δ 168.56, 166.15, 165.39, 164.04, 162.11, 161.94, 161.77, 161.59, 157.25, 128.52, 127.45, 127.24, 118.87, 117.41, 115.96, 114.51, 48.09, 47.98, 45.26, 44.80, 44.47, 43.96, 43.82, 43.59, 40.49, 40.36, 40.03, 39.01, 38.71, 38.62, 38.48, 28.99, 26.65, 26.38 (rotomer existence is present and traces of DMSO is present). Maldi-tof *m/z* calcd for $C_{43}H_{68}ClN_{29}$ 1025.59, found 1026.62 $(M+H)^+$.

TZP10

^{1}H NMR (700 MHz, MeOD) δ 7.41 – 7.24 (m, 8H), 7.23 – 7.09 (m, 2H), 4.08 – 3.94 (m, 1H), 3.83 – 3.46 (m, 16H), 3.47 – 3.39 (m, 2H), 3.31 – 3.14 (m, 8H), 2.46 – 2.30 (m, 2H), 2.04 – 1.79 (m, 8H). ^{13}C NMR (176 MHz, MeOD) δ 169.11, 168.52, 165.48, 164.88, 162.56, 162.04, 161.84, 161.64, 161.44, 157.31, 156.43, 155.41, 144.41, 128.15, 127.43, 125.97, 119.10, 117.44, 115.78, 114.12, 48.87, 48.58, 45.07, 44.49, 44.08, 43.80, 39.73, 39.51, 39.36, 38.81, 38.56, 38.46, 34.56, 34.10, 26.81, 26.46 (rotomer existence is present). Maldi-tof *m/z* calcd for $C_{44}H_{70}ClN_{29}$ 1039.60, found 1040.68$(M+H)^+$

TZP11

^{1}H NMR (500 MHz, MeOD) δ 7.35 – 7.03 (m, 20H), 3.98 (s, 2H), 3.73 – 3.45 (m, 20H), 3.42 – 3.33 (m, 4H), 3.28 – 3.15 (m, 8H), 2.67 (s, 1H), 2.47 – 2.24 (m, 4H), 2.00 – 1.80 (m, 8H). ^{13}C NMR (176 MHz, MeOD) δ 169.17, 168.49, 164.86, 163.81, 162.15, 161.95, 161.75, 161.56, 157.29, 157.24, 156.13, 144.39, 144.30, 128.16, 127.43, 125.96, 119.16, 117.51, 115.85, 114.24, 48.87, 48.61, 48.45, 45.05, 43.78, 43.65, 39.70, 39.36, 39.01, 38.82, 38.59, 38.46, 34.56, 34.41, 34.12, 29.34, 26.80, 26.50 (rotomer existence is present and traces of DMSO is present) . Maldi-tof *m/z* calcd for $C_{64}H_{92}ClN_{35}$ 1385.79, found 1386.90$(M+H)^+$.

TZP12

^{1}H NMR (500 MHz, MeOD) δ 7.42 – 7.03 (m, 21H), 4.07 – 3.93 (m, 2H), 3.79 – 3.34 (m, 24H), 3.09 – 2.84 (m, 8H), 2.67 (s, 1H), 2.46 – 2.24 (m, 4H), 2.10 – 1.92 (m, 8H). ^{13}C NMR (101 MHz, MeOD) δ 166.15, 165.18, 164.28, 162.23, 161.88, 161.53, 161.40, 161.18, 144.31, 136.02, 132.84, 128.15, 127.44, 125.97, 120.82, 119.57, 118.08, 116.46, 115.17, 48.69, 48.62, 44.73, 43.32, 42.83, 39.36, 39.27, 38.77, 37.22, 37.12, 36.73, 36.62, 34.55, 34.50, 33.64, 25.69, 25.59, 24.96, 24.69 (rotomer existence is present). Maldi-tof *m/z* calcd for $C_{60}H_{84}ClN_{27}$ 1217.70, found 1218.70(M+H)$^+$.

TZP13

^{1}H NMR (400 MHz, D_2O) δ 3.53 (d, *J* = 31.1 Hz, 33H), 3.17 – 2.79 (m, 23H), 2.05 – 1.80 (m, 20H). ^{13}C NMR (101 MHz, MeOD) δ 169.14, 165.07, 163.35, 162.87, 162.38, 162.03, 161.69, 161.34, 156.87, 155.49, 121.04, 118.13, 115.22, 112.32, 44.72, 44.41, 43.72, 39.22, 37.10, 36.50, 25.64, 24.00 (rotomer existence is present). Maldi-tof *m/z* calcd for $C_{53}H_{106}ClN_{39}$ 1323.91, found 1325.12 (M+H)$^+$

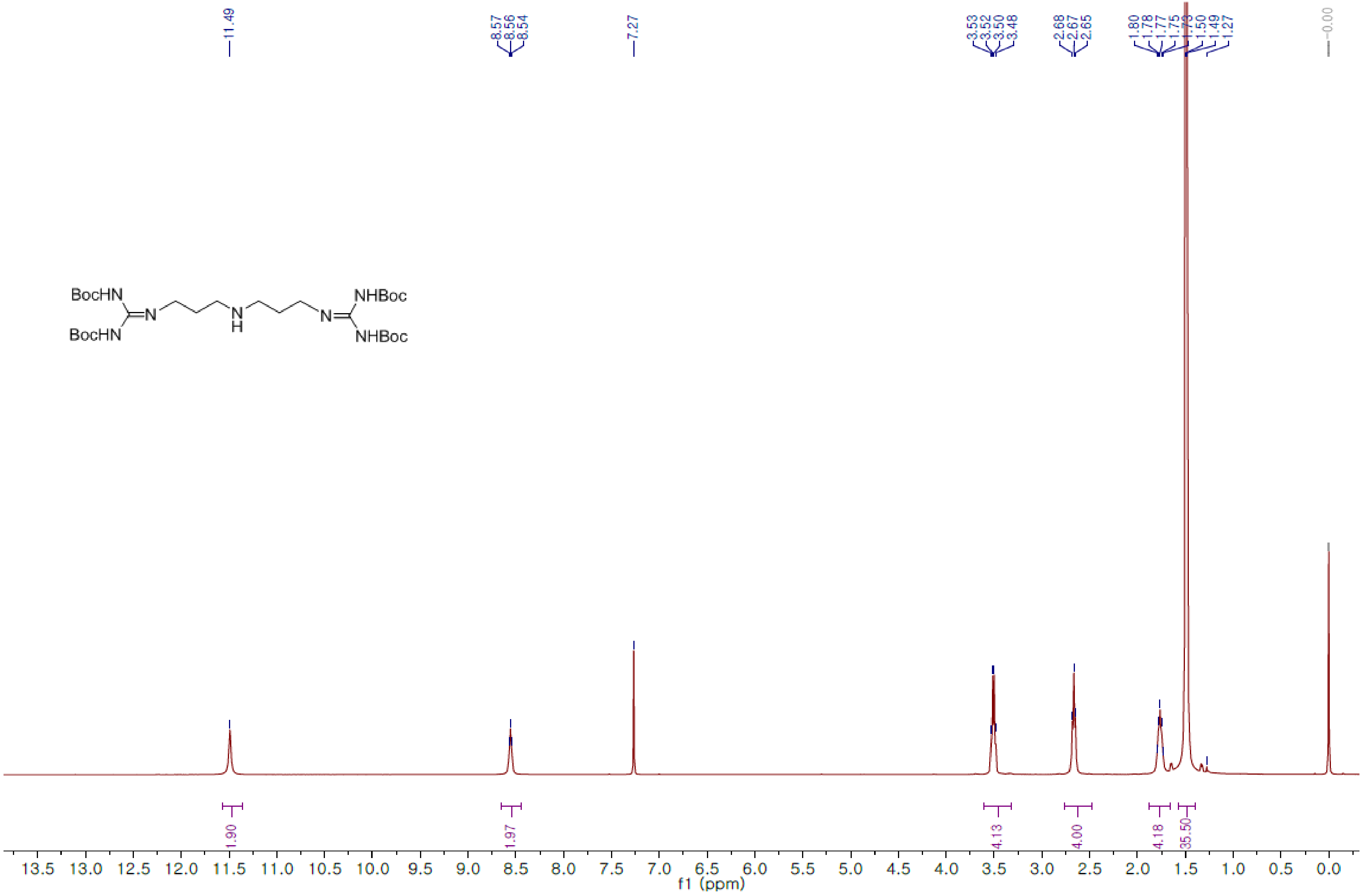

^{1}H NMR spectrum of **2** ($CDCl_3$, 400 MHz)

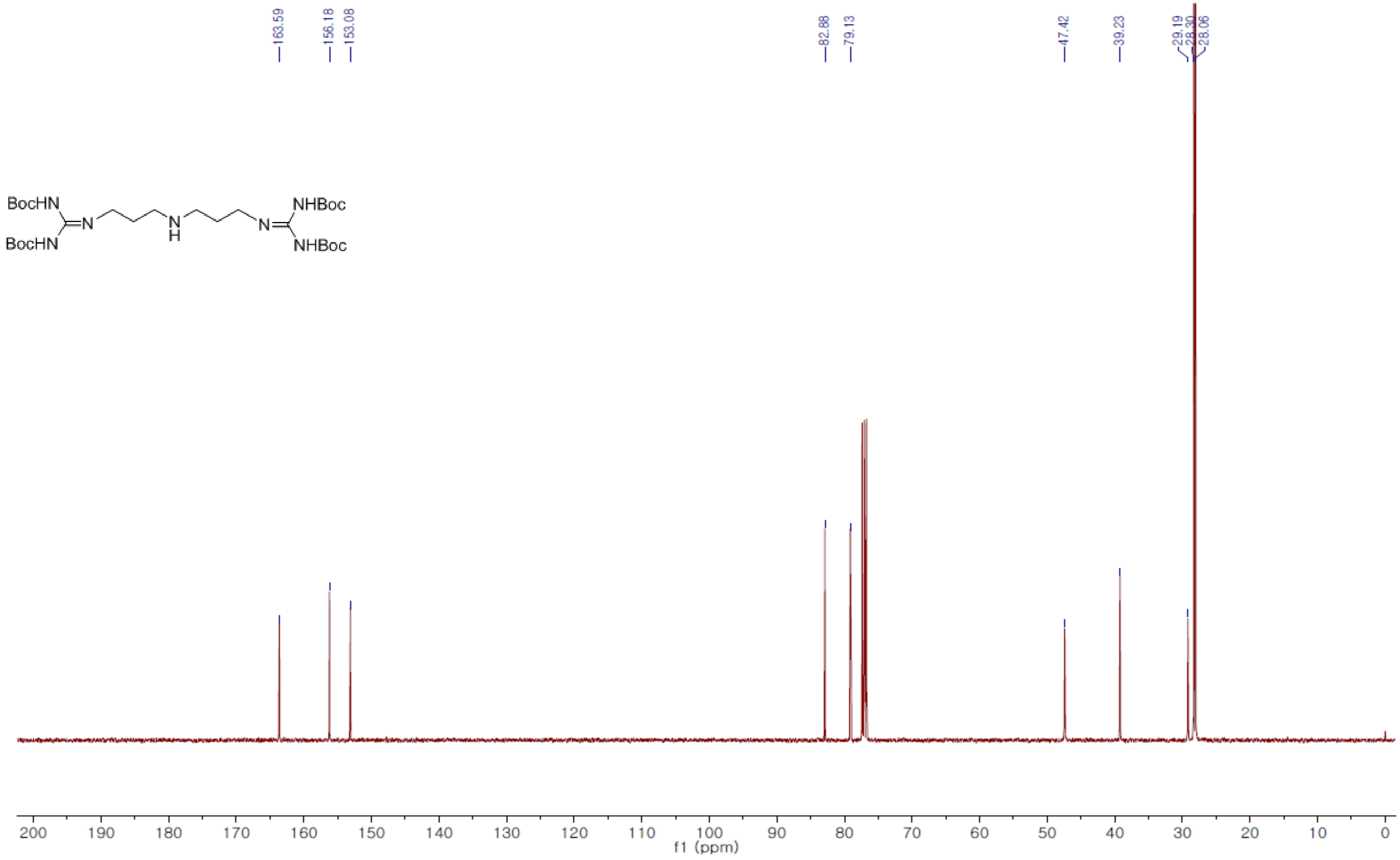

^{13}C NMR spectrum of **2** ($CDCl_3$, 101 MHz)

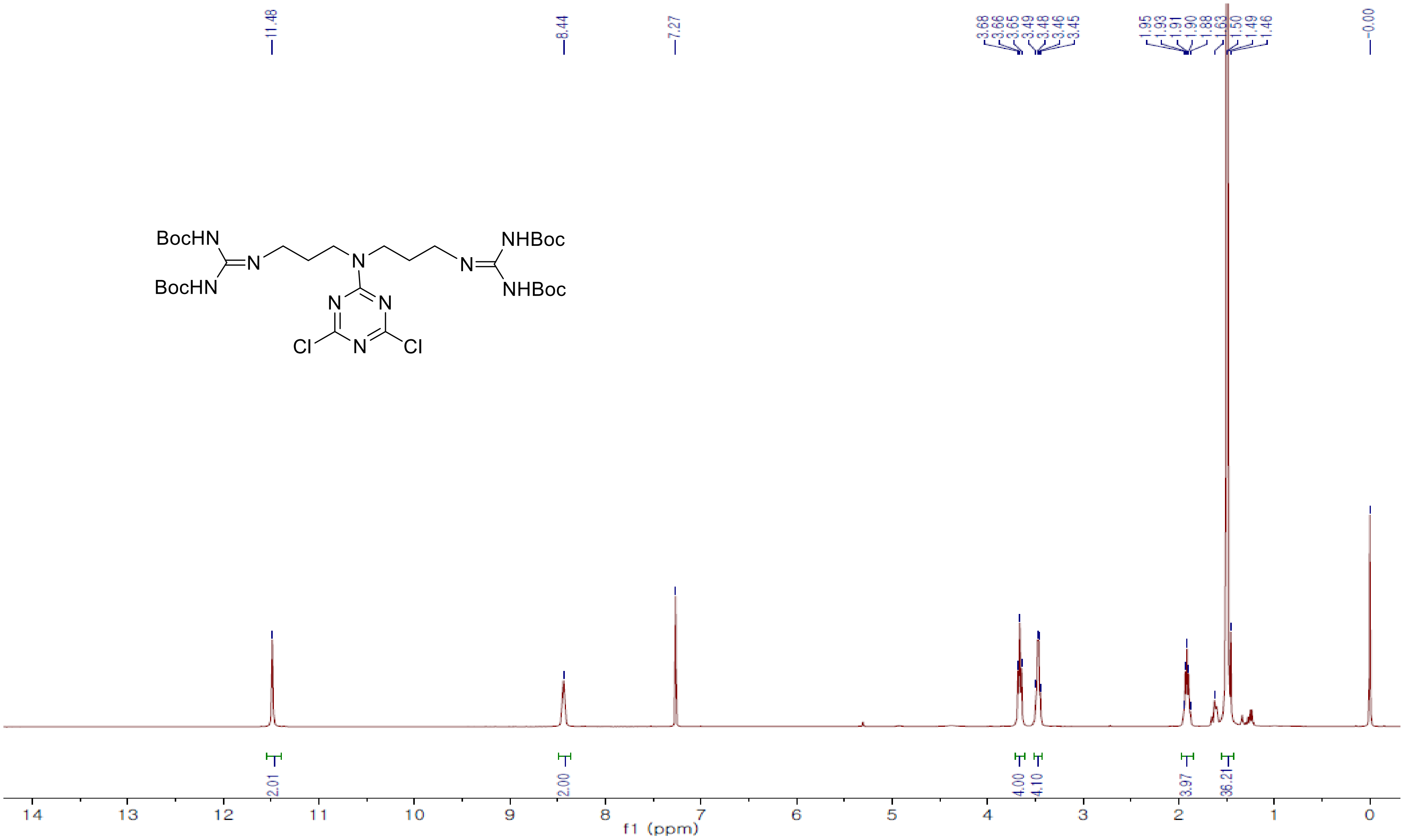

^{1}H NMR spectrum of **3** ($CDCl_3$, 400 MHz)

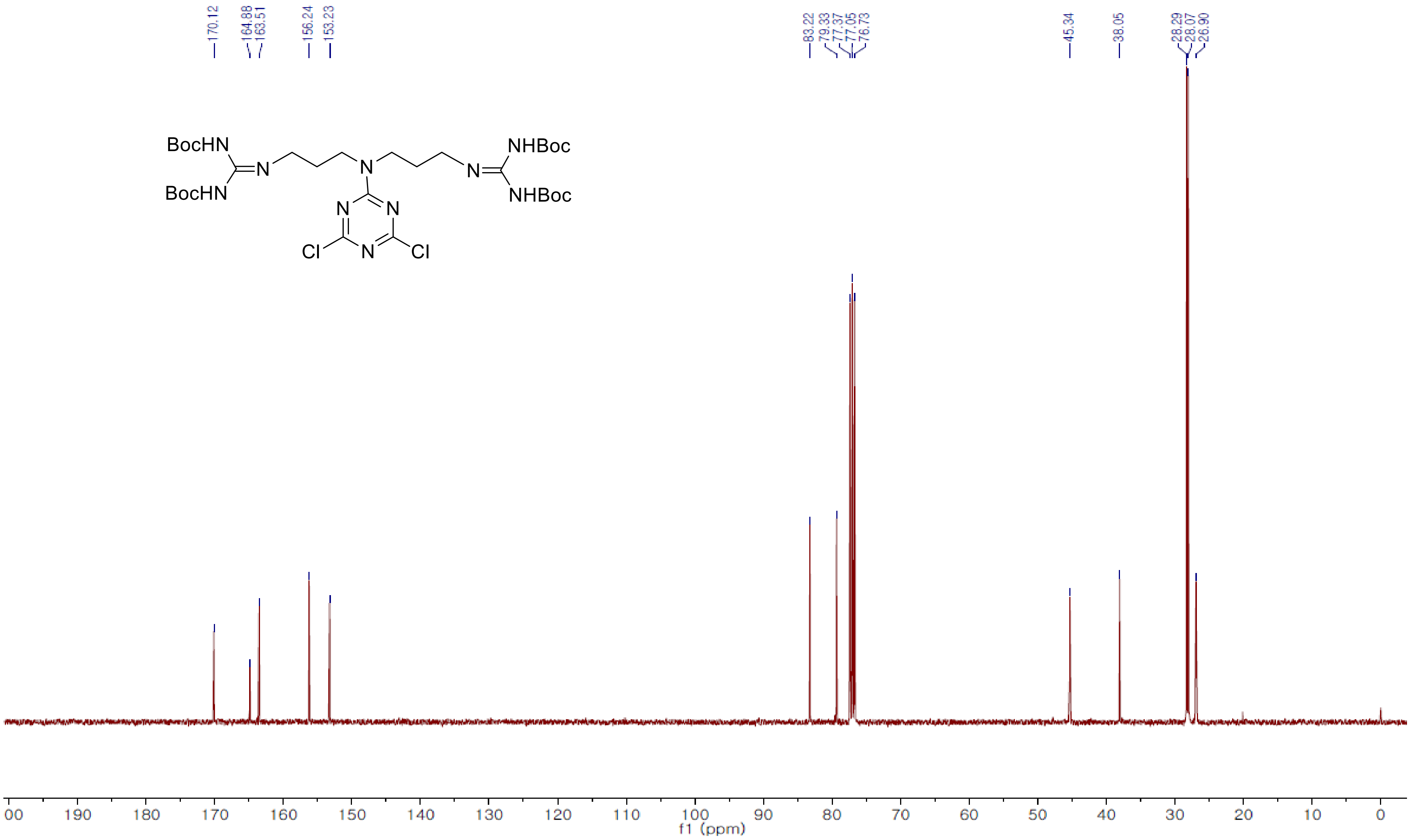

^{13}C NMR spectrum of **3** ($CDCl_3$, 101 MHz)

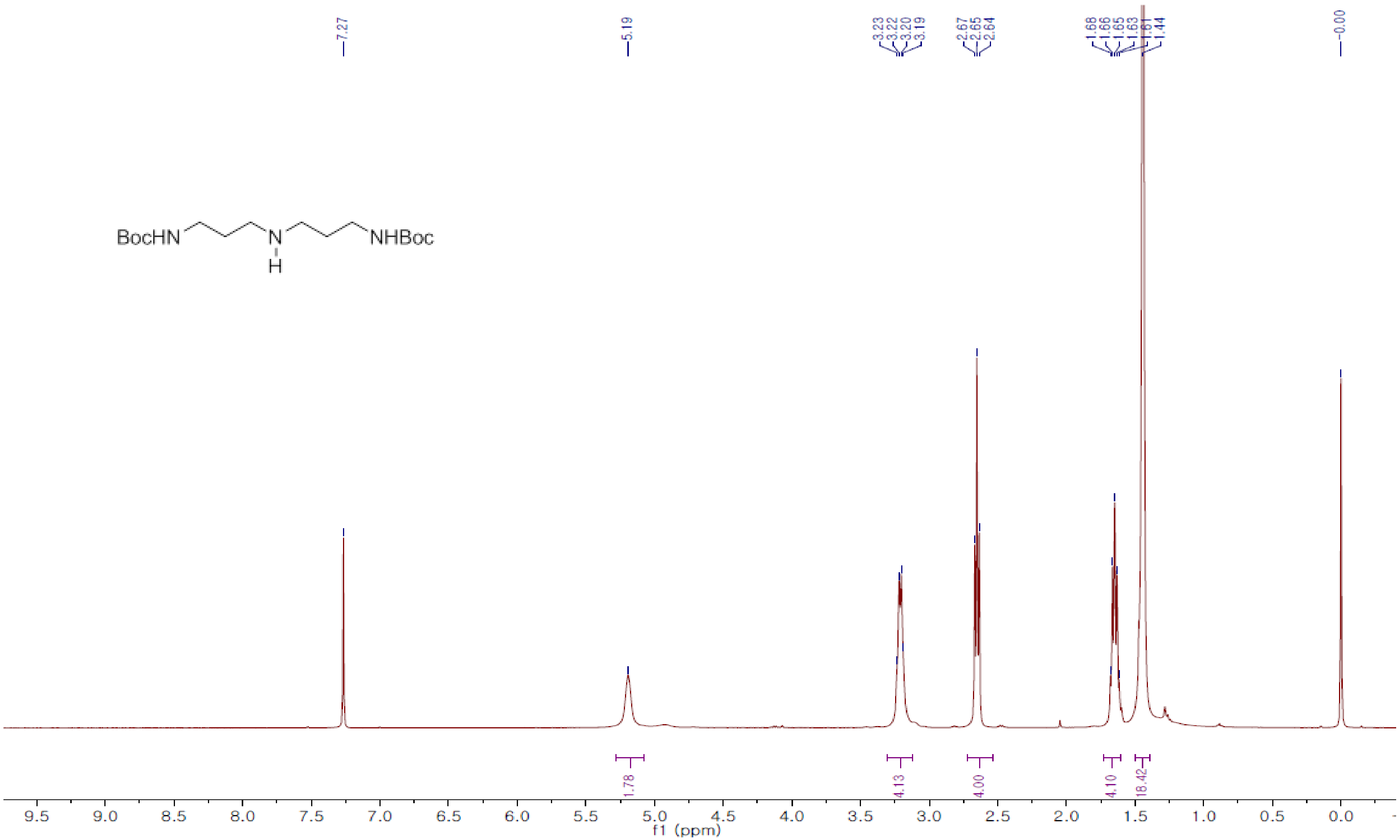

^{1}H NMR spectrum of **4** ($CDCl_3$, 400 MHz)

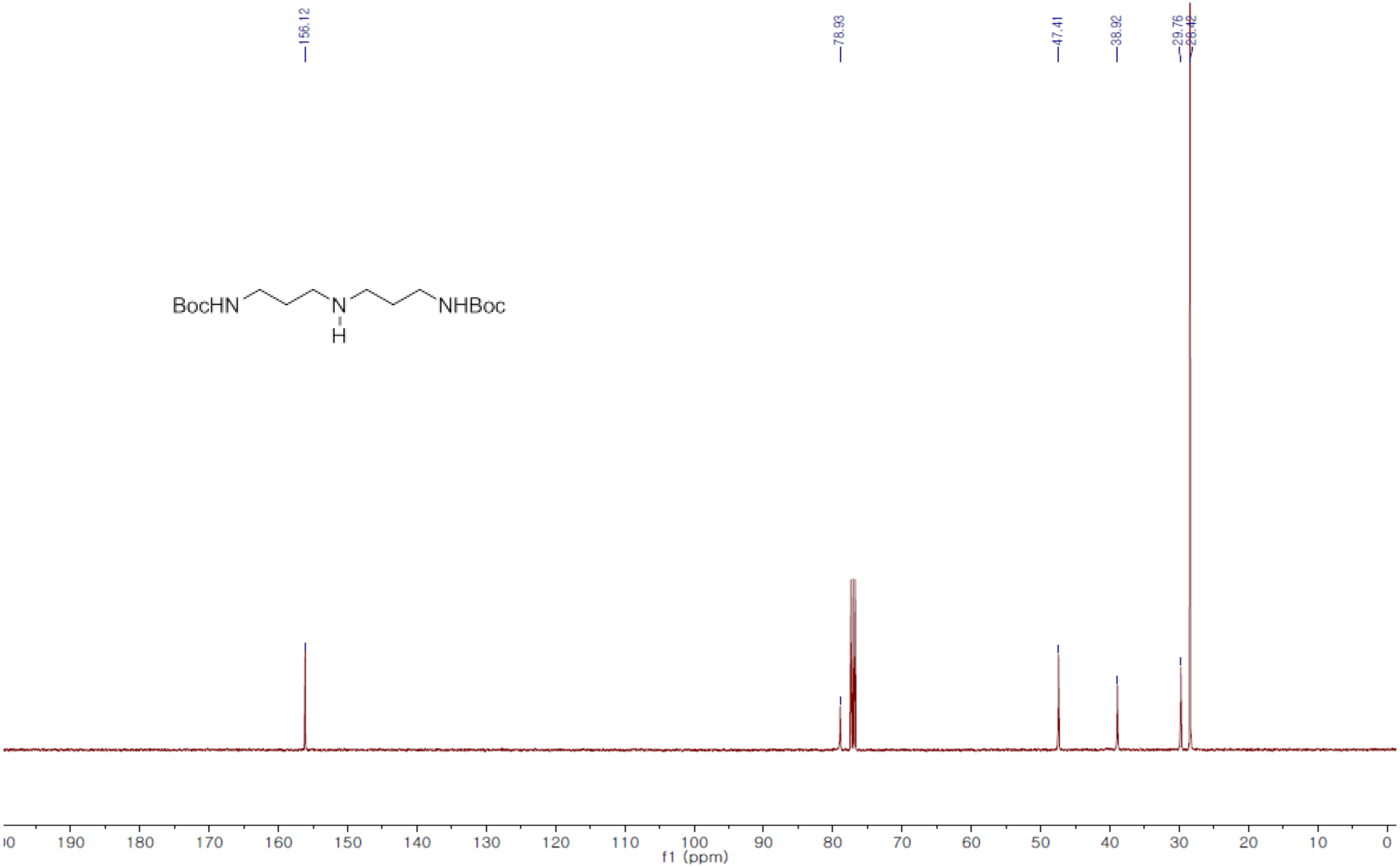

^{13}C NMR spectrum of **4** ($CDCl_3$, 101 MHz)

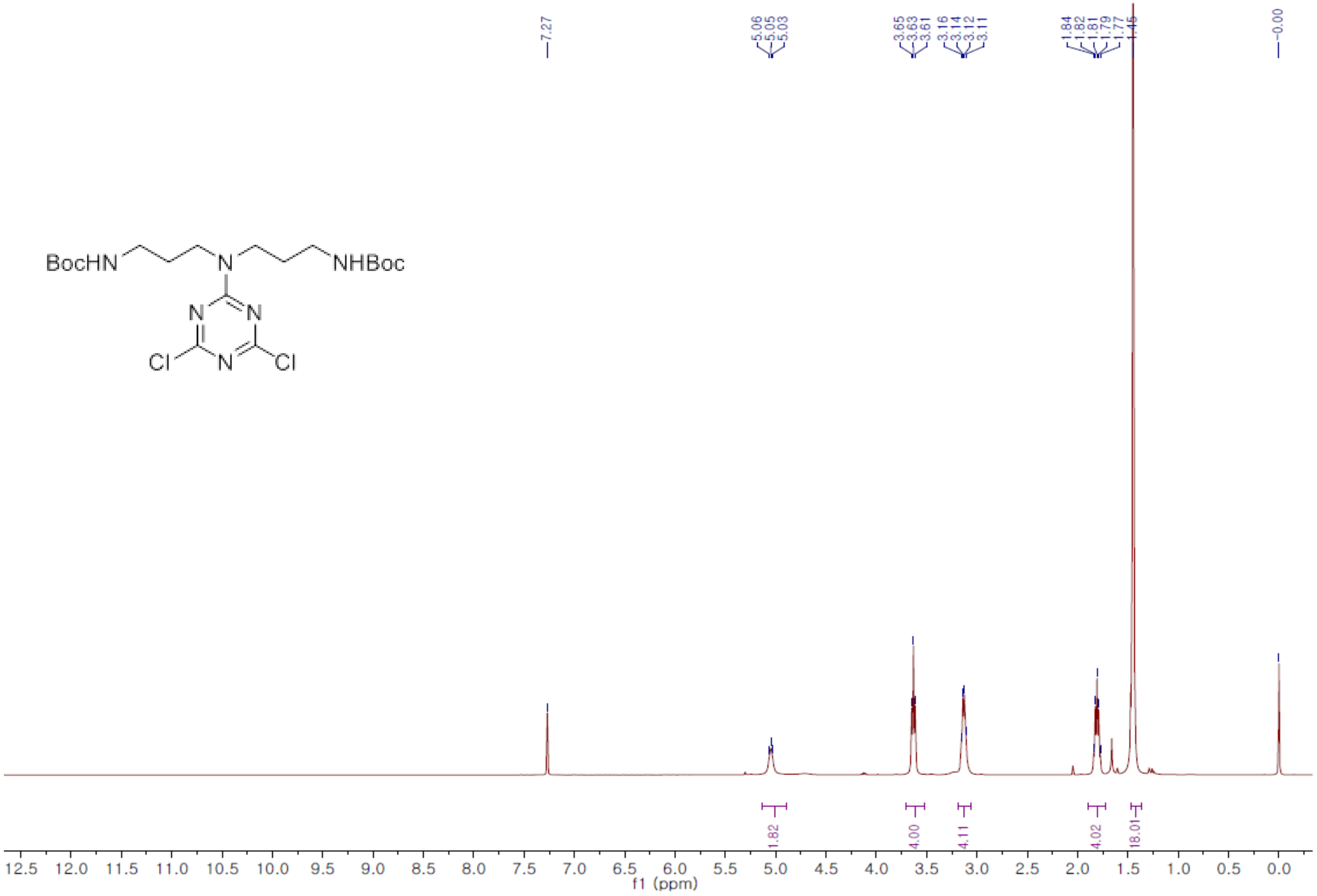

^{1}H NMR spectrum of **5** ($CDCl_3$, 400 MHz)

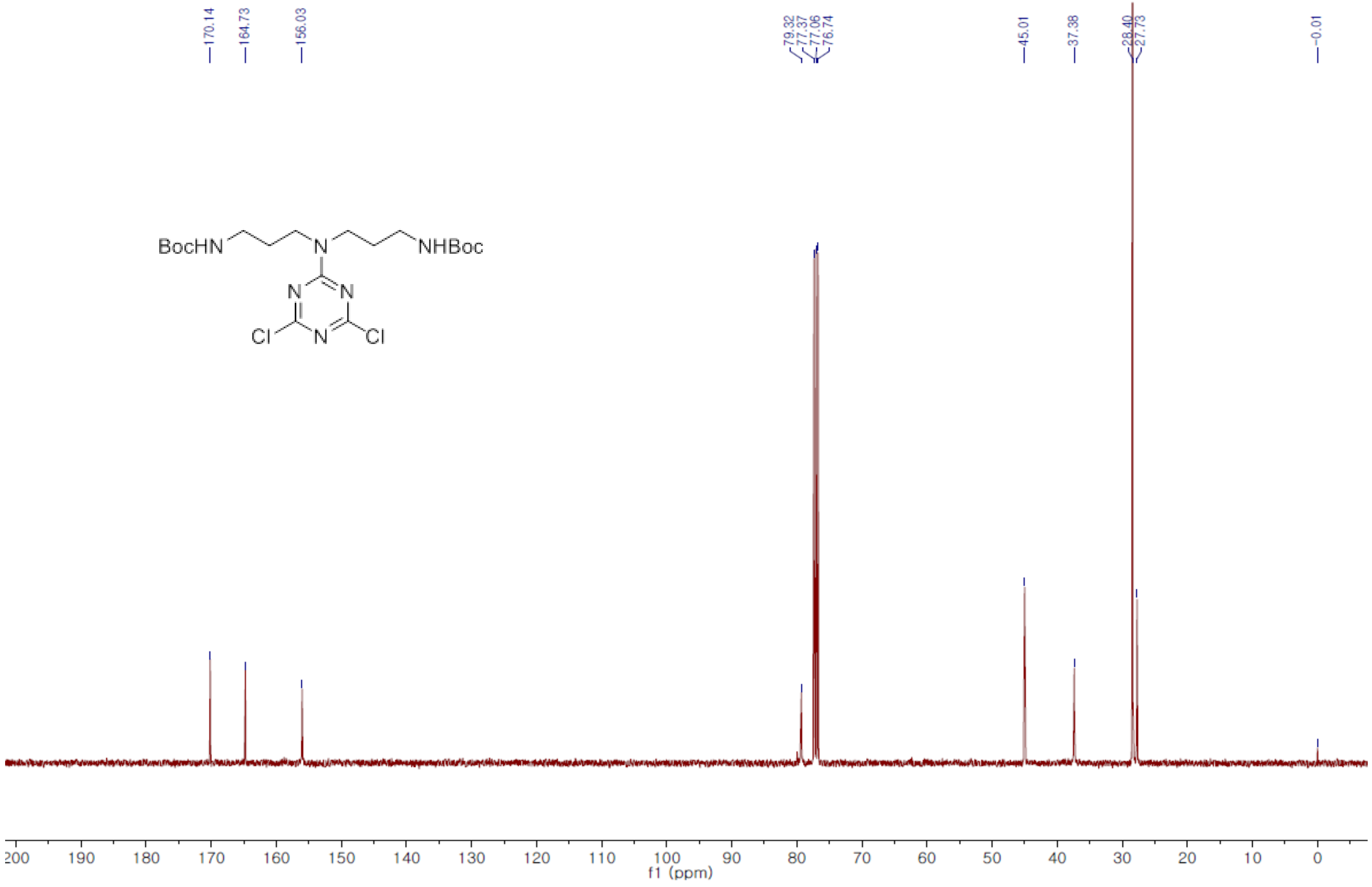

^{13}C NMR spectrum of **5** ($CDCl_3$, 101 MHz)

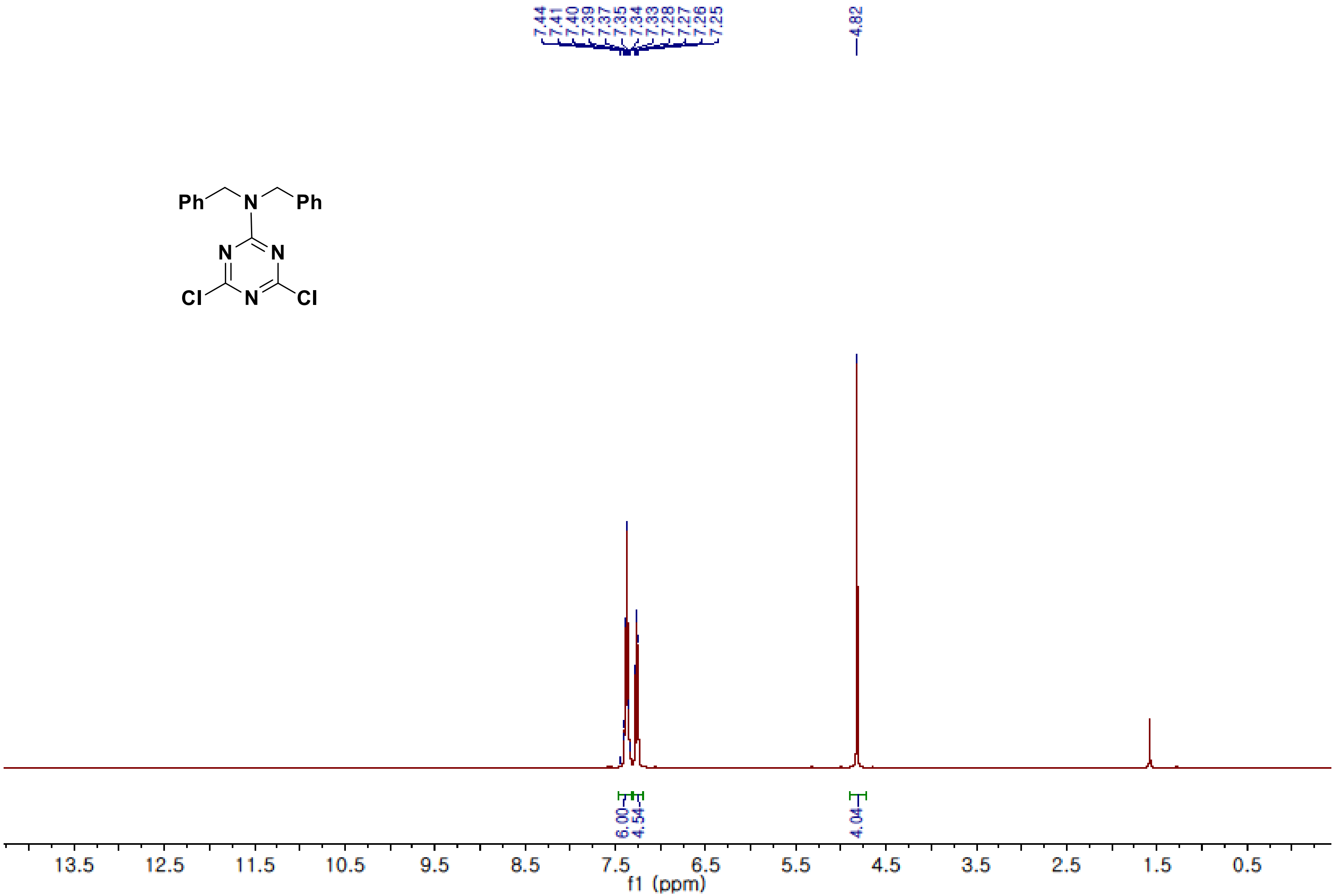

^{1}H NMR spectrum of **6a** ($CDCl_3$, 400 MHz)

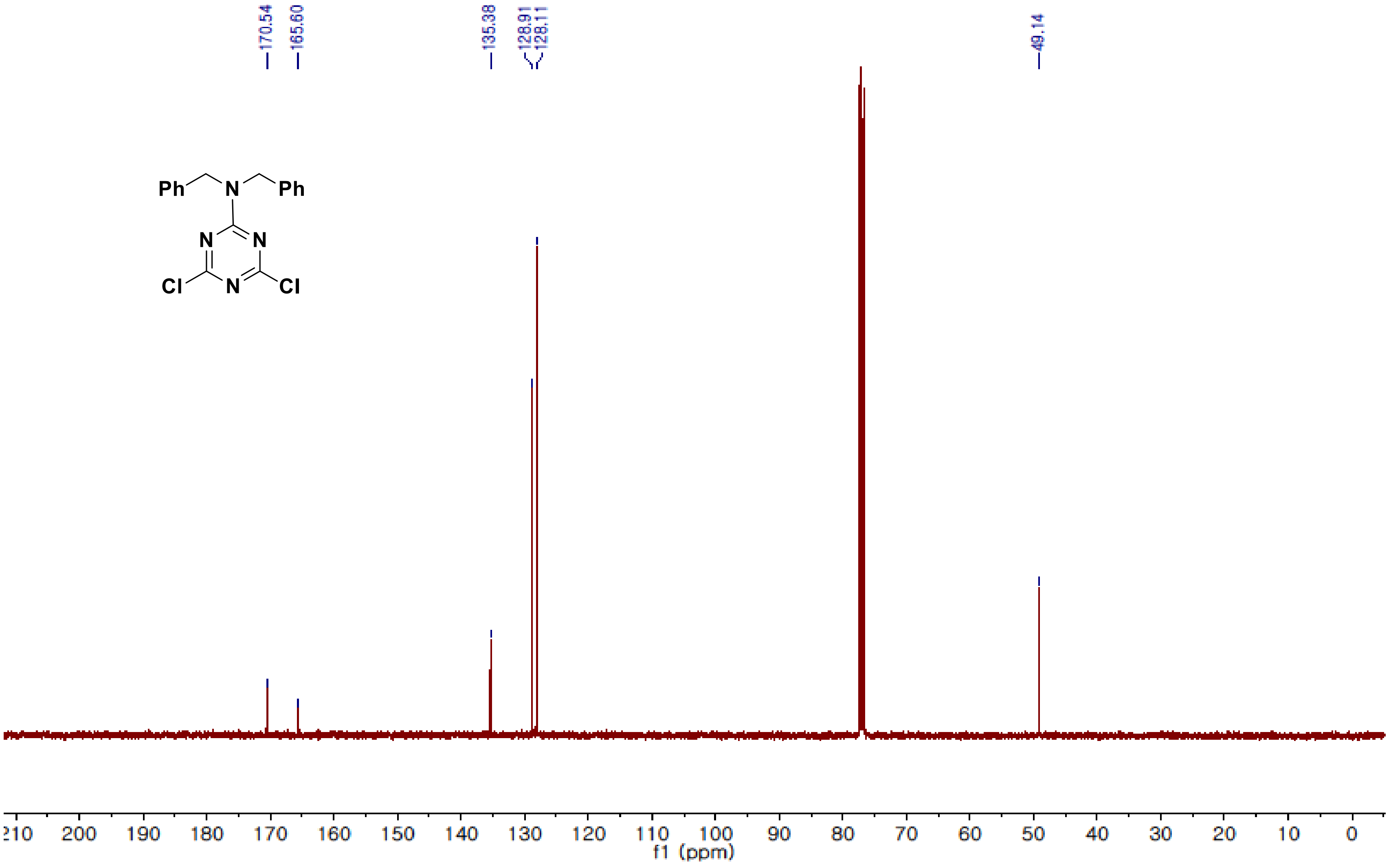

^{13}C NMR spectrum of **6a** ($CDCl_3$, 400 MHz)

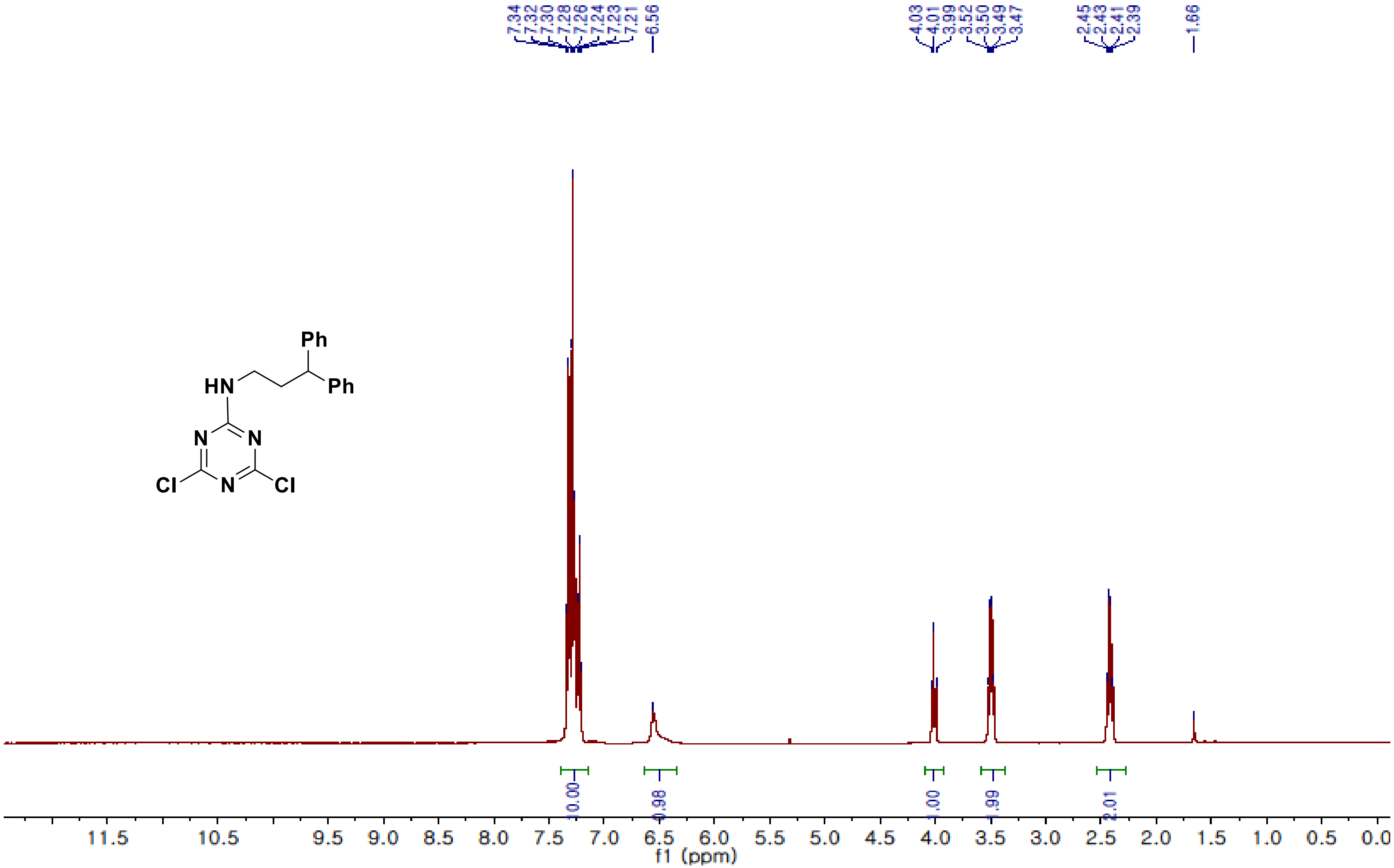

^{1}H NMR spectrum of **6b** ($CDCl_3$, 400 MHz)

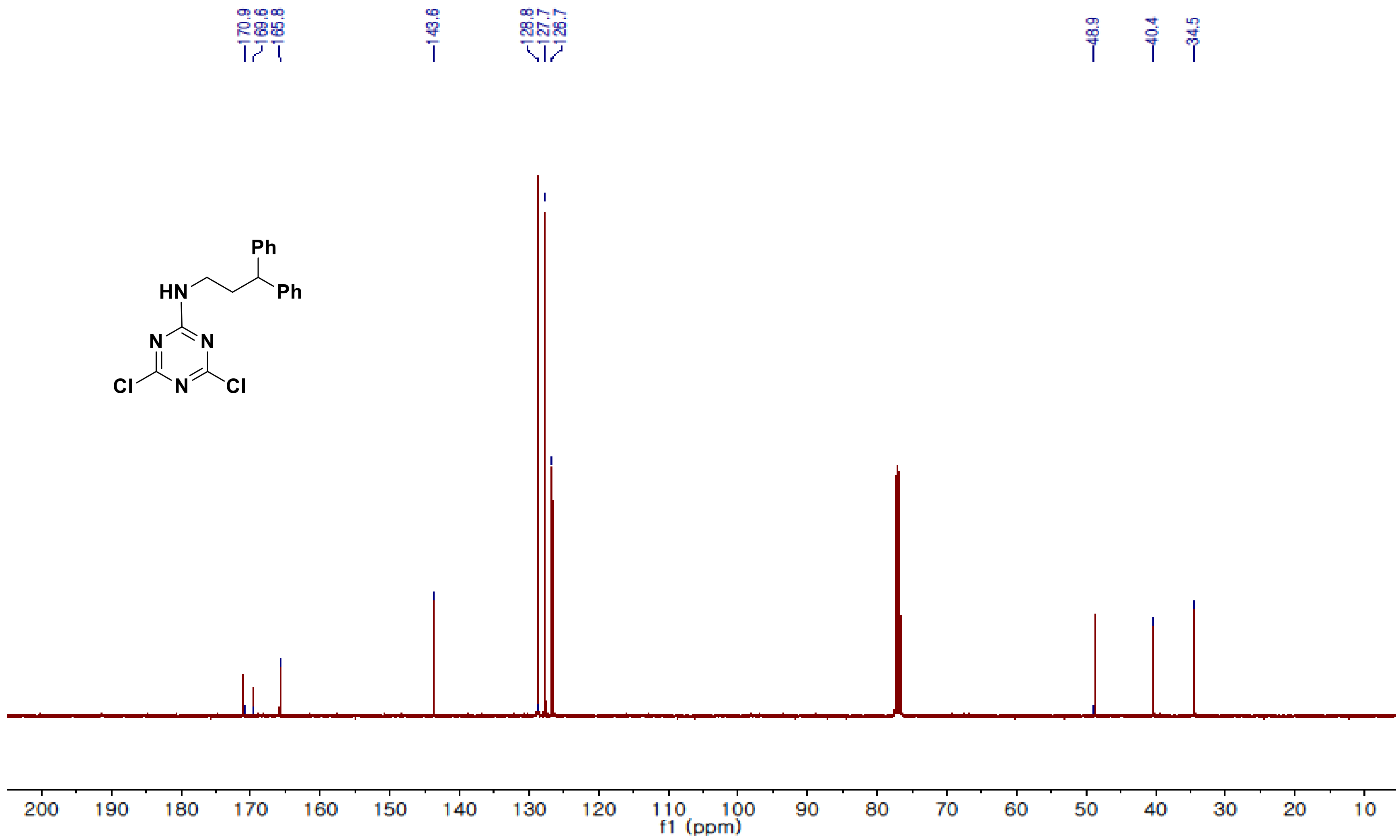

^{13}C NMR spectrum of **6b** ($CDCl_3$, 400 MHz)

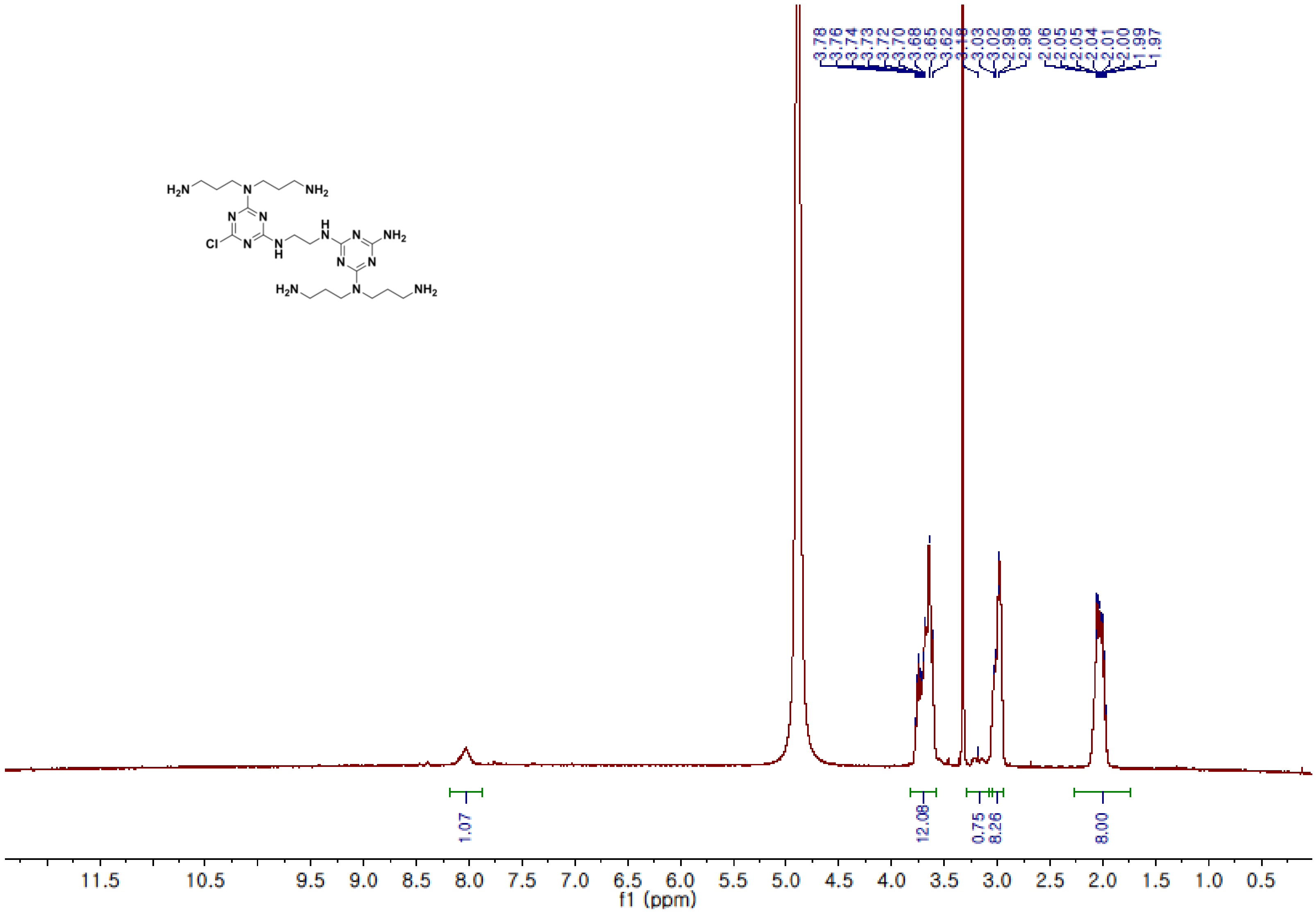

^{1}H NMR spectrum of **TZP1** (MeOD, 500 MHz)

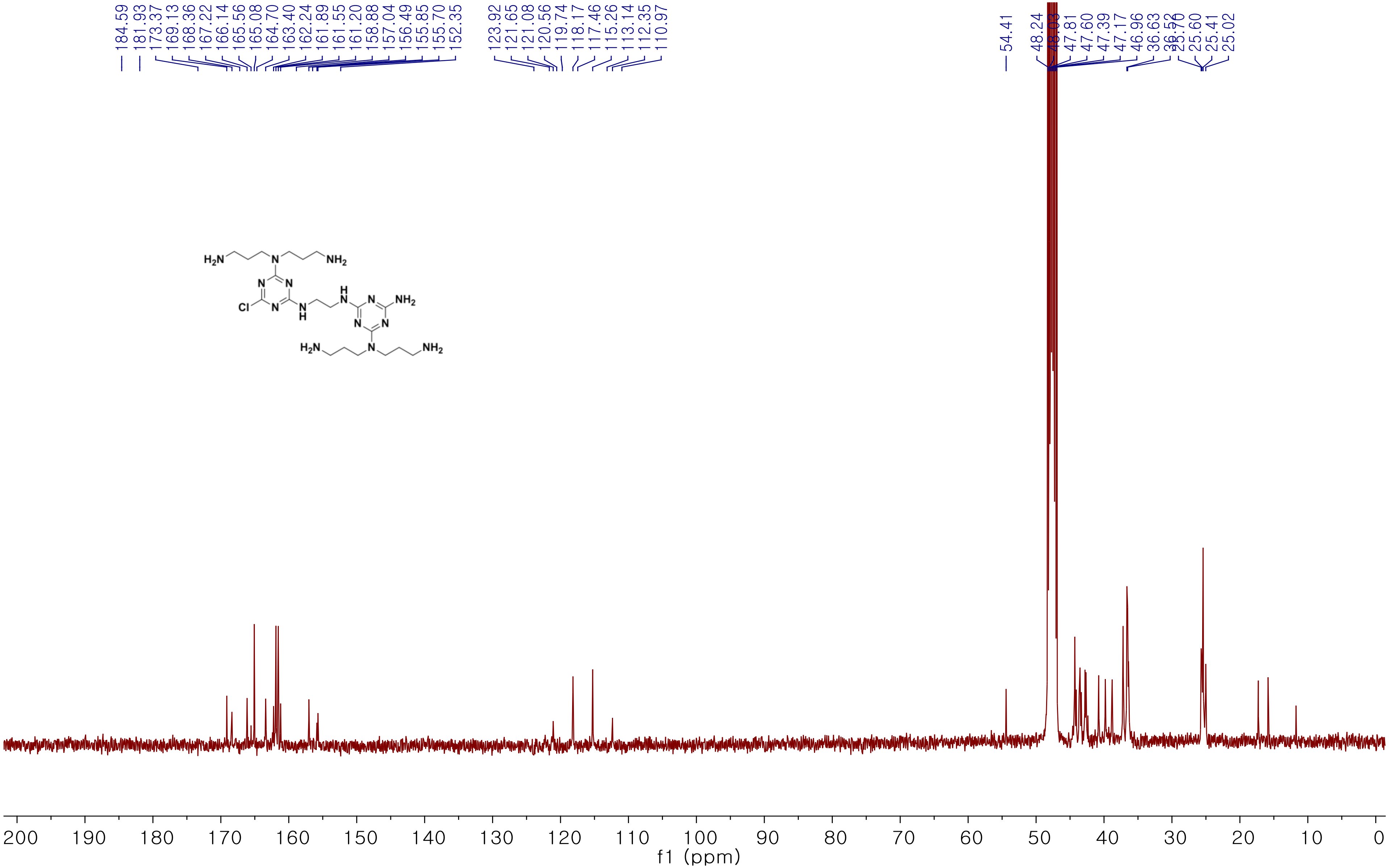

^{13}C NMR spectrum of **TZP1** (MeOD, 101 MHz)

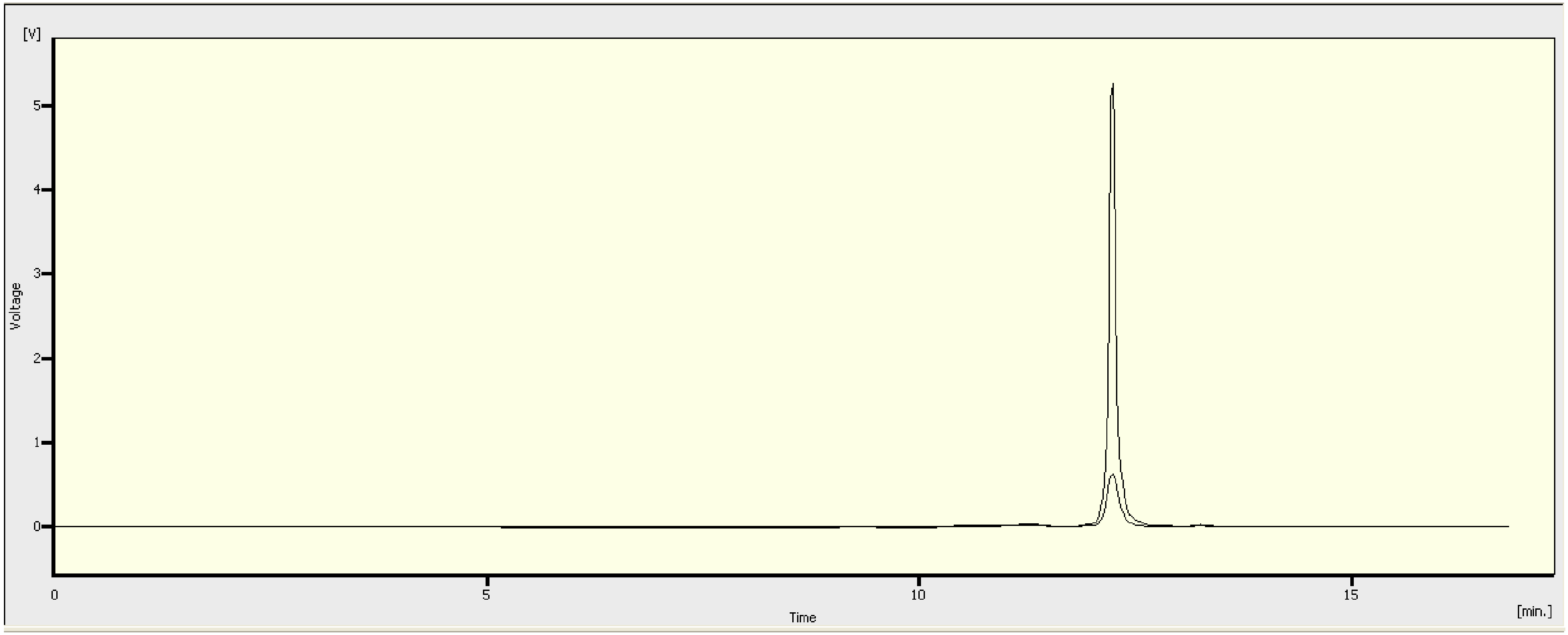

HPLC chromatogram of **TZP1**

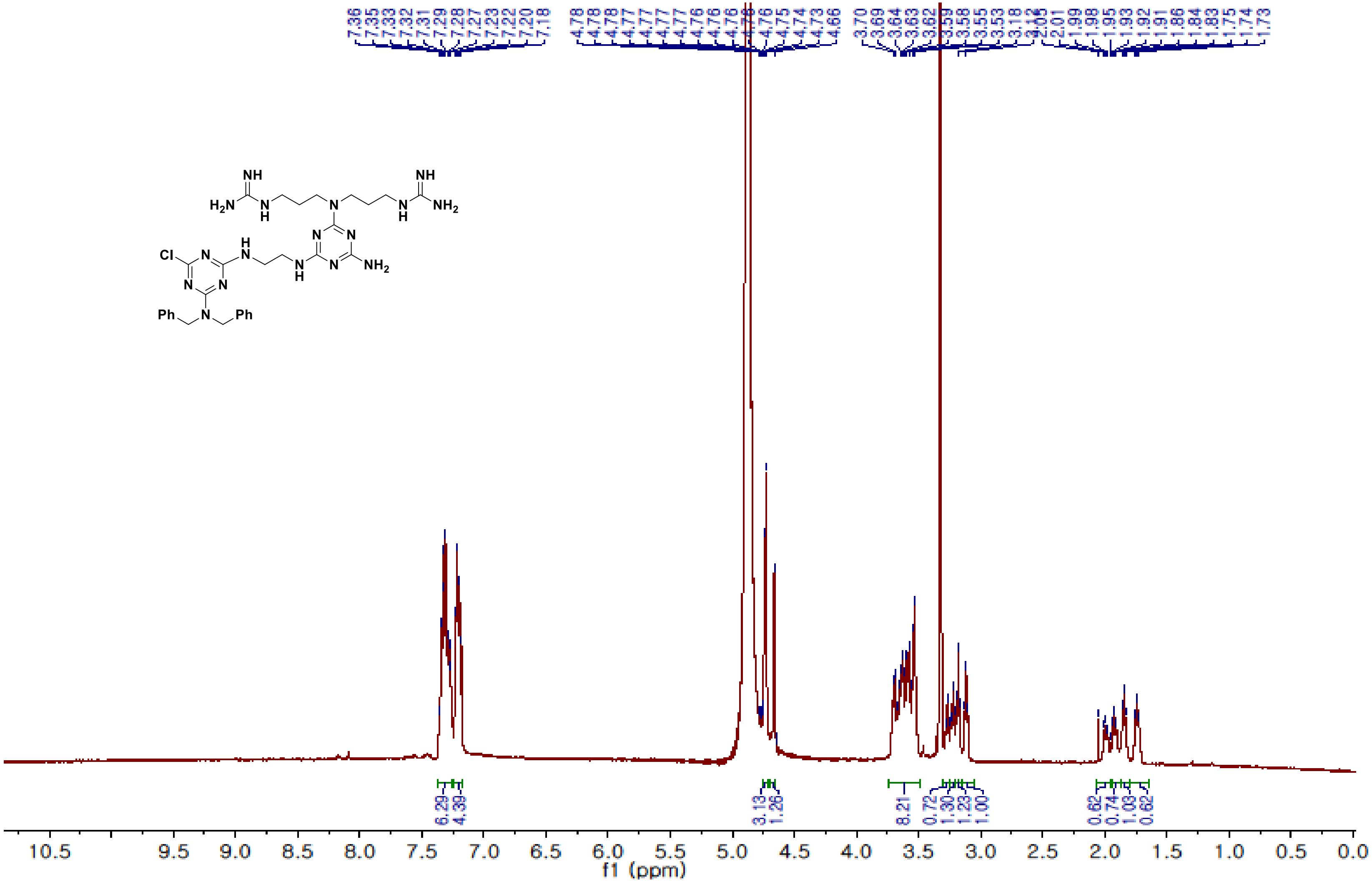

^{1}H NMR spectrum of **TZP2** (MeOD, 500 MHz)

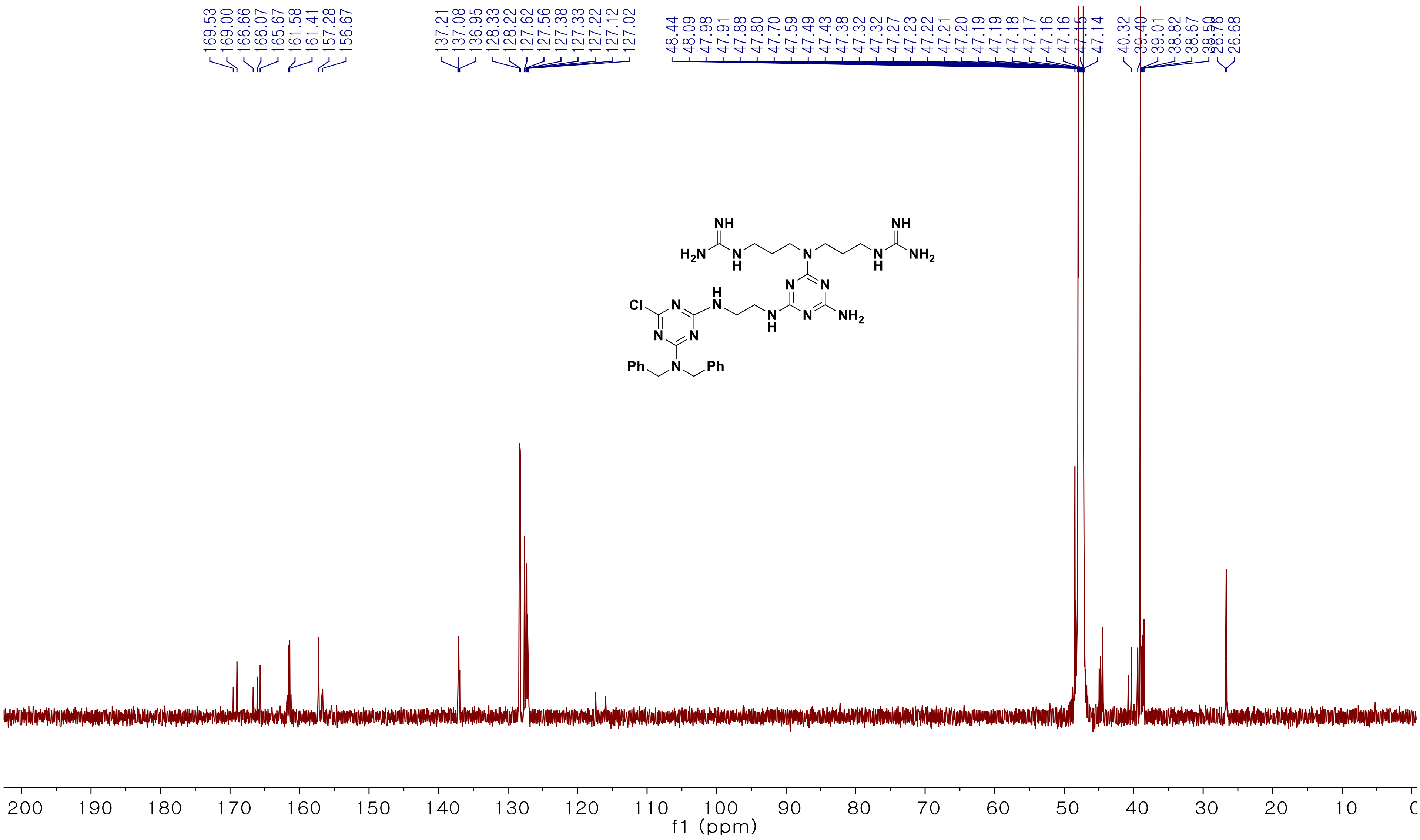

^{13}C NMR spectrum of **TZP2** (MeOD, 201 MHz)

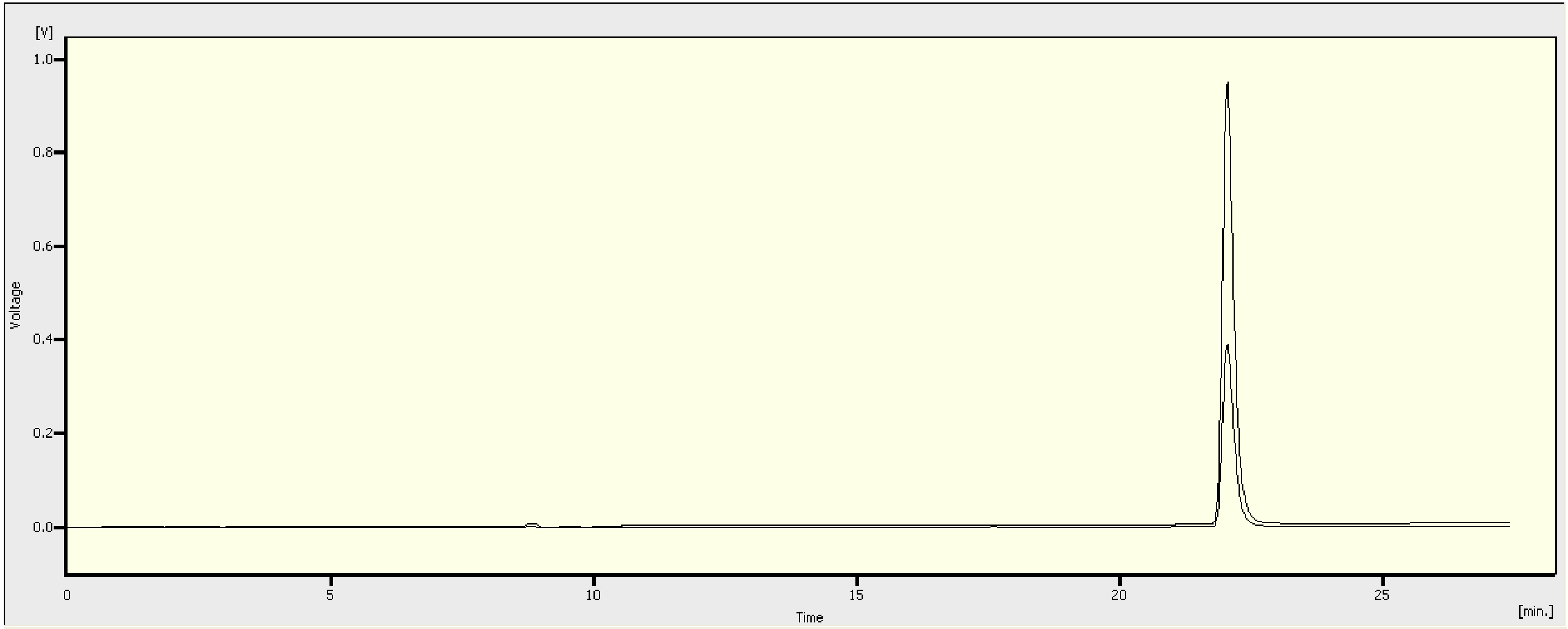

HPLC chromatogram of **TZP2**

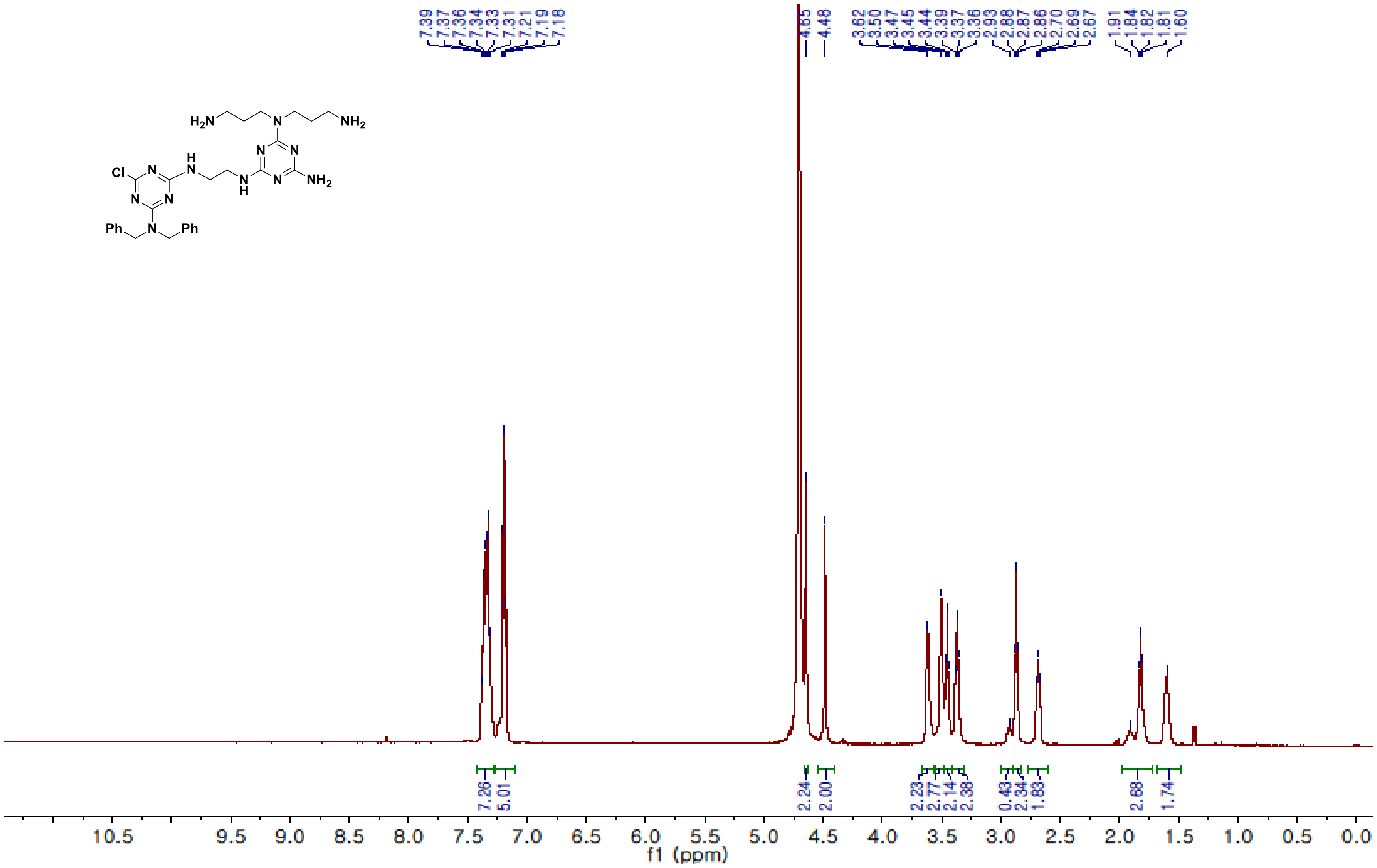

^{1}H NMR spectrum of **TZP3** (MeOD, 500 MHz)

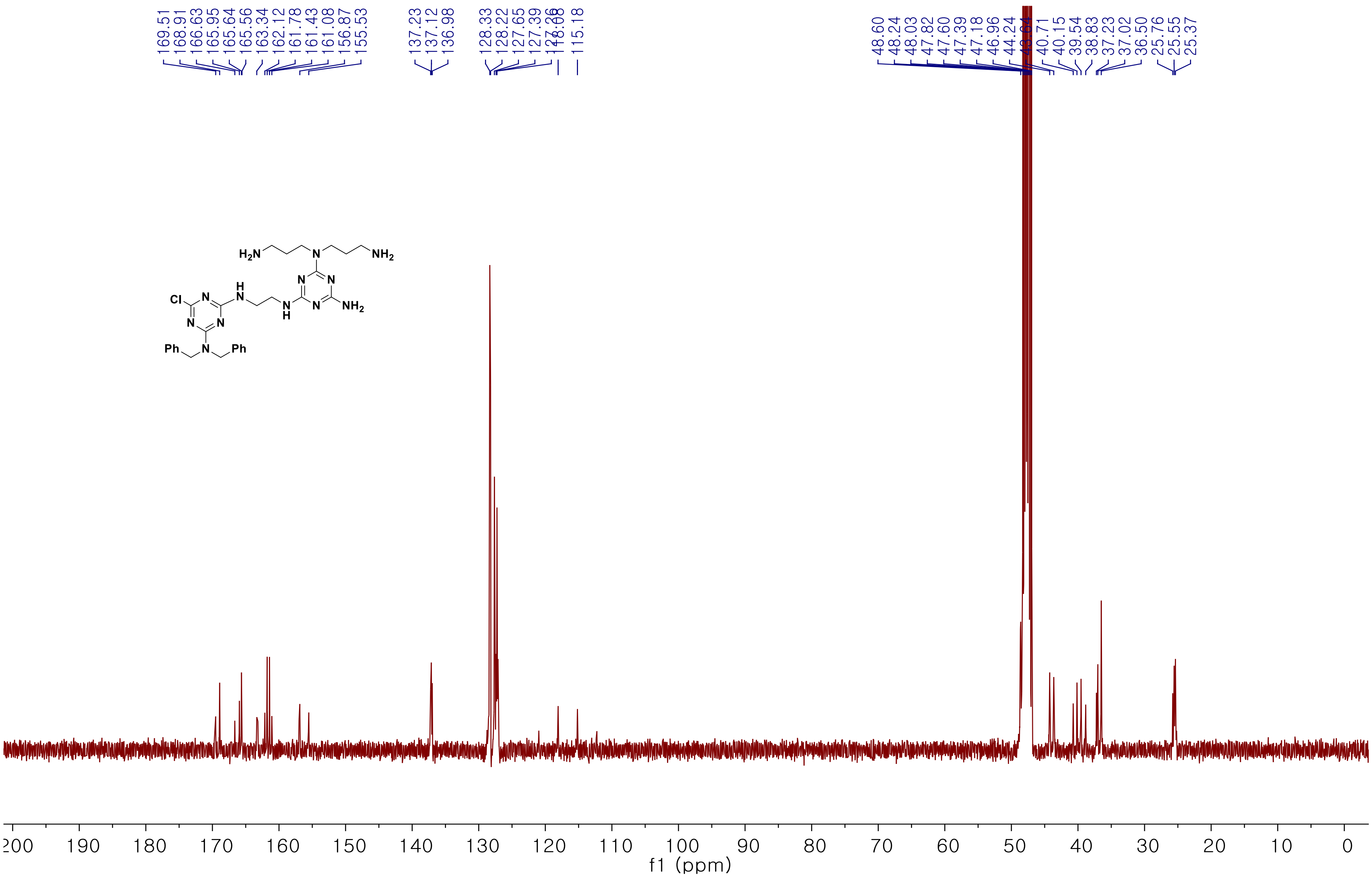

^{1}H NMR spectrum of **TZP3** (MeOD, 101 MHz)

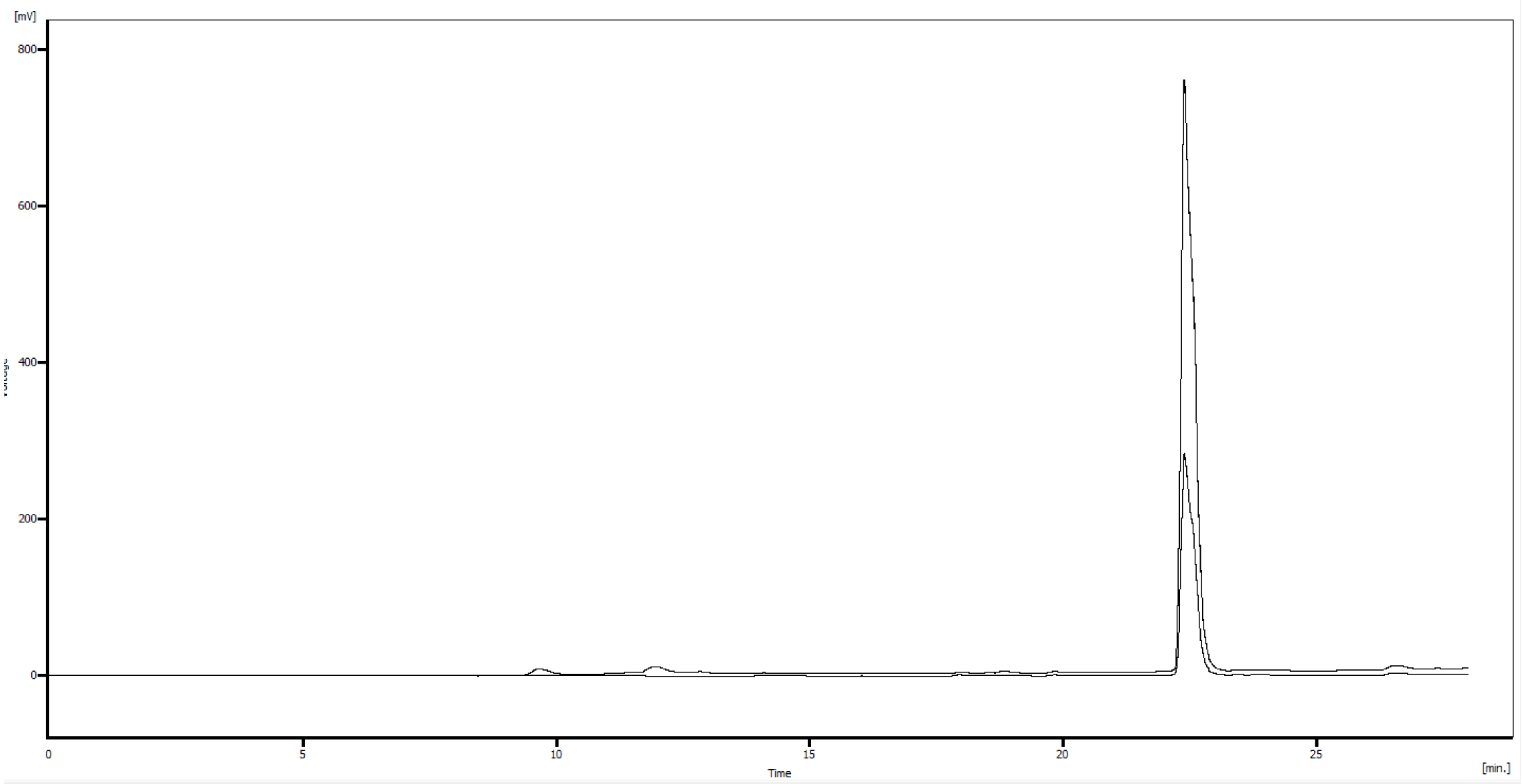

HPLC chromatogram of **TZP-3**

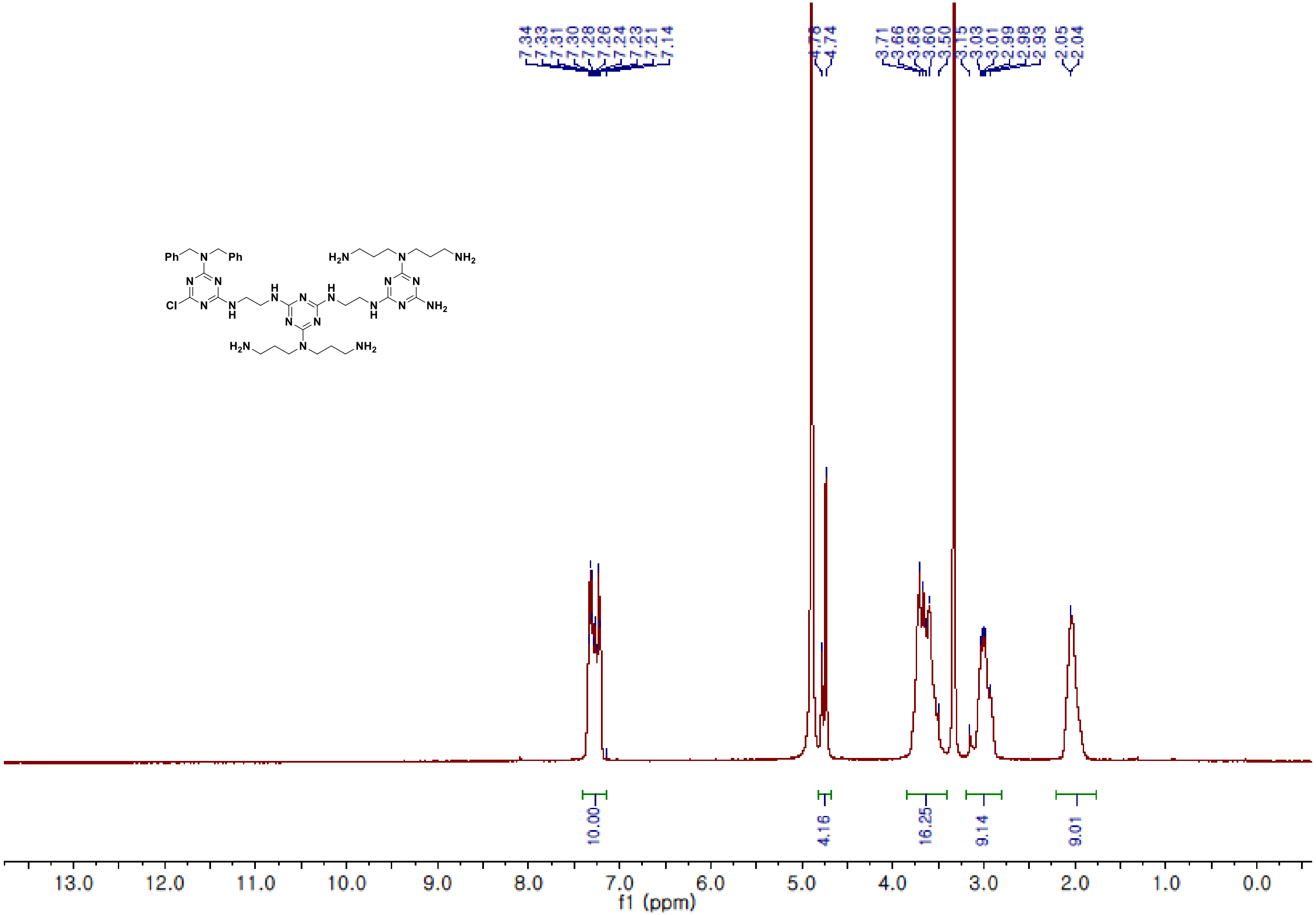

^{1}H NMR spectrum of **TZP4** (MeOD, 400 MHz)

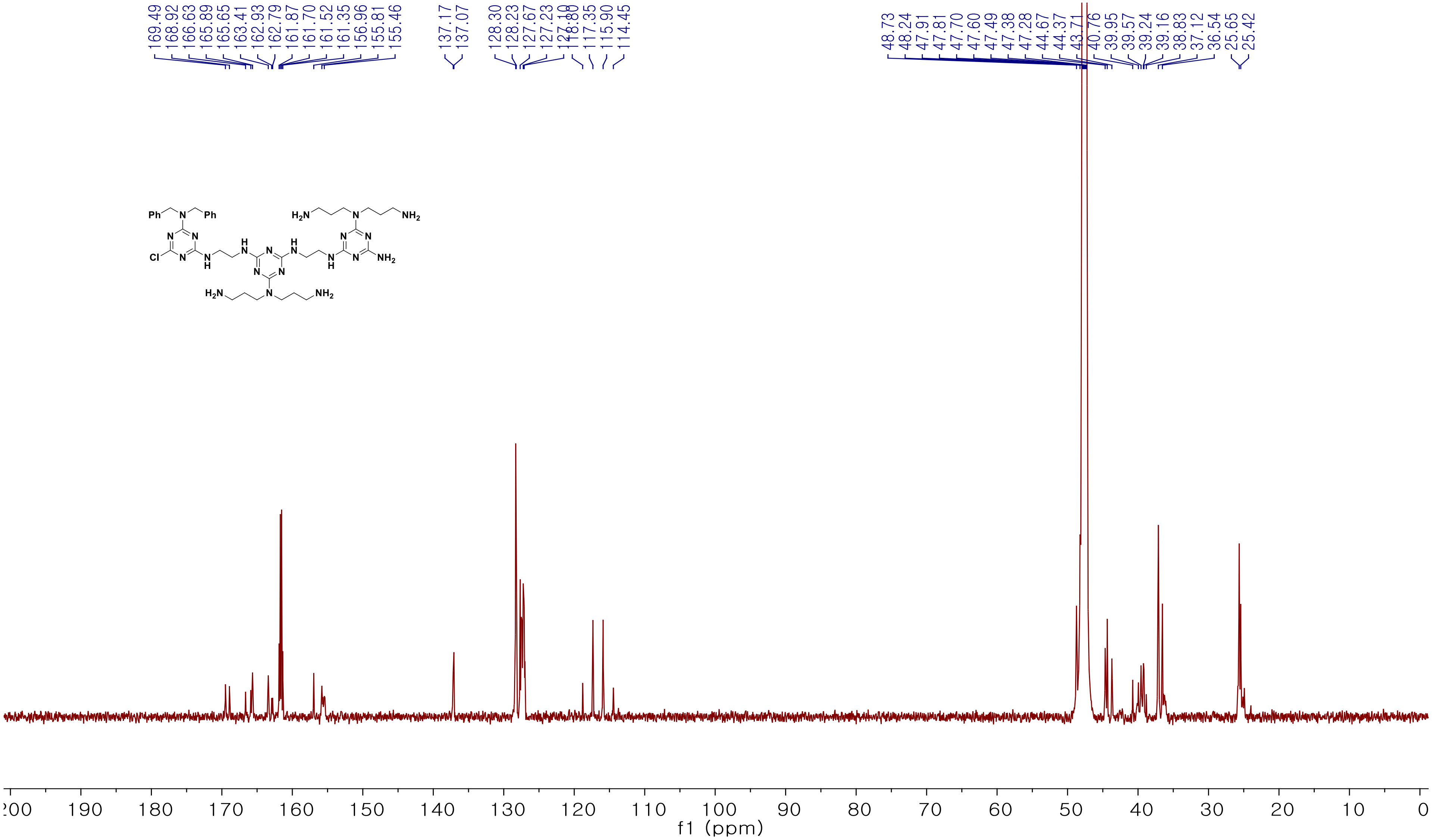

^{13}C NMR spectrum of **TZP4** (MeOD, 201 MHz)

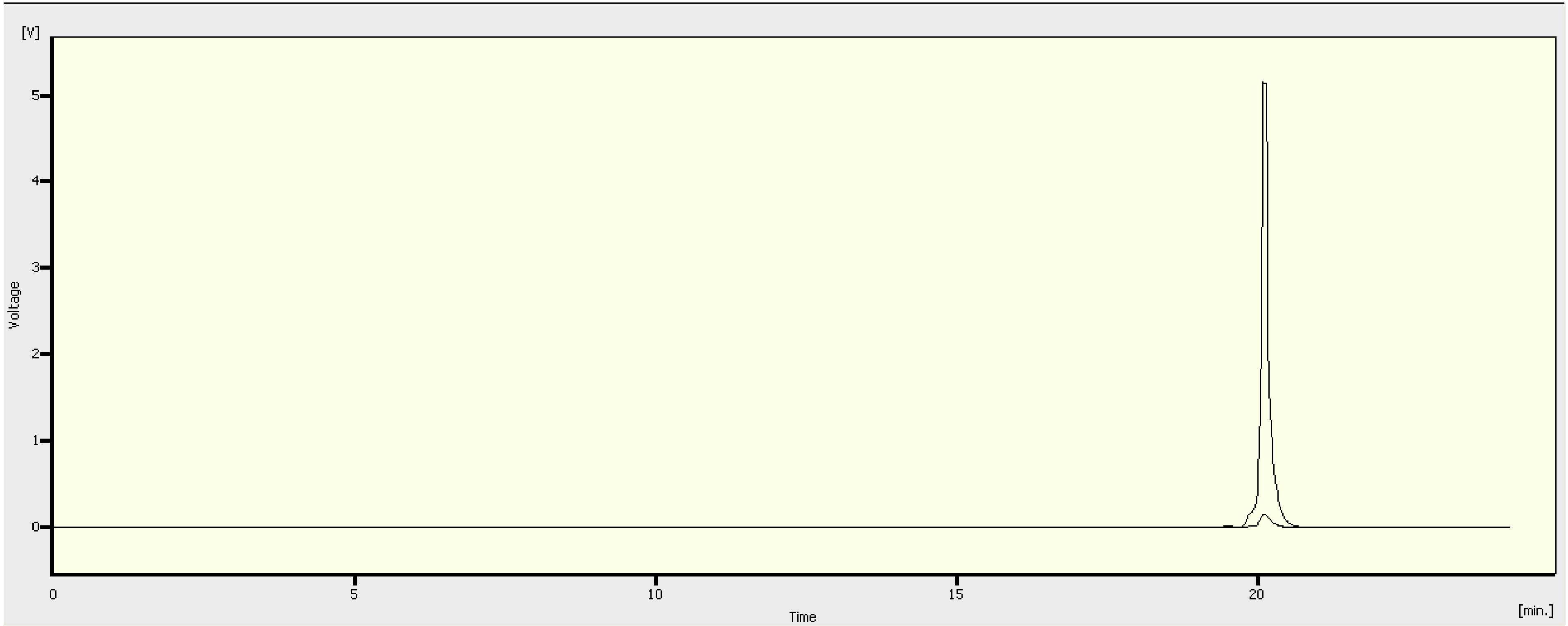

HPLC chromatogram of **TZP4**

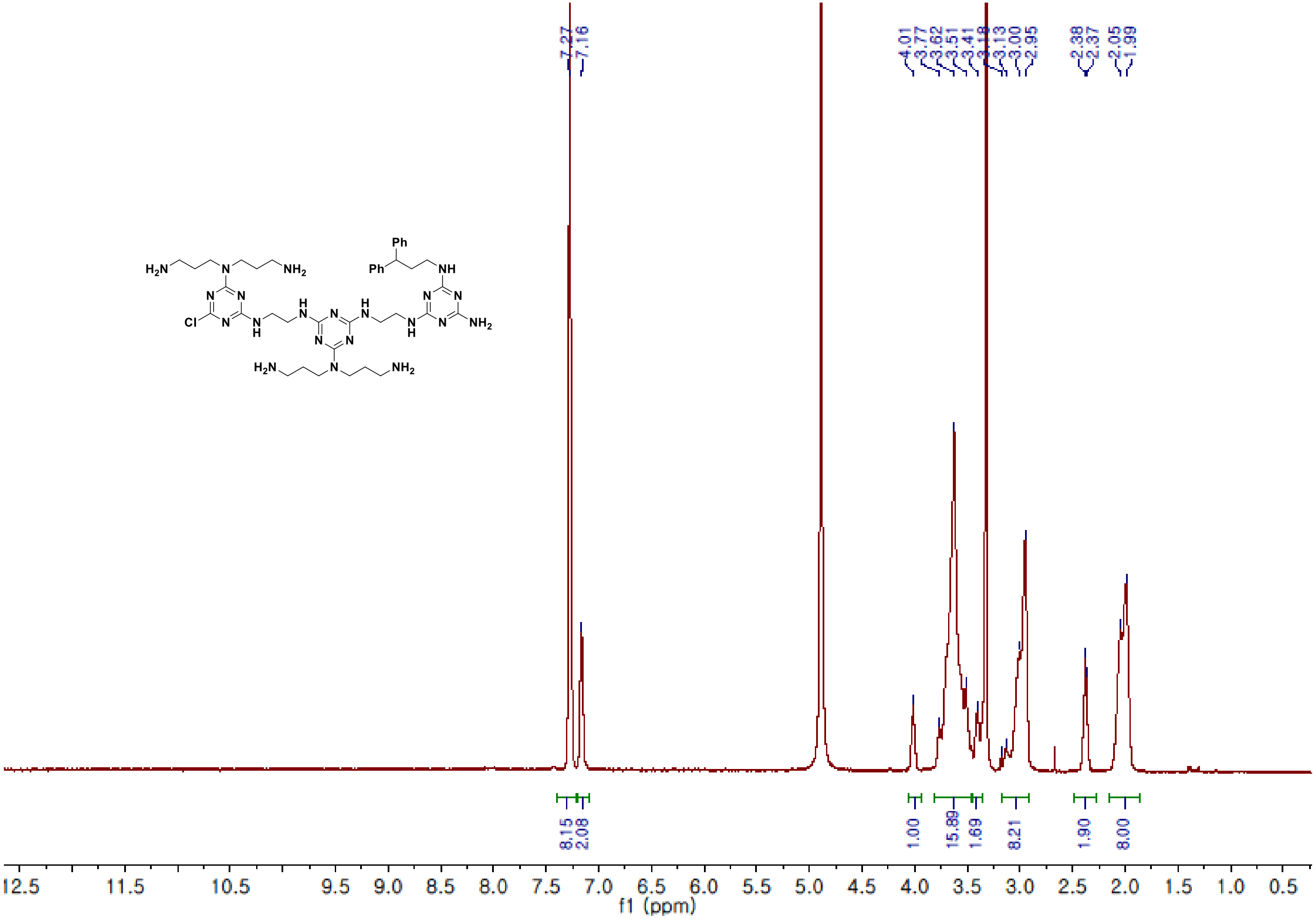

^{1}H NMR spectrum of **TZP5** (MeOD, 500 MHz)

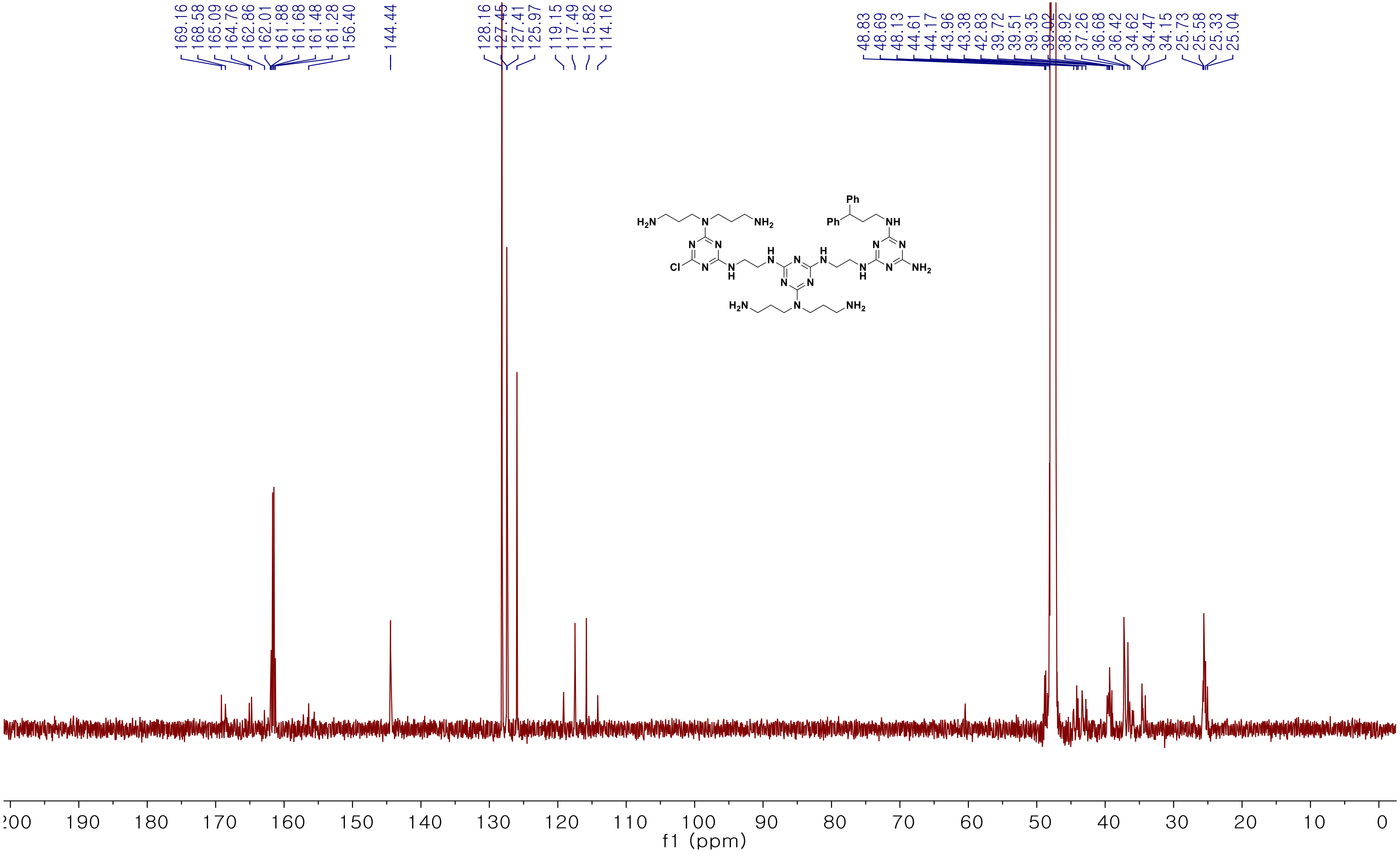

^{13}C NMR spectrum of **TZP5** (MeOD, 176 MHz)

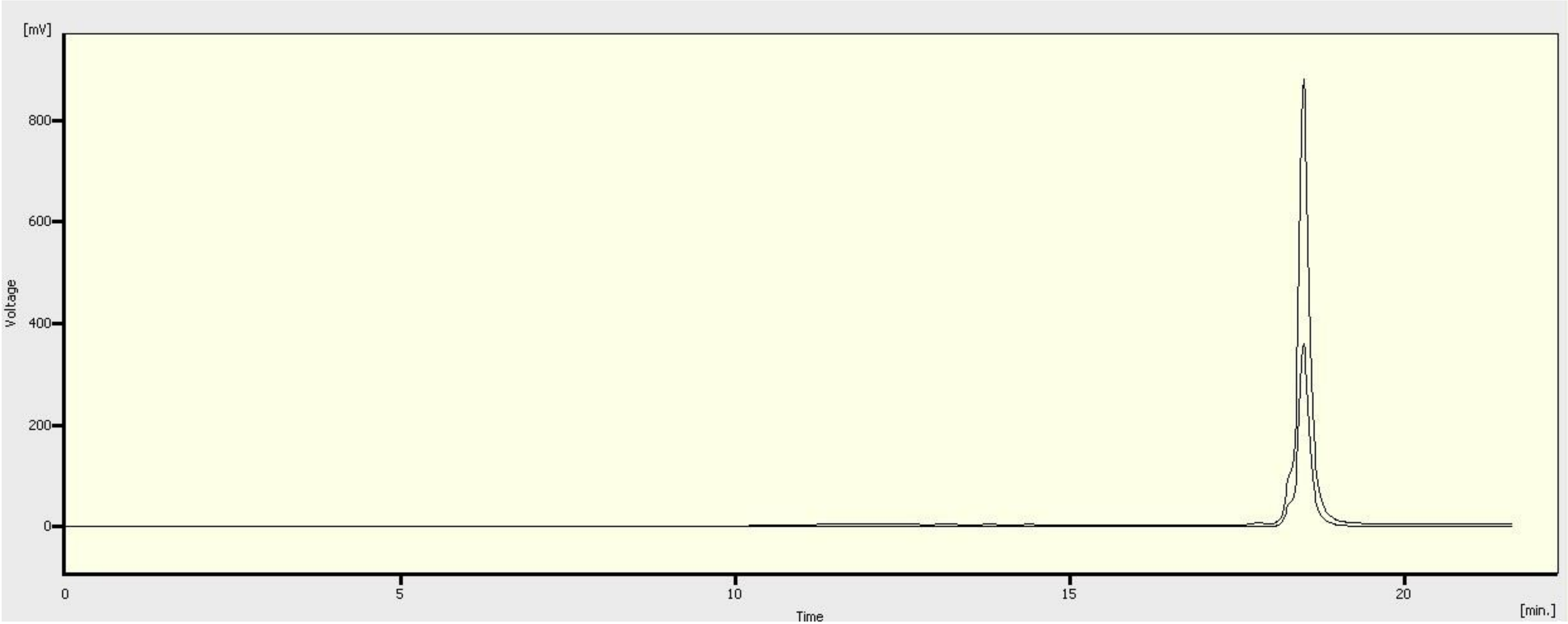

HPLC chromatogram of **TZP5**

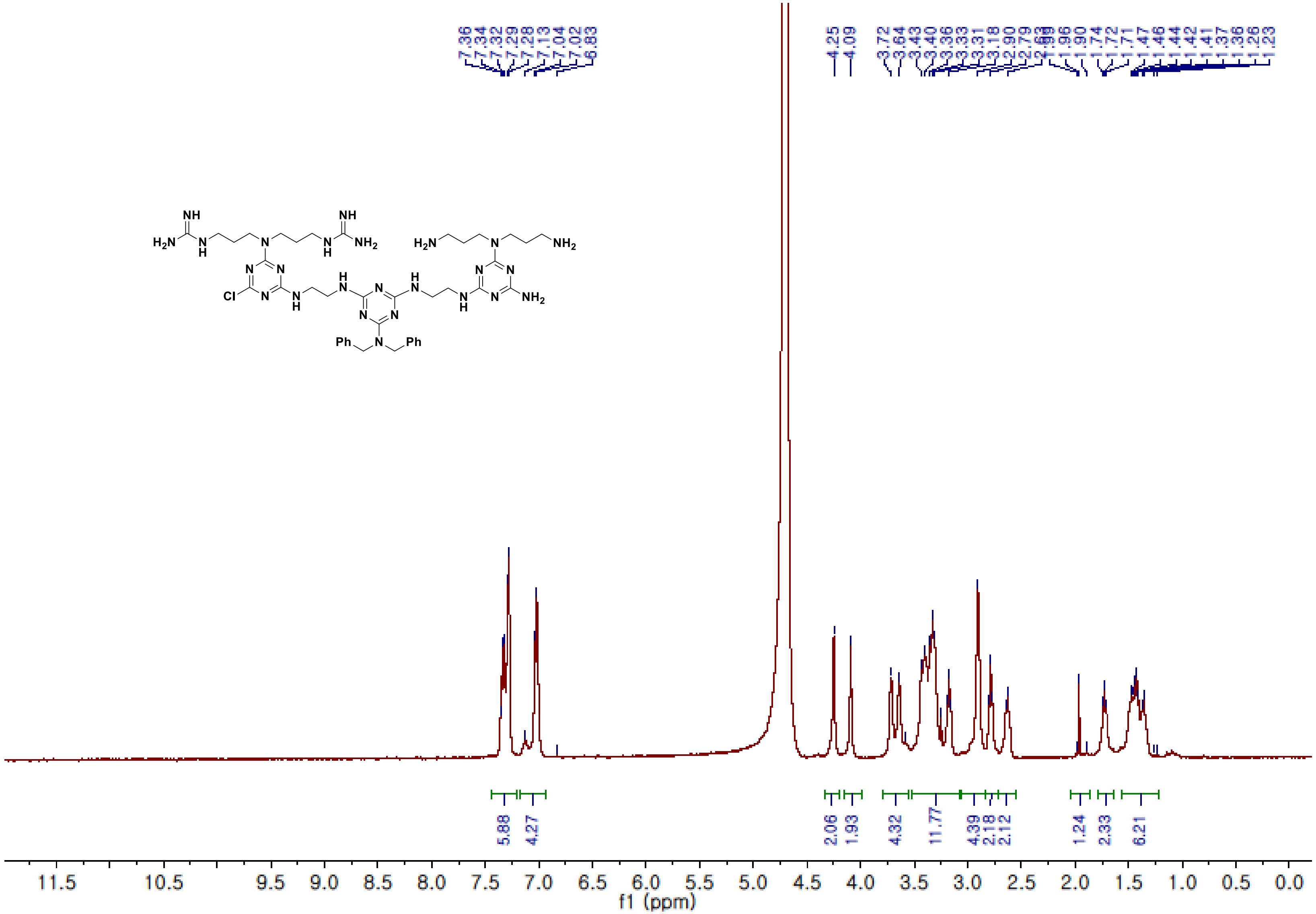

^{1}H NMR spectrum of **TZP6** (D_2O, 400 MHz)

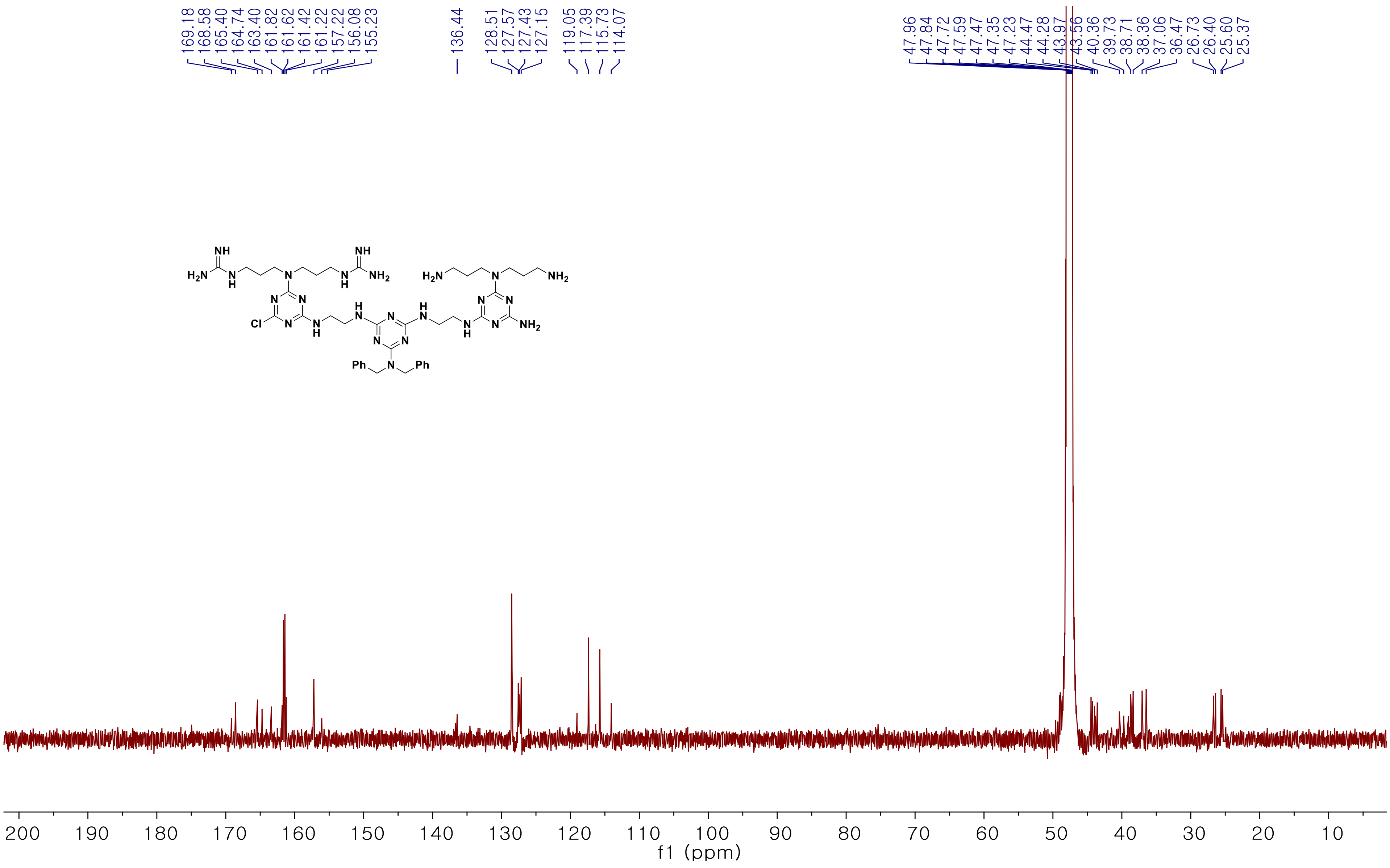

^{13}C NMR spectrum of **TZP6** (MeOD, 176 MHz)

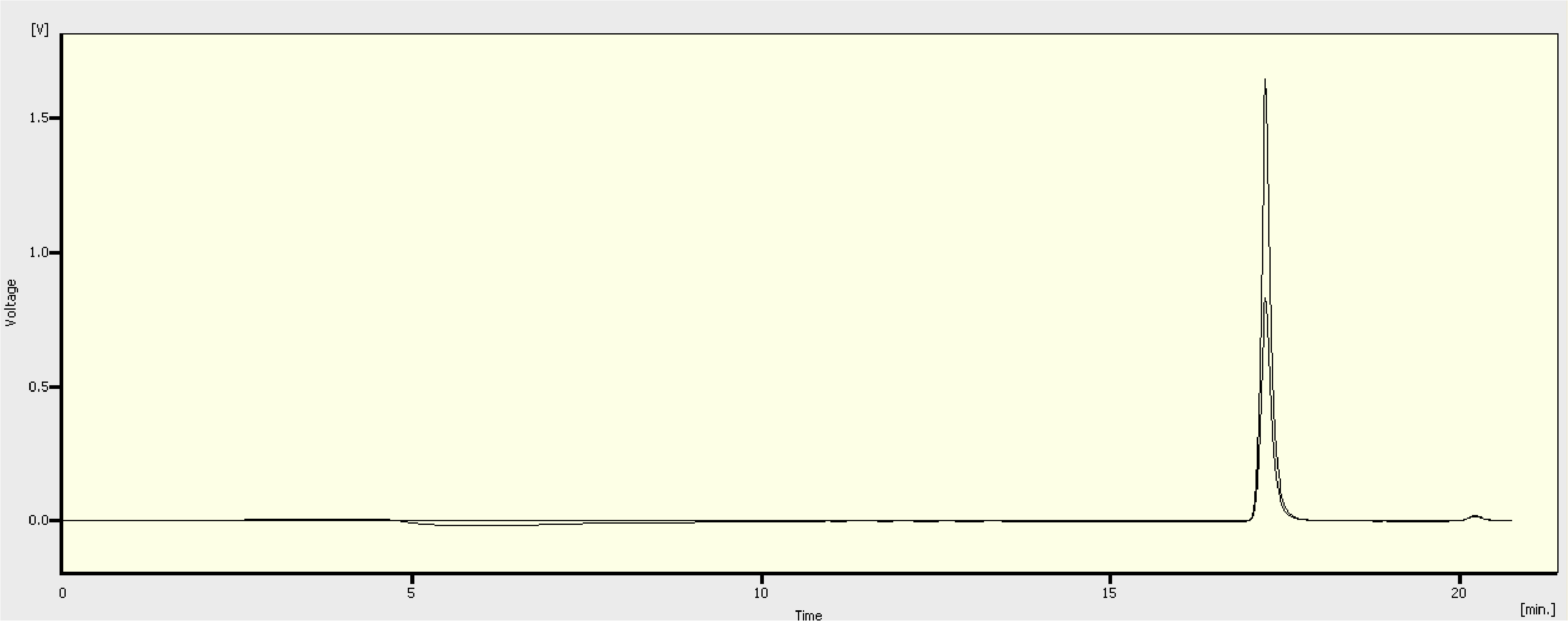

HPLC chromatogram of **TZP6**

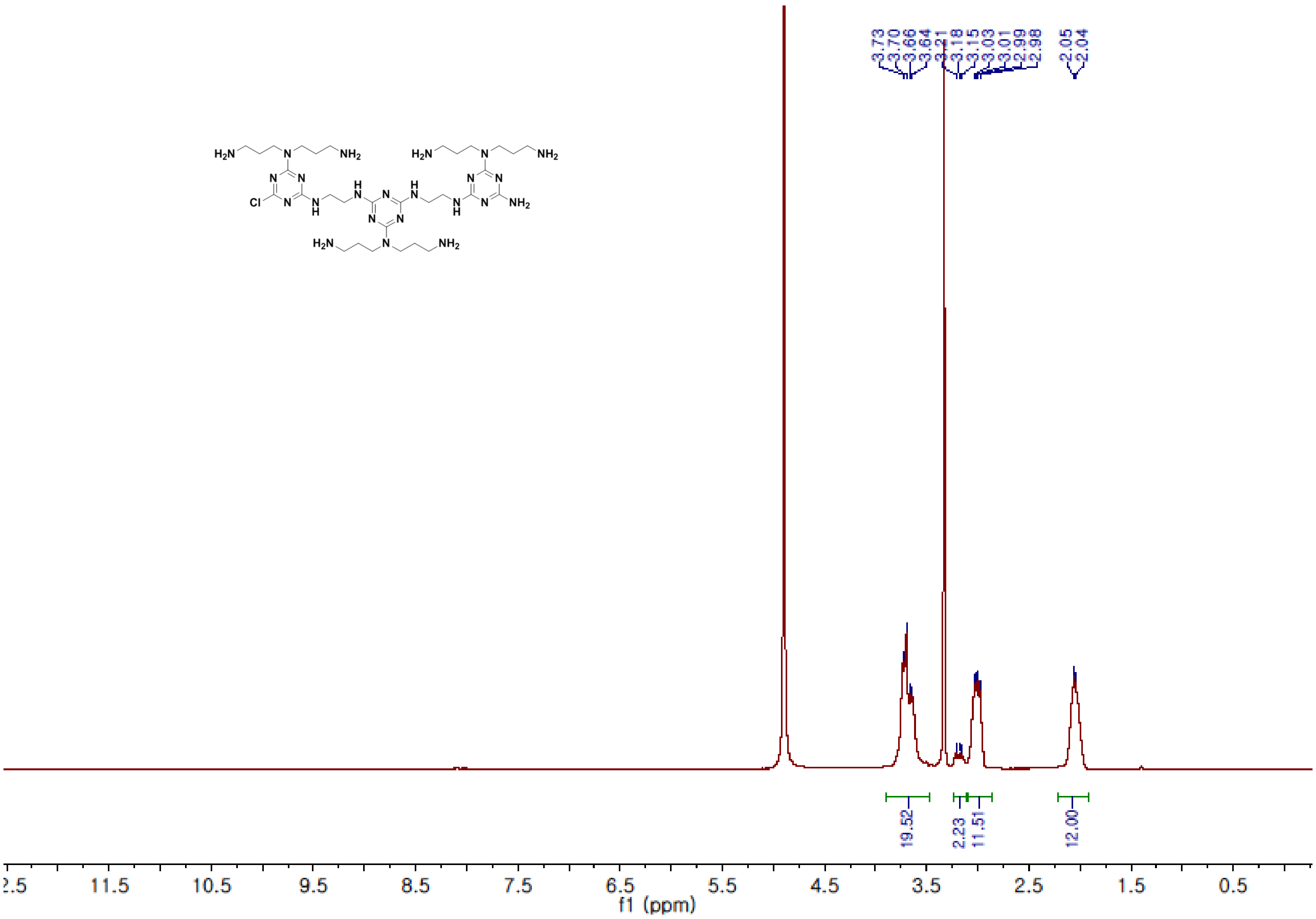

^{1}H NMR spectrum of **TZP7** (MeOD, 400 MHz)

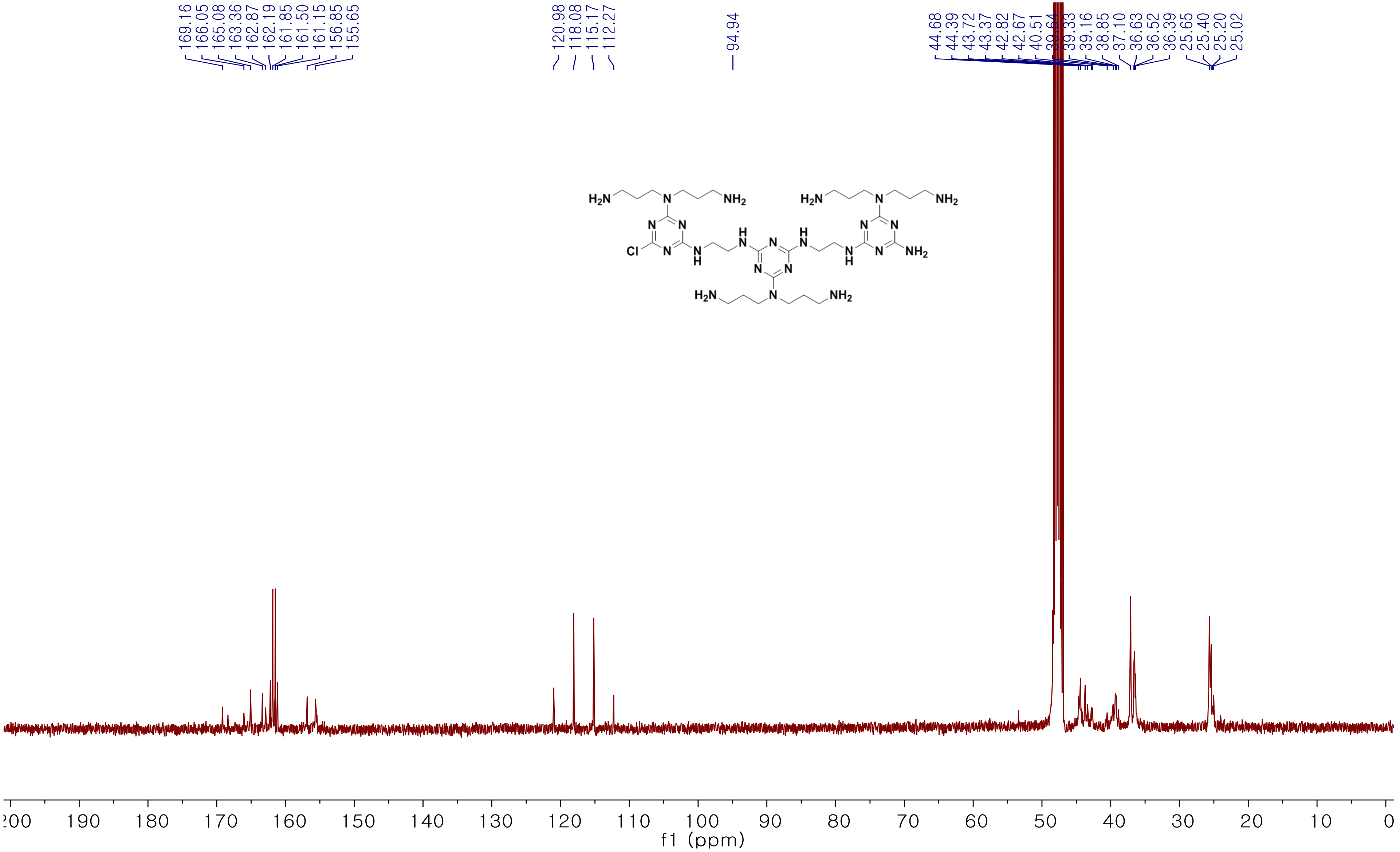

^{13}C NMR spectrum of **TZP7** (MeOD, 101 MHz)

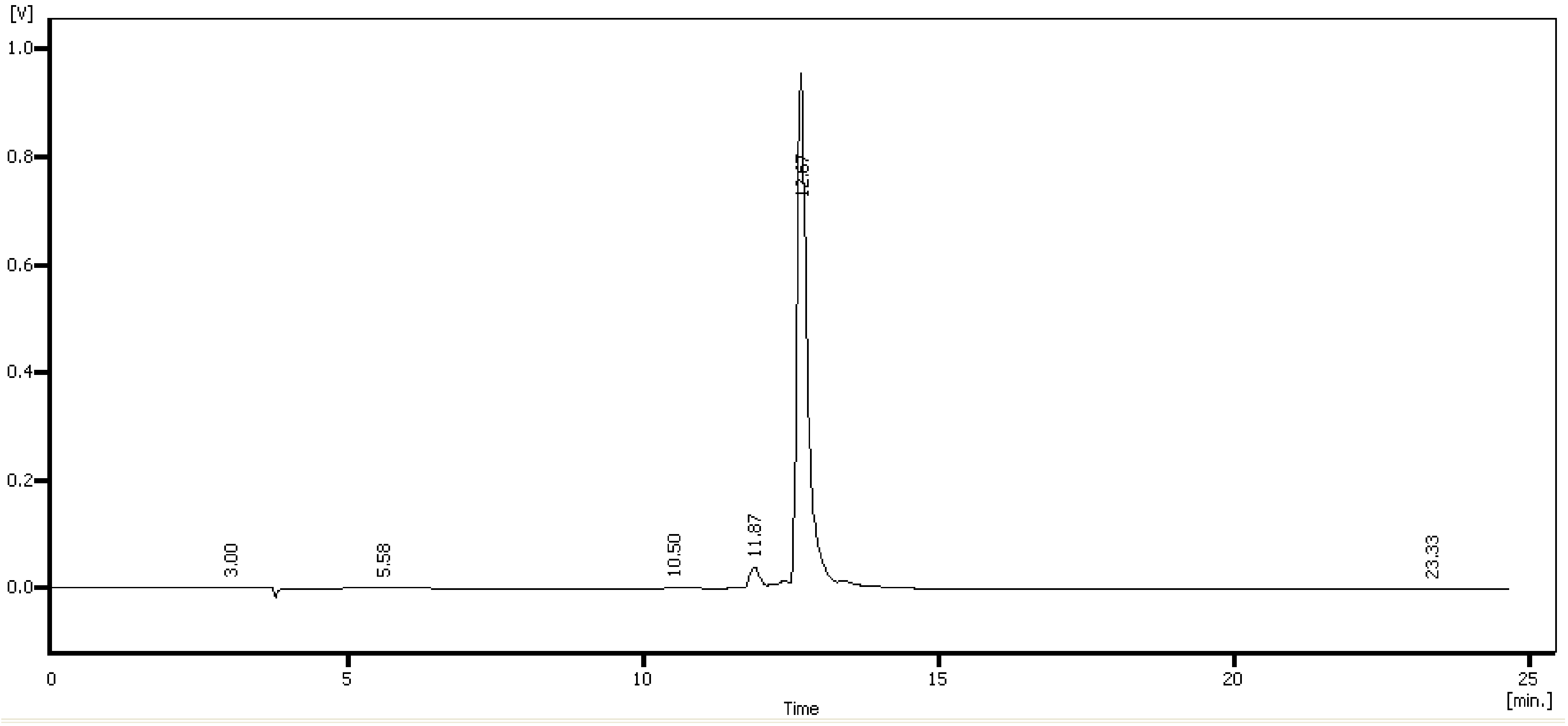

HPLC chromatogram of **TZP7**

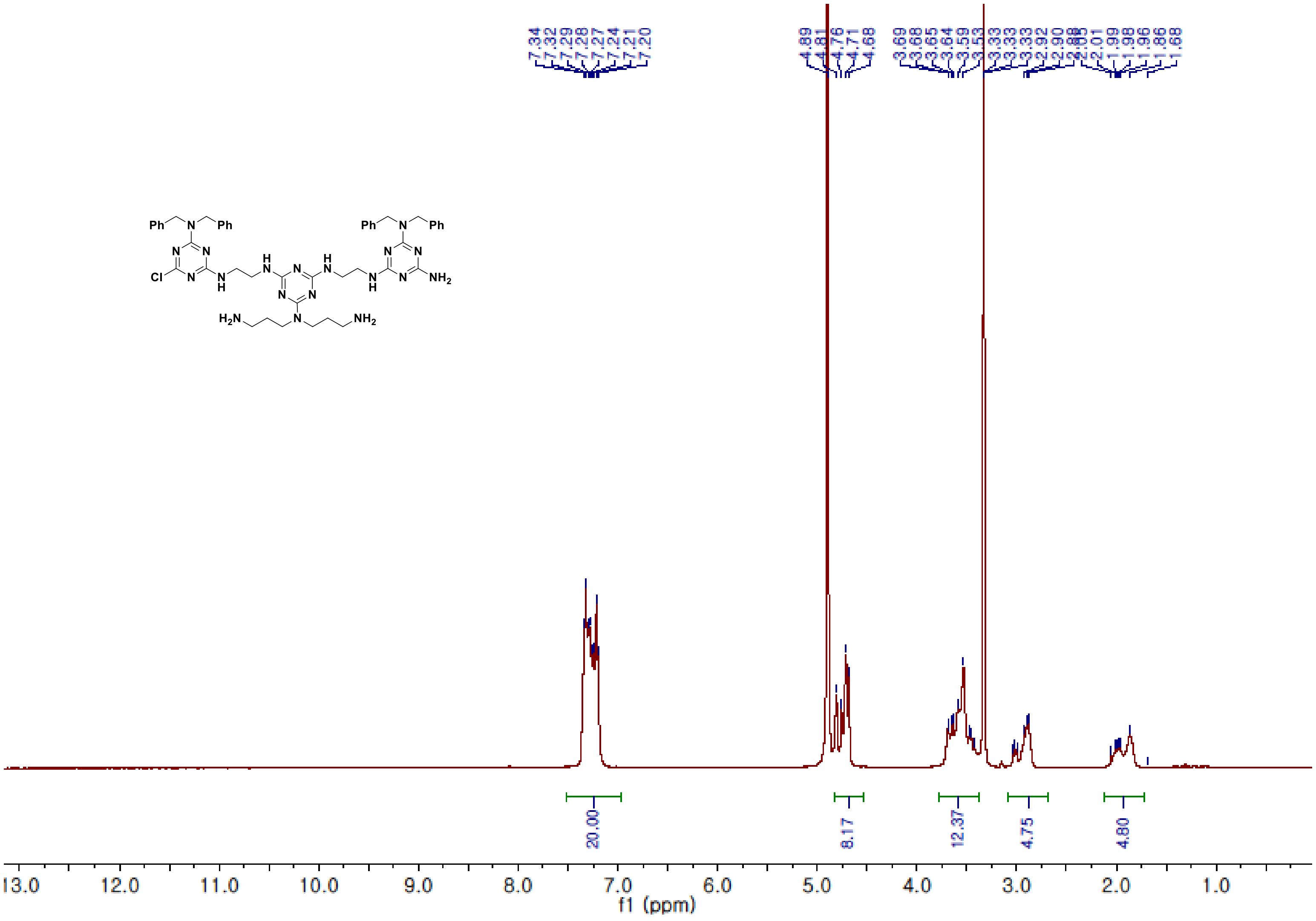

^{1}H NMR spectrum of **TZP8** (MeOD, 400 MHz)

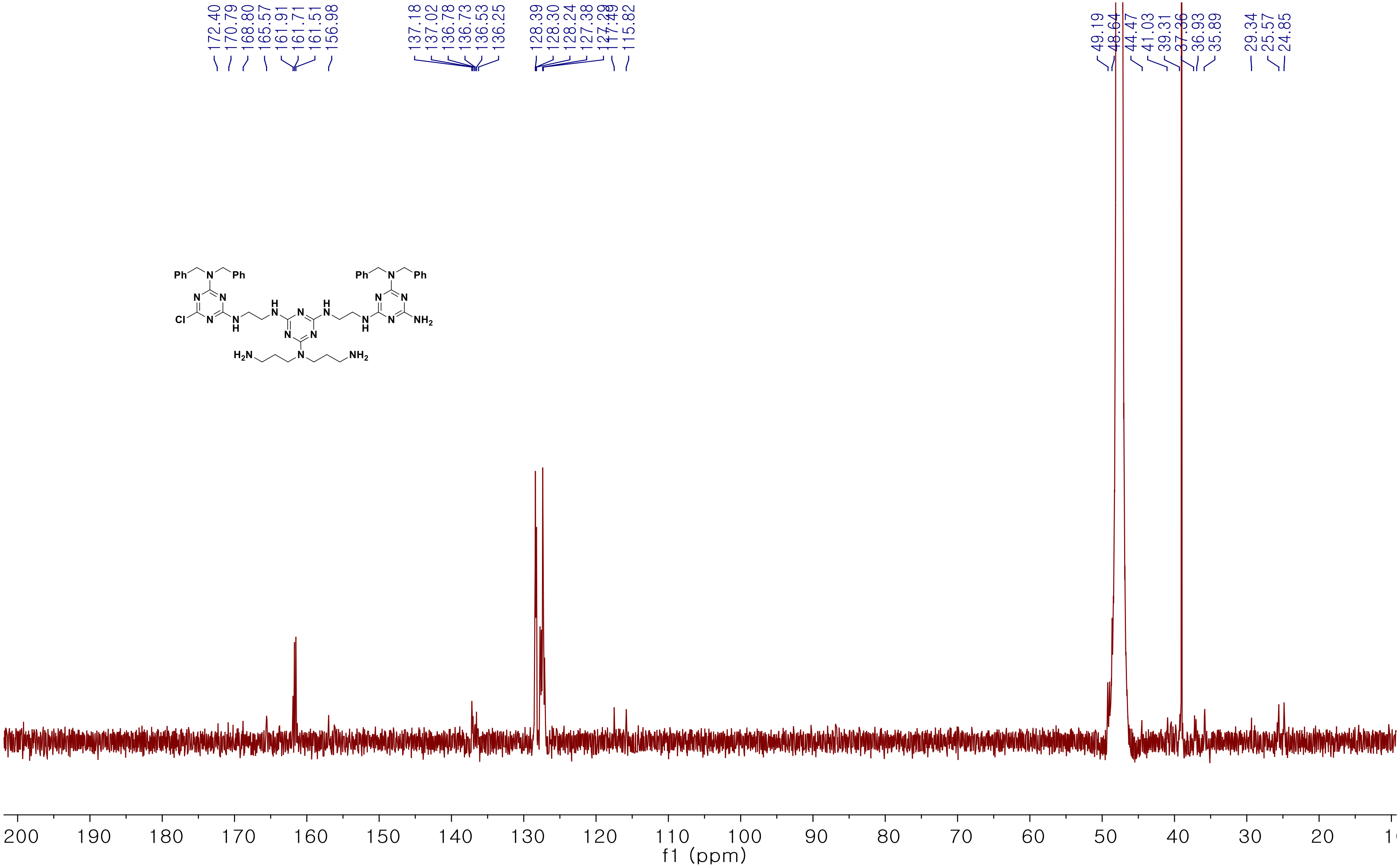

^{13}C NMR spectrum of **TZP8** (MeOD, 176 MHz)

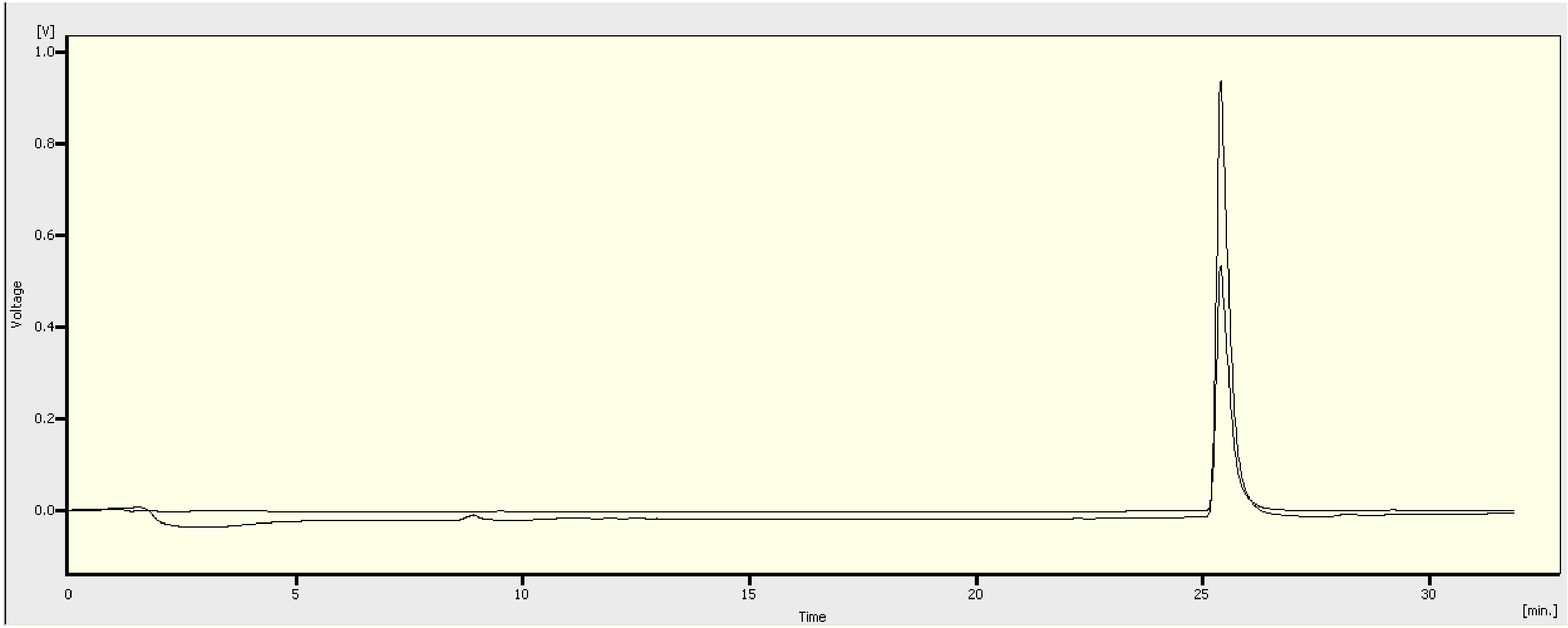

HPLC chromatogram of **TZP8**

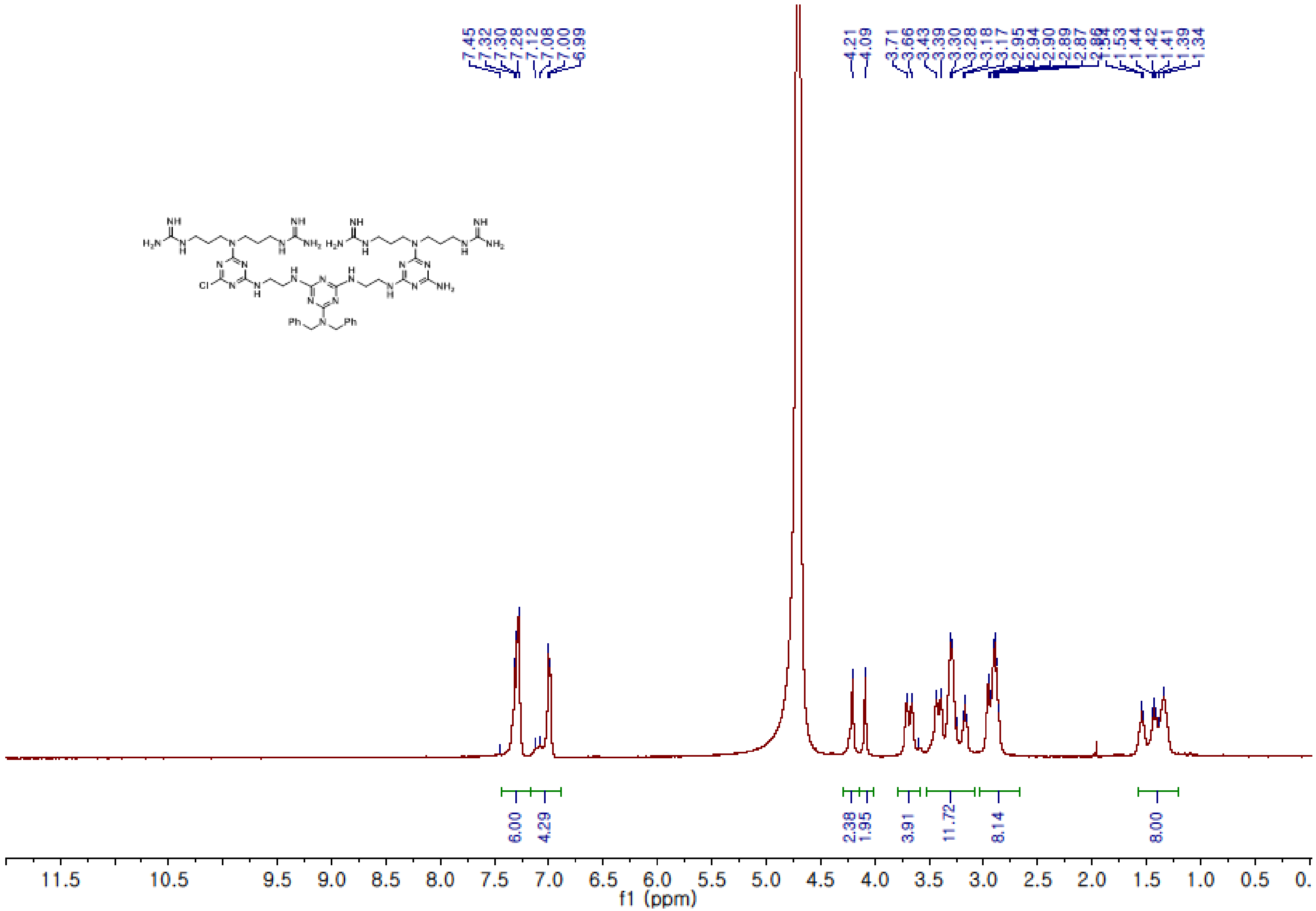

^{1}H NMR spectrum of **TZP9** (D_2O, 400 MHz)

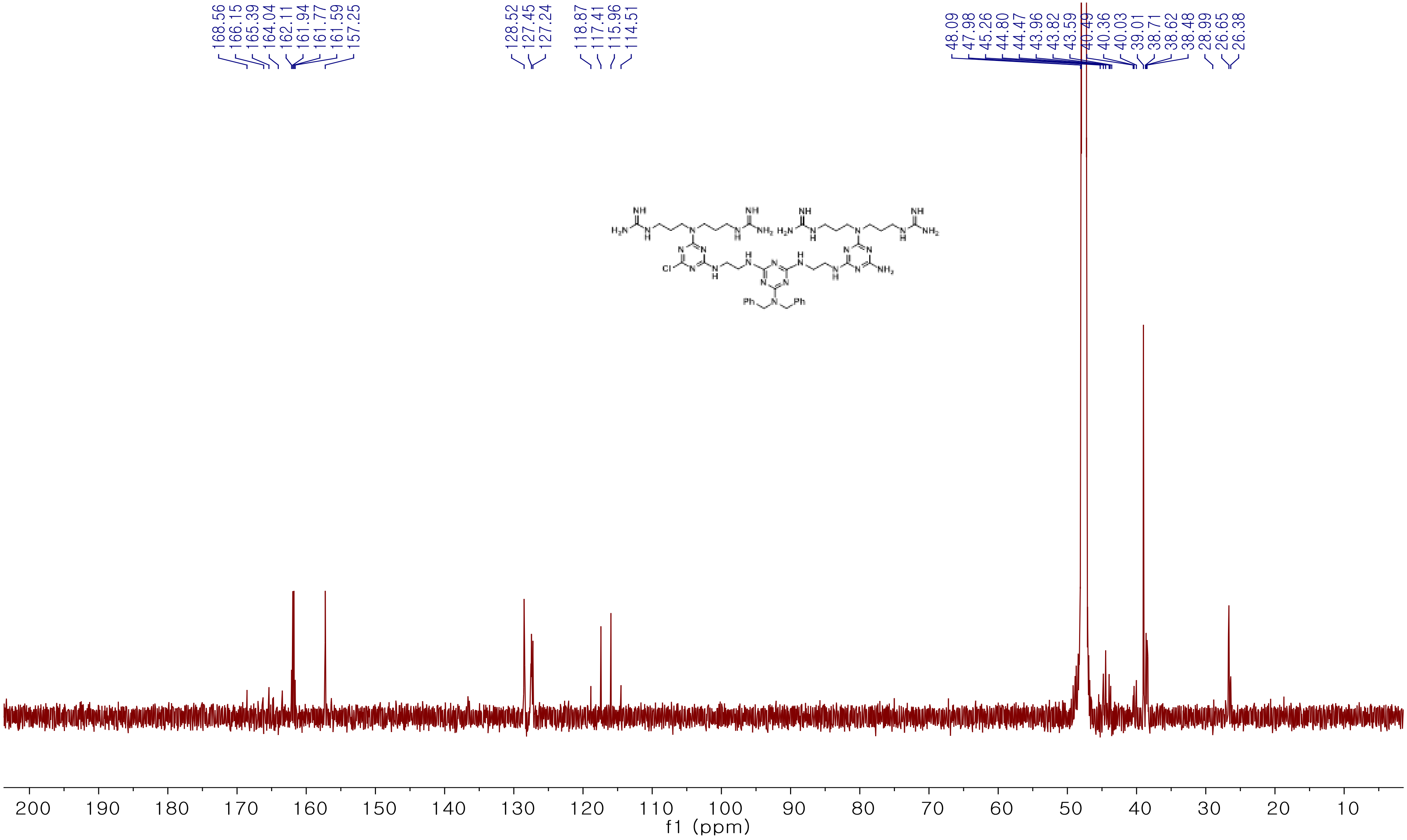

^{13}C NMR spectrum of **TZP9** (MeOD, 201 MHz)

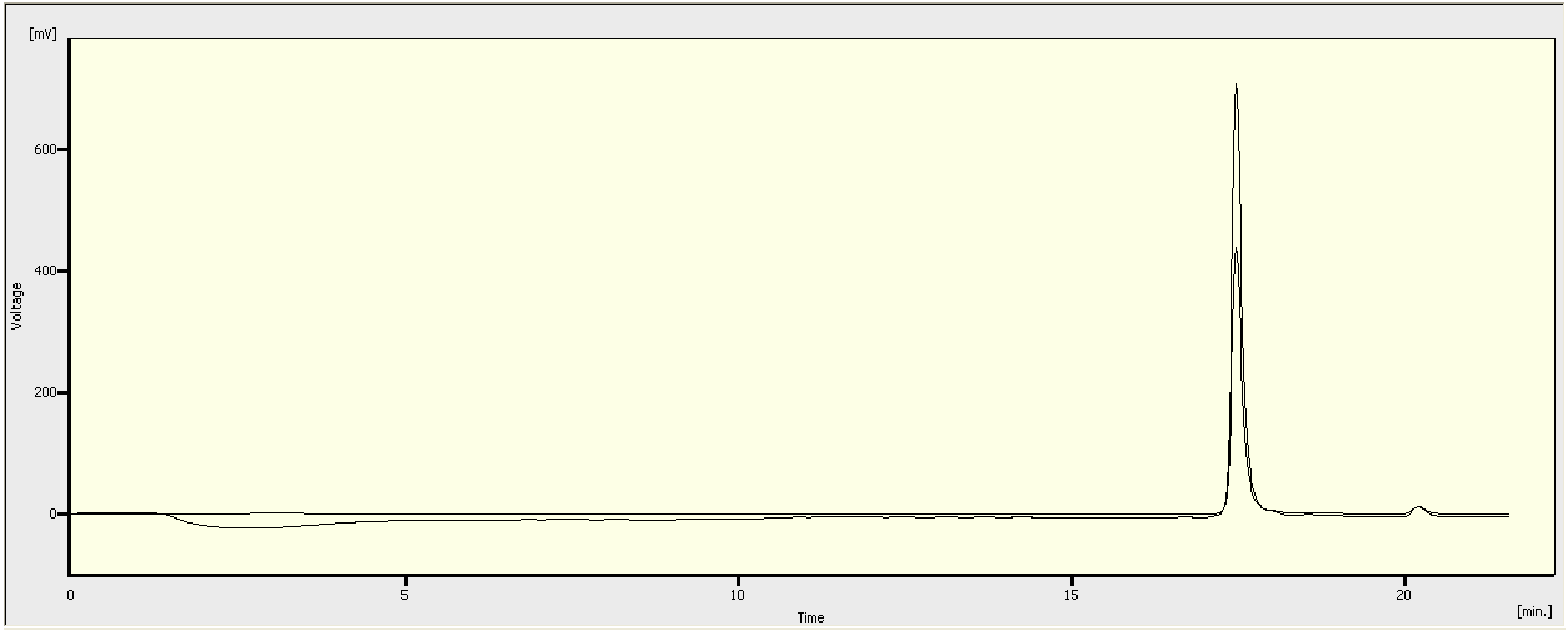

HPLC chromatogram of **TZP9**

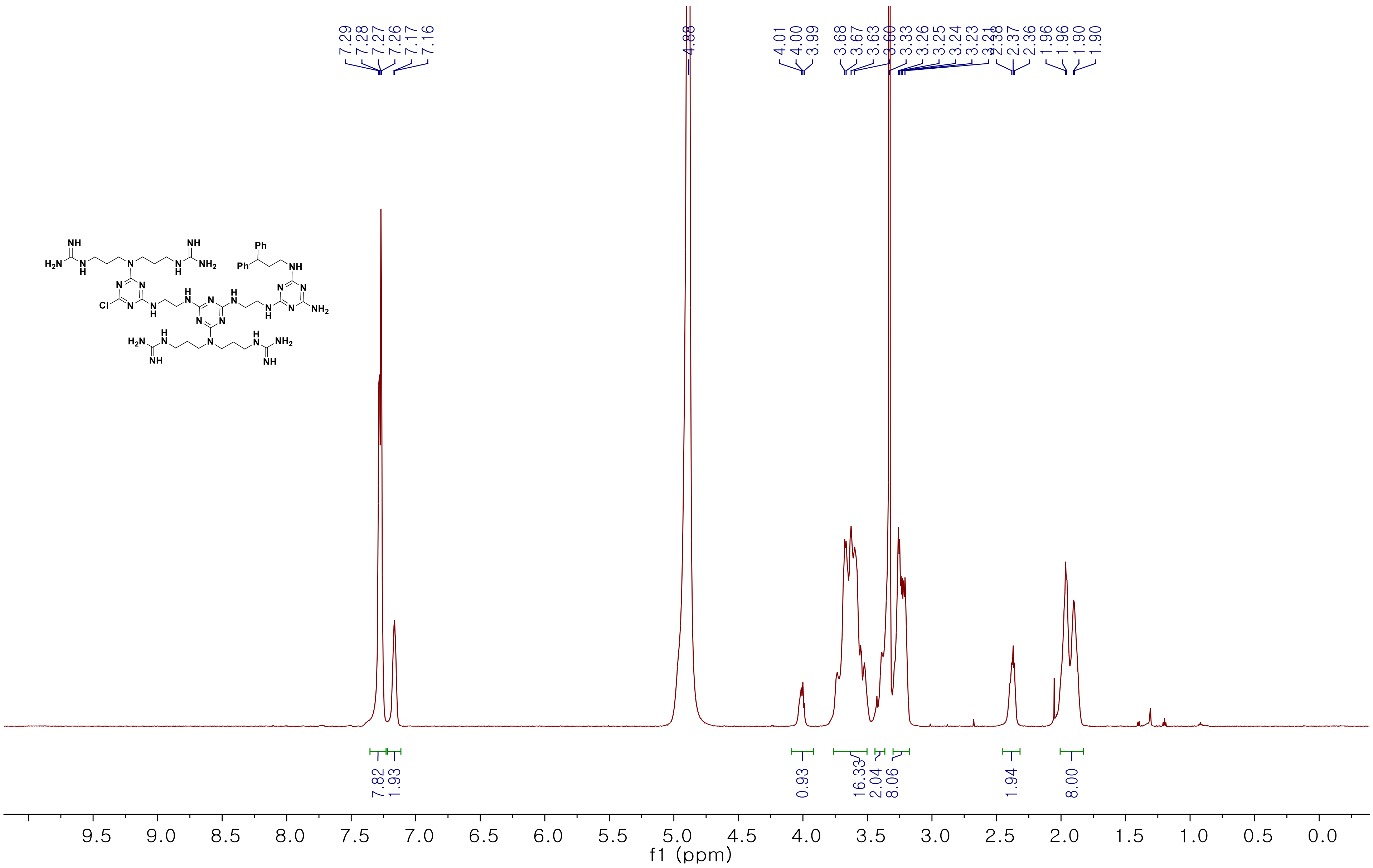

^{1}H NMR spectrum of **TZP10** (MeOD, 700 MHz)

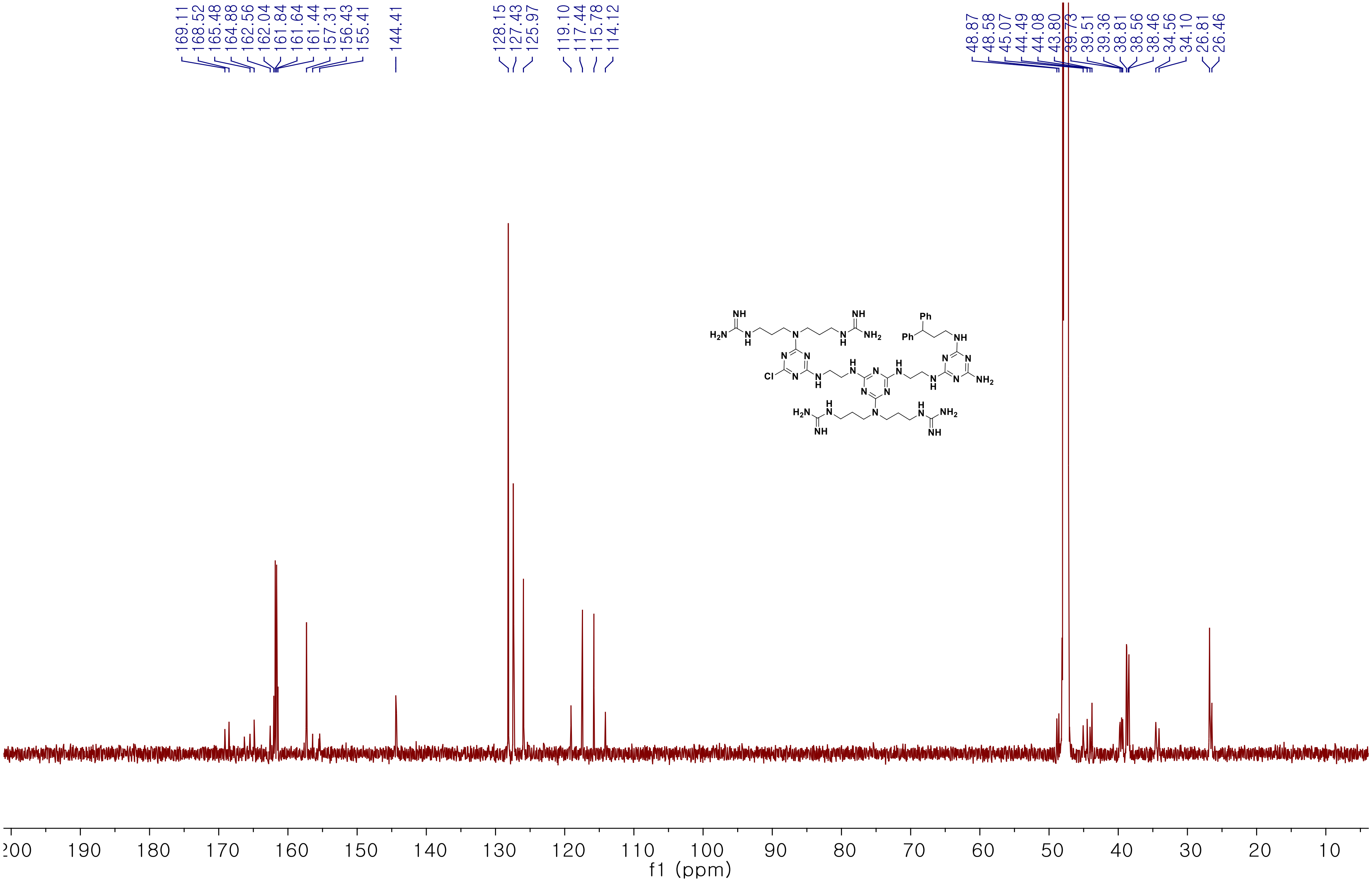

^{13}C NMR spectrum of **TZP10** (MeOD, 176 MHz)

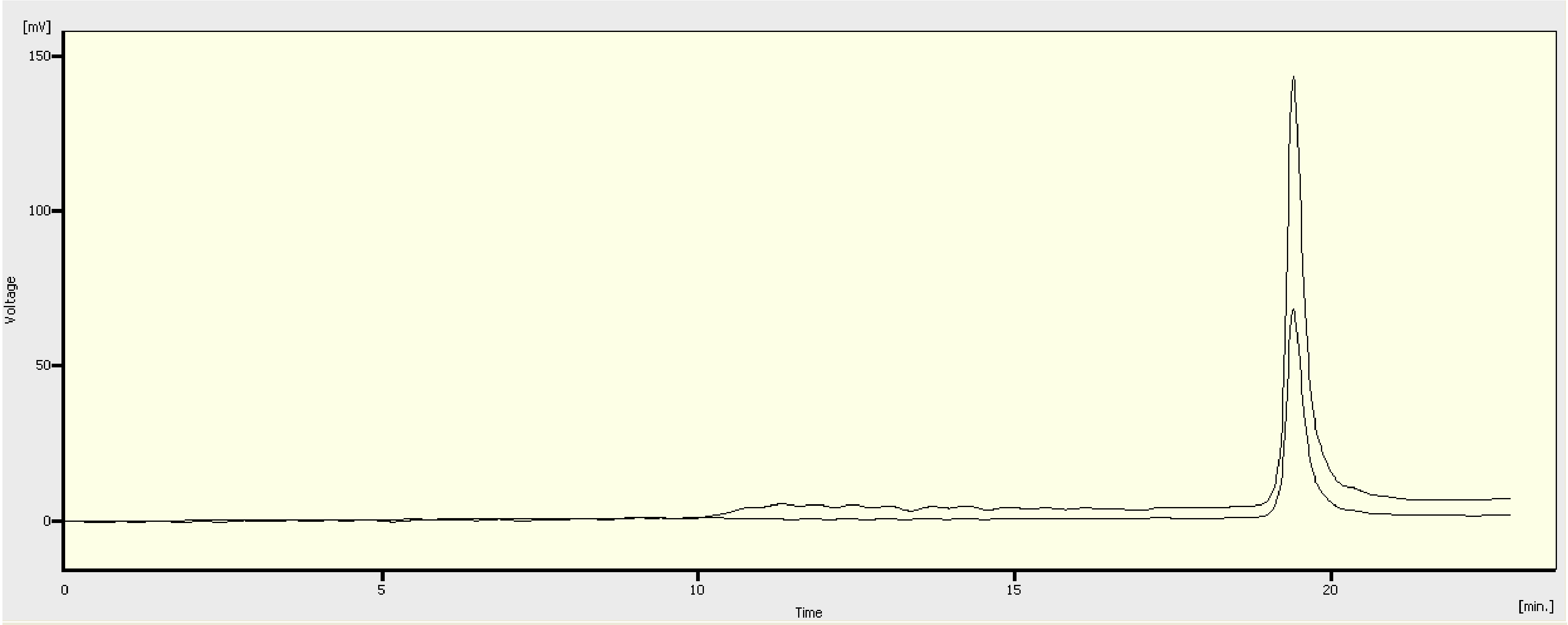

HPLC chromatogram of **TZP10**

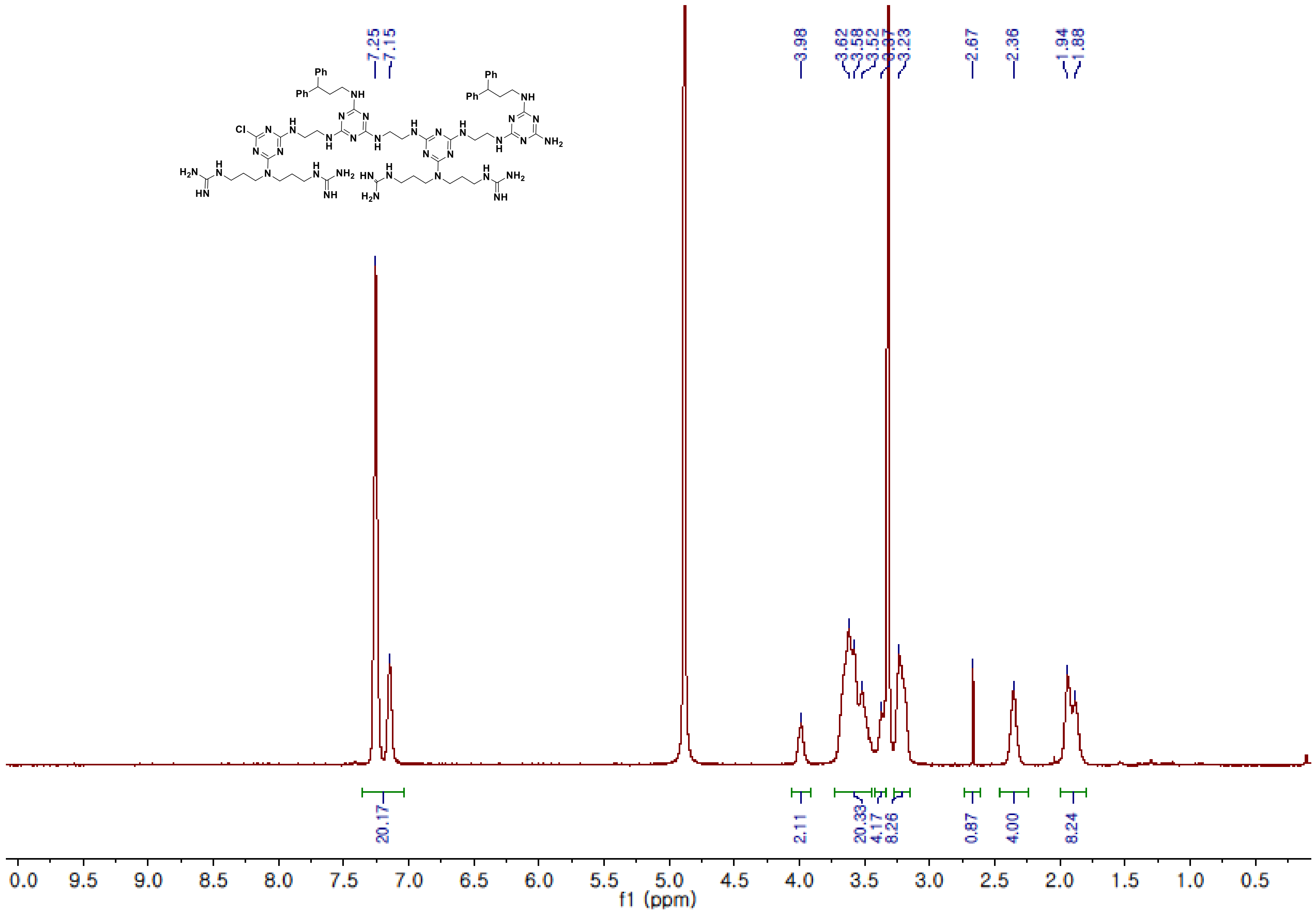

^{1}H NMR spectrum of **TZP11** (MeOD, 500 MHz)

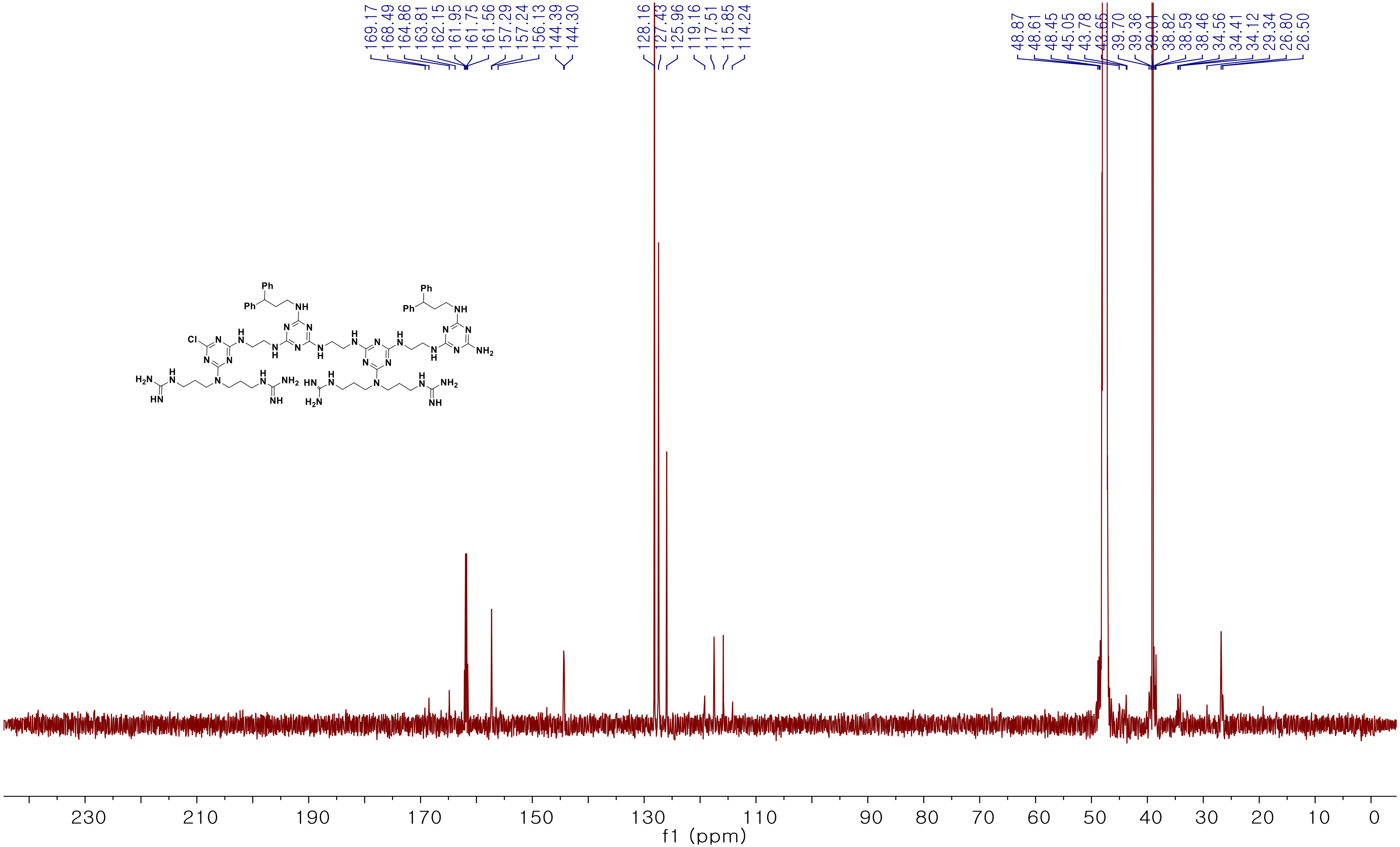

^{13}C NMR spectrum of **TZP11** (MeOD, 176 MHz)

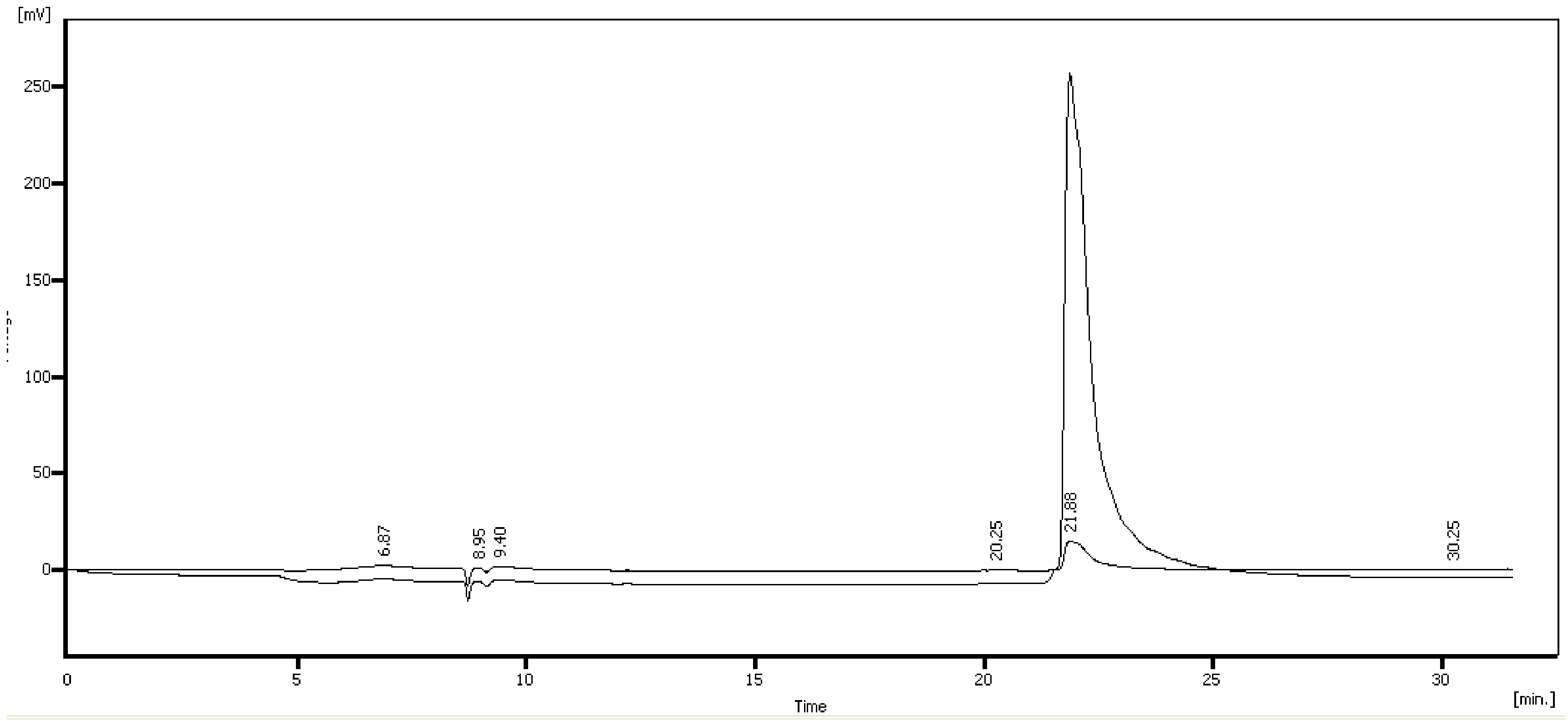

HPLC chromatogram of **TZP11**

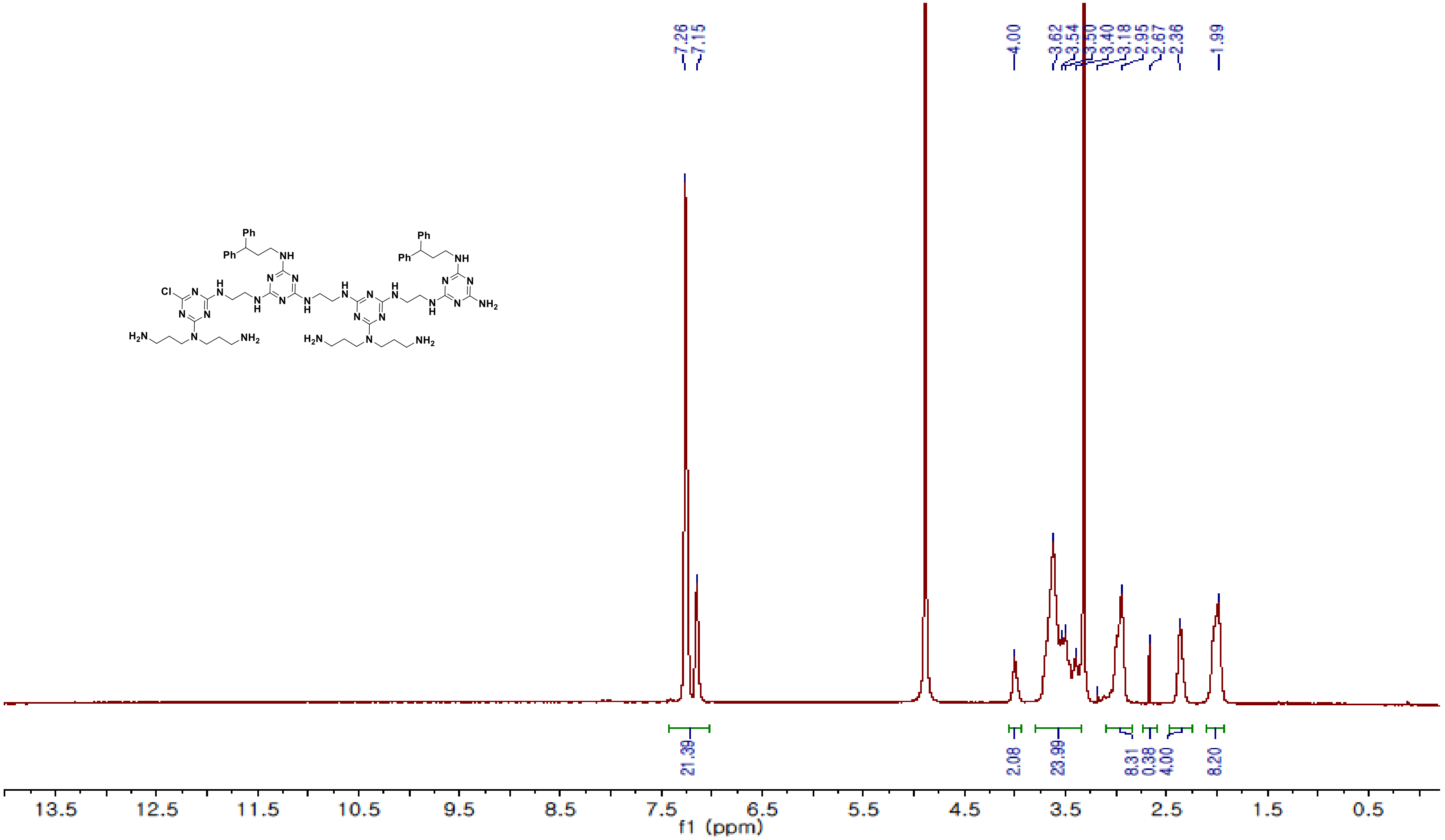

^{1}H NMR spectrum of **TZP12** (MeOD, 500 MHz)

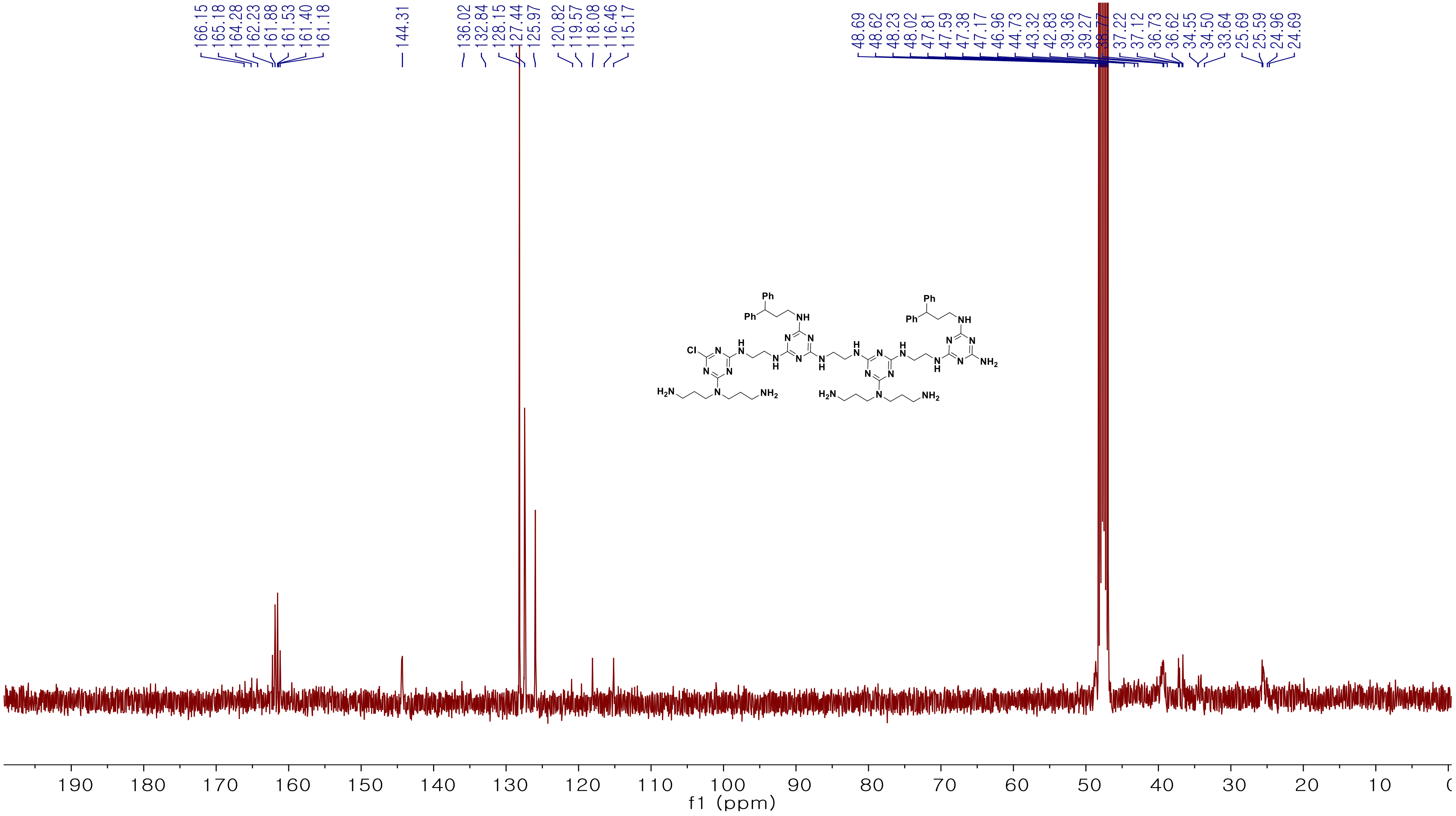

^{13}C NMR spectrum of **TZP12** (MeOD, 101 MHz)

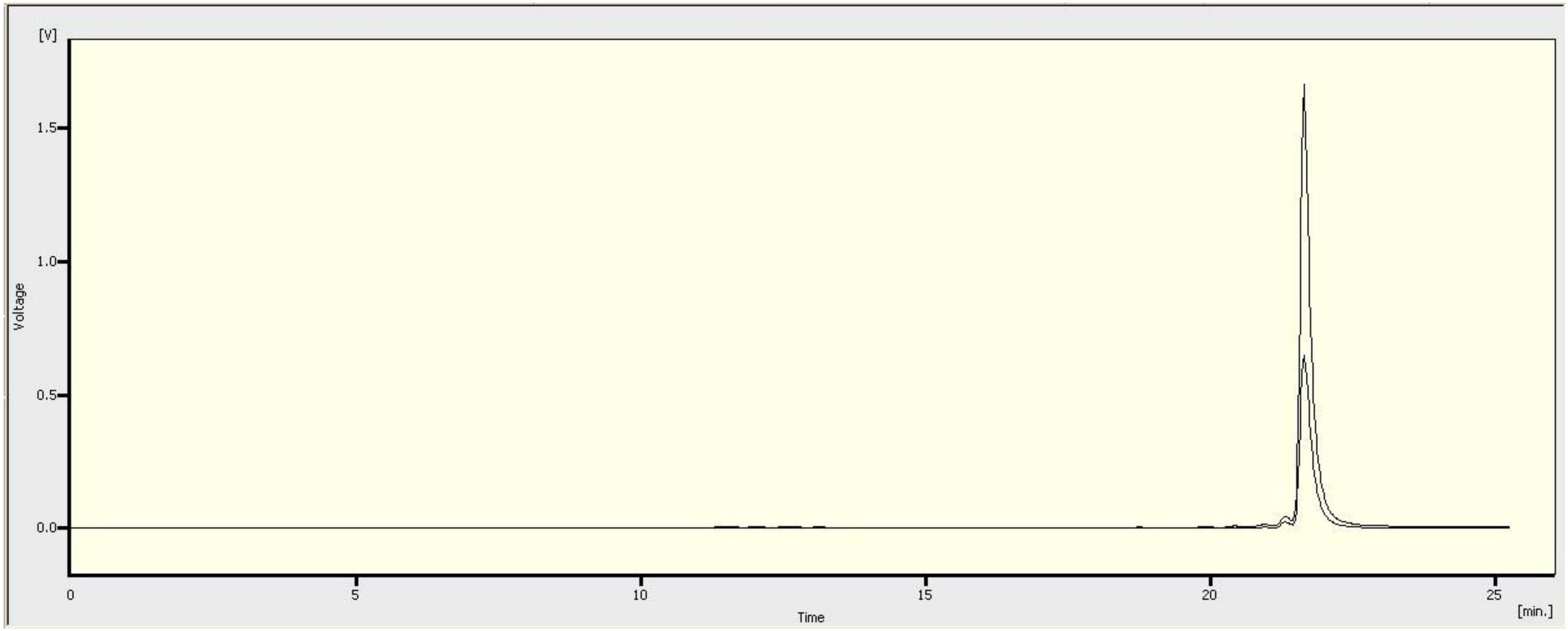

HPLC chromatogram of **TZP12**

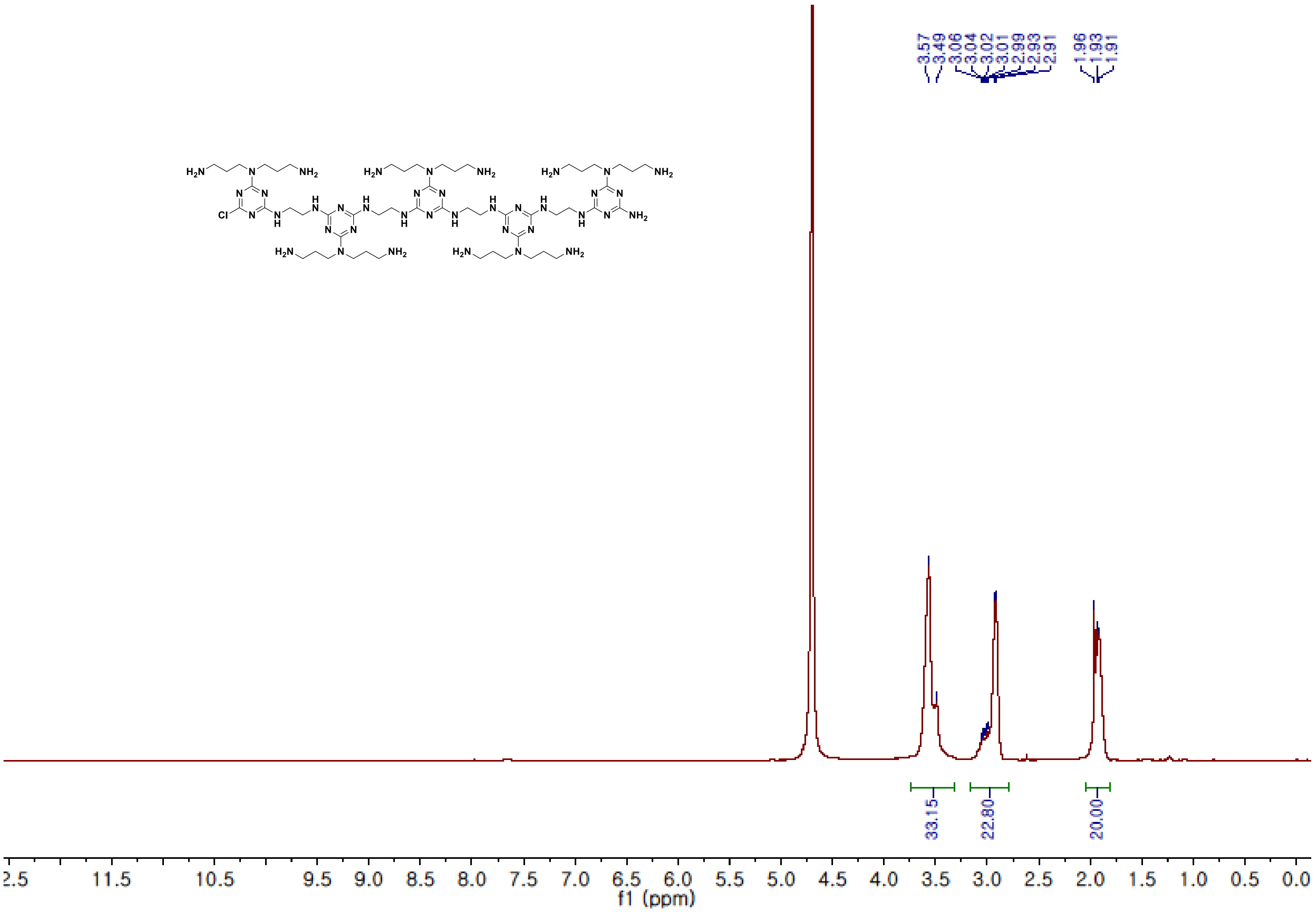

^{1}H NMR spectrum of **TZP13** (D_2O, 400 MHz)

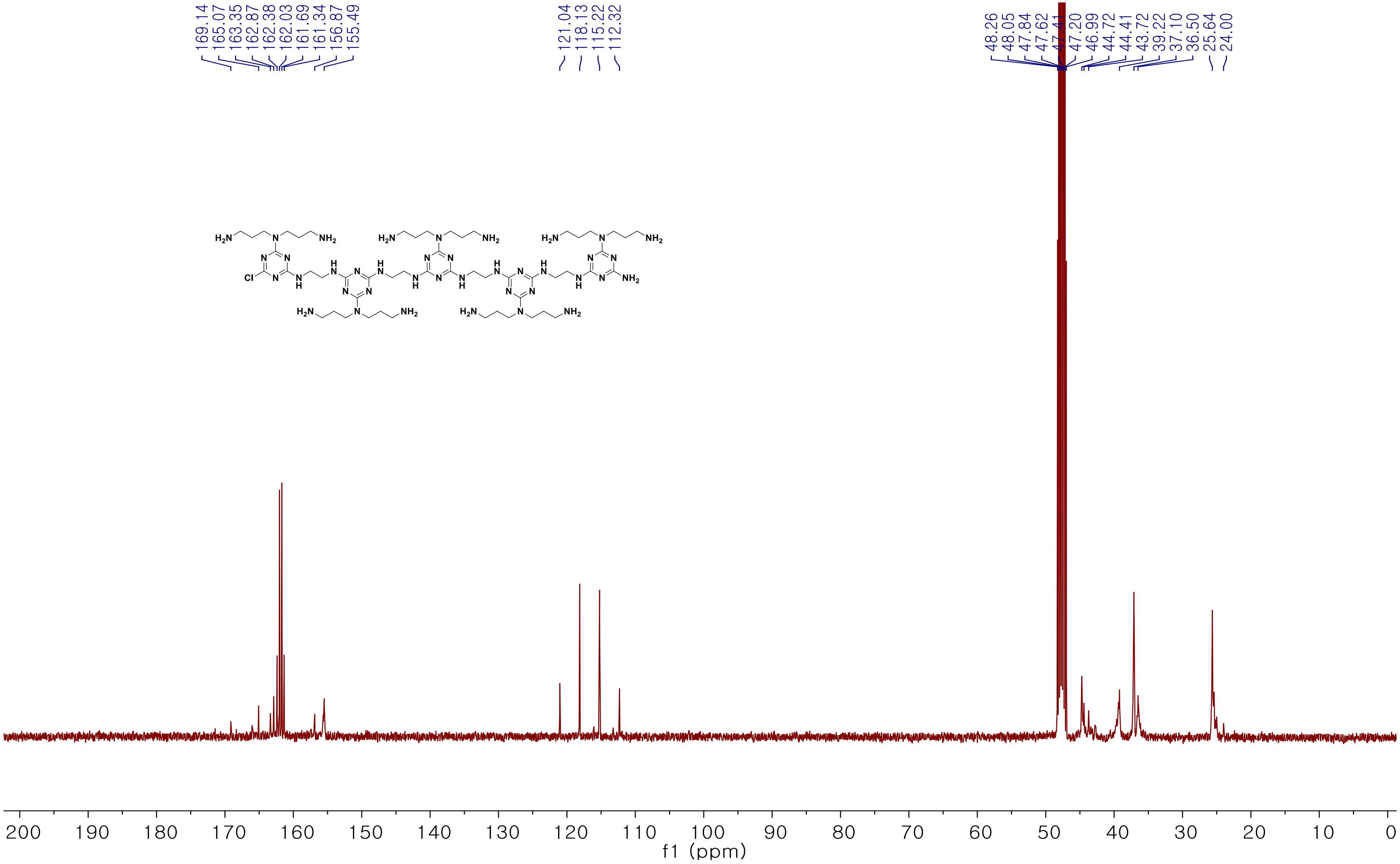

¹H NMR spectrum of **TZP13** (MeOD, 101 MHz)

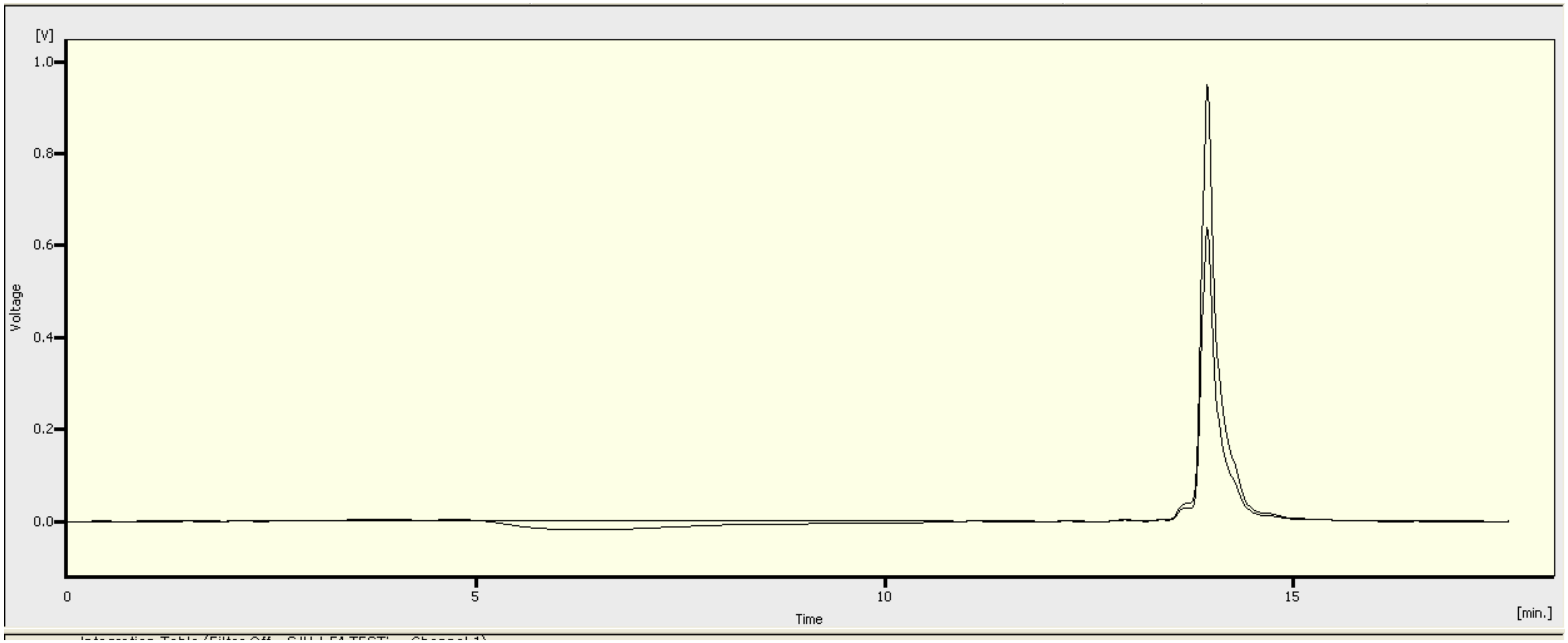

HPLC chromatogram of **TZP13**
